# Integrated analysis of relapsed B-cell precursor Acute Lymphoblastic Leukemia identifies subtype-specific cytokine and metabolic signatures

**DOI:** 10.1038/s41598-019-40786-1

**Published:** 2019-03-12

**Authors:** Michael P. Schroeder, Lorenz Bastian, Cornelia Eckert, Nicola Gökbuget, Alva Rani James, Jutta Ortiz Tanchez, Cornelia Schlee, Konstandina Isaakidis, Björn Häupl, Katharina Baum, Oscar Arturo Migueles Lozano, Khouloud Kouidri, Kuan-Ting Pan, Henning Urlaub, Stefan Schwartz, Thomas Burmeister, Arend von Stackelberg, Dieter Hoelzer, Heike Pfeiffer, Michael A. Rieger, Stefanie Göllner, Thomas Oellerich, Martin Horstman, Martin Schrappe, Jana Wolf, Renate Kirschner-Schwabe, Monika Brüggemann, Carsten Müller-Tidow, Hubert Serve, Martin Neumann, Claudia D. Baldus

**Affiliations:** 1 Charité, University Hospital Berlin, Campus Benjamin Franklin, Department of Hematology and Oncology, Berlin, Germany; 2 German Cancer Research Center (DKFZ), Heidelberg, Germany; 3 German Cancer Consortium (DKTK), Heidelberg, Germany; 4 Charité, University Hospital Berlin, Pediatric Hematology/Oncology, Campus Rudolf Virchow, Berlin, Germany; 5 Goethe University Hospital, Department of Medicine II, Hematology/Oncology, Frankfurt/M., Germany; 6 Max Delbrück Center for Molecular Medicine in the Helmholtz Association, Berlin, Germany; 7 Max Planck Institute for Biophysical Chemistry, Göttingen, Germany; 8 University Medical Center, Göttingen, Germany; 9 University Clinic Heidelberg, Department of Hematology, Oncology & Rheumatology, Heidelberg, Germany; 10 Research Institute Children’s Cancer Center, Dept. of Pediatric Hematology and Oncology, University Medical Center Hamburg, Hamburg, Germany; 11 University Hospital Schleswig-Holstein, Campus Kiel, Department of Pediatrics, Kiel, Germany; 12 University Hospital Schleswig-Holstein, Campus Kiel, Department of Hematology and Oncology, Kiel, Germany; 13 Berlin Institute of Health, Berlin, Germany

## Abstract

Recent efforts reclassified B-Cell Precursor Acute Lymphoblastic Leukemia (BCP-ALL) into more refined subtypes. Nevertheless, outcomes of relapsed BCP-ALL remain unsatisfactory, particularly in adult patients where the molecular basis of relapse is still poorly understood. To elucidate the evolution of relapse in BCP-ALL, we established a comprehensive multi-omics dataset including DNA-sequencing, RNA-sequencing, DNA methylation array and proteome MASS-spec data from matched diagnosis and relapse samples of BCP-ALL patients (n = 50) including the subtypes DUX4, Ph-like and two aneuploid subtypes. Relapse-specific alterations were enriched for chromatin modifiers, nucleotide and steroid metabolism including the novel candidates *FPGS*, *AGBL* and *ZNF483*. The proteome expression analysis unraveled deregulation of metabolic pathways at relapse including the key proteins G6PD, TKT, GPI and PGD. Moreover, we identified a novel relapse-specific gene signature specific for DUX4 BCP-ALL patients highlighting chemotaxis and cytokine environment as a possible driver event at relapse. This study presents novel insights at distinct molecular levels of relapsed BCP-ALL based on a comprehensive multi-omics integrated data set including a valuable proteomics data set. The relapse specific aberrations reveal metabolic signatures on genomic and proteomic levels in BCP-ALL relapse. Furthermore, the chemokine expression signature in DUX4 relapse underscores the distinct status of DUX4-fusion BCP-ALL.

## Introduction

The dismal prognosis of relapsed B cell precursor Acute Lymphoblastic Leukemia (BCP-ALL) warrants for novel and more specific therapeutic approaches^1,2^. Genetic lesions in relapsed BCP-ALL remain the prerequisite for personalized treatment approaches, preferably in the minimal residual disease (MRD) setting^3^. Next generation sequencing (NGS) has advanced the genetic identification of disease-contributing aberrations, which may become targets of new approaches. In Ph-like BCP-ALL, clinical evidence has emerged that patients with these lesions might specifically benefit from treatment with tyrosine kinase inhibitors (TKIs)^4–6^. Besides Ph-like ALL, other molecularly defined subgroups have been unraveled, but yet no targeted therapeutic approaches have been systematically explored^7–9^. For now, molecular studies predominantly focused on pediatric ALL and on molecular alterations at initial diagnosis^7,10–13^.

An important prognostic factor in pediatric and adult relapsed ALL is the duration of first remission^14,15^. Identified differences in the molecular make-up of early and late pediatric relapses are *CDKN2A/2B* deletions, which are more frequent in early relapses, and *NT5C2* mutations occurring primarily in early relapses/on treatment^3,16^. Gene expression variations associated with time to relapse as well as a great diversity in the IG/*TCR* gene rearrangement repertoire in early relapse have been reported^17–20^, suggesting a different molecular portrait and a distinct pattern of clonal evolution in early versus late ALL relapse.

Clonal evolution studies have revealed mutations emerging in subclones, different from the dominant diagnostic clone^21,22^. The first comprehensive study, which analyzed relapse-specific genetic alterations, identified recurrent mutations in *CREBBP*, a transcriptional corepressor^23^. Other studies identified *NT5C2*, *RAS* and *PRPS1* mutations emerging in novel clones in relapsed pediatric ALL^16,24,25^. Particularly *NT5C2* mutations have been described to emerge as a response to chemotherapy^16^. The heterogeneity of cancer makes it likely that additional mutated driver genes will be discovered in sub-entities that have not yet been studied in depth^26^.

In contrast to the availability of detailed genomic data generated on NGS platforms, proteomic characterization of BCP-ALL remains largely unexplored. Recent insights though highlight the relevance of proteomic and metabolomic analyses demonstrating the gatekeeper function of the Pentose-Phosphate pathway (PPP) in PAX5- and IKZF1-driven BCP-ALL mouse models and other model systems^27^. Yet unbiased proteomics on primary samples from relapsed BCP-ALL patients combined with matched multi-omics data are lacking.

Thus, here we combined pediatric and adult relapsed BCP-ALL in one dataset for a comprehensive approach, analyzing DNA methylation, RNA- and exon-sequencing and proteome expression data obtained from the same samples in order unravel relevant pathway alterations. This multi-omics characterization of a combined cohort of 50 matched triplicate samples at diagnosis, remission and relapse highlight novel insights in key mechanisms of resistance.

## Material and Methods

### Patients samples

All patients were treated in population based German study trials (GMALL for adult and COALL/BFM for pediatric patients). All patients gave written informed consent to participate in these trials according to the Declaration of Helsinki. This study was approved by the ethics board of Charité, Berlin. Patients sample triplets retrieved at initial diagnosis (ID), complete remission (CR) and relapse (REL) excluded patients with known fusion genes (BCR-ABL1, KMT2A-AFF1, ETV6-RUNX1). CR samples were used as germline controls for whole exome and panel sequencing. Pediatric and adult patients treated on pediatric inspired intensive protocols were categorized into early and late relapse, based on a cut-off at 700 days to relapse.

### Nucleic acid preparation

RNA isolation was performed using Trizol reagent (Life Technologies, Grand Island, NY). RNA integrity numbers greater than seven were required. Samples from ID and REL were used for RNA-seq. DNA was extracted using unstranded Allprep extraction (Qiagen, Hilden, Germany) and used for WES, panel-sequencing and methylation arrays. For WES, samples from ID, CR, and REL were processed.

Sequencing was performed on an Illumina HiSeq4000 platform. For Whole Exome Sequencing (WES) three samples/lane were proceeded using Low input Exome-Seq Human v5 + UTRs (Agilent, Santa Clara, California) with an average coverage of 141.6 Mio mapped reads/sample (MMRS). Panel-sequencing was performed using a customized biotinylated RNA oligo pool (SureSelect, Agilent, Santa Clara, California) to hybridize the target regions comprising 362 kbp on a HiSeq2000. We obtained an average coverage of 30.1 MMRS. For RNA-seq, six samples per lane were sequenced with an average 64 MMRS. All sequences were aligned to the human genome build GRCh37.75^28^ using the bcbio-nextgen pipeline v0.9.1a-7da8dce and STAR-aligner^29^ respectively.

Protein expression was obtained by using an UltiMate 3000 RSLCnano HPLC system coupled online to a Q Exactive Plus mass spectrometer. A detailed protocol is available in Supplementary Methods.

Primary data are available at the European Genome-phenome Archive (EGAS00001002856).

Somatic mutations were detected using the bcbio-nextgen pipeline Mutect, Freebayes, Vardict, Varscan, copy number variations were called with CNVkit and copywriteR; Pyclone and Schism were used for the clonality analyses. Fusion genes were detected with defuse and FusionCatcher and expression quantification were obtained with Stringtie; differential expression analysis was performed with limma. Differential methylation analysis has been performed with bumphunter. Statistical Tests were carried out two-tailed and if not indicated otherwise in Supplementary Methods^30–38^.

### Ethics approval and consent to participate

All patients gave written informed consent to participate in these trials according to the Declaration of Helsinki.

## Results

### Genomic characterization of adult and pediatric relapsed patients

We analyzed 50 BCP-ALL patient trios, initial diagnosis (ID), complete remission (CR) and relapse (REL), including 26 pediatric and 24 adult patients lacking recurrent cytogenetic rearrangements as assessed by the conventional diagnostic workup (*BCR-ABL1*, *KMT2A-AFF1*, *ETV6-RUNX1*, *TCF3-PBX1*). We used the WES data of the CR samples as germline control for the mutation and copy number analyses. The mutational and copy number status were examined by WES and targeted panel sequencing; expression profiles and fusion-genes were obtained via RNA-sequencing and the methylation status by Illumina DNA Methylation arrays.

We profiled the recurrently mutated genes and targets of copy number alterations (CNA). Epigenetic regulators (*CREBBP*, *KMT2D*, *EZH2*; Table S1a,b; Fig. S1) were predominantly altered in adult patients. In contrast, mutations in genes related to the activity of conventional ALL therapy elements (*NR3C1* - glucocorticoid response, *NT5C2* - response to purine analogues) were only observed in pediatric patients (Table 1). Patients relapsing early had more alterations in *CDKN2A/B* and in *PTPRD*, whereas patients relapsing late showed more *IKZF1* alterations (Table 1). Genes preferentially subjected to homozygous deletions were *VPREB1* (n = 6), *SH2B3* (n = 4), and *ETV6* (n = 2). The *SH2B3* deletions occurred exclusively in pediatric samples.

**Table 1:** Molecular events biased towards subgroups. Samples from early relapse (ER; time of REL <700 days) showed a bias towards deletions involving the CDKN2A/B locus as 13 of the 15 deletions were associated with an early relapse as well as mutations or copy number losses of PTPRD (7/7).

Gene	Early Relapse (ER) Patients (n = 25)	Late Relapse (LR) Patients (n = 25)	p-value
*CDKN2B*	13 deletions	2 deletions	<0.01
*CDKN2A*	13 deletions	3 deletions	<0.01
*PTPRD*	7 (6 deletions/1 mutation)	0 alterations	<0.01
*PRSS3*	8 (7 deletions/1 mutation)	1 deletion	0.02
*IKZF1*	2 (1 deletion/1 mutation)	9 (4 mutations/3 deletions/2 double hits)	0.04
	**Pediatric Patients (n** = **26)**	**Adult Patients (n** = **24)**	
*NR3C1*	7 (6 deletions/1 mutation)	0 alterations	0.01
*CREBBP*	1 deletion	7 (5 mutations/2 deletions)	0.02
*KMT2D*	1 mutation	6 mutations	0.05
*EZH2*	0 alterations	4 (2 mutations/2 deletions)	0.05
*TP53*	3 (2 double hits/1 mutation)	8 (3 double hits/3 mutations/2 deletions)	0.09
*NT5C2*	4 mutations	0 alterations	0.11

Mutations and copy number losses of IKZF1 were observed in 9 of 11 patients with IKZF1 alterations in ID and/or REL relapsed later than 700 days and thus revealed a clear tendency to late relapse. NR3C1 was deleted (n = 6) and mutated (n = 1) exclusively in pediatric samples. Acronyms used: ER (early relapse), LR (late relapse), ID (initial diagnosis), REL (relapse).

### Transcriptome and methylome signatures classify samples into BCP-ALL subtypes

We classified the samples into known molecular BCP-ALL subtypes based on distinct mRNA expression signatures accompanied by specific gene fusions, mutations and a defined methylation status (Fig. S2a–c; Supplementary Table S2a–d). This subgroup allocation remained stable from diagnosis to relapse. 12 patients (24 samples), including 10 pediatric, were assigned to the *DUX4*-*IGH* fusion (short: DUX4) subtype, 12 patients (24 samples) were classified as Ph-like and 14 patients (28 samples; 4 pediatric, 10 adult) had an aneuploid karyotype defined by 3 or more whole chromosomes affected by loss of heterozygosity (LOH; loss of chromosome or copy-neutral LOH) or other hyperdiploidies (Table S3a). Of these 14 aneuploidy cases, 4 patients had TP53 mutations accompanied by a masked low-hypodiploid karyotype (LH) including gains in chromosomes 1 and 22 (Fig. S3). The remaining 10 aneuploid patients shared distinct gene methylation and expression patterns (Fig. S2a,b) and were classified as masked near-haploid ALL and high hyperdiploid, thus referred to as NH-HeH. NH-HeH patients showed a pattern of gains in chromosomes 4, 14 and 21 (Table S3a,b). The analysis of differentially expressed and methylated genes revealed that DUX4 and NH-HeH samples were characterized by a hypo-methylation pattern when compared to the remaining samples (Table S4a,b).

The remaining yet unclassified samples were grouped according to recurrent or known BCP-ALL gene fusions: BCL2-IGH (2 patients), *EP300*-*ZNF384* (2 patients) *MEF2D*-*PYGO2* (1 patient), and *KMT2A*-*MLLT3* (1 patient). Six additional samples (3 patients) lacking all of the above-mentioned fusions, had mutations (n = 4) and fusions (n = 2) within *PAX5*. The seven remaining samples (3 patients, and the REL of the classified BCL2-IGH ID) could not be attributed to a specific subtype. Specific lesions could be observed for the different BCP-ALL subgroups: somatic TP53 mutations in LH patients and CRLF2 and other kinase mutations in Ph-like patients (Fig. S2c).

### Clonal evolution reveals volatile and stable mutations driving relapse

**Figure 1:**
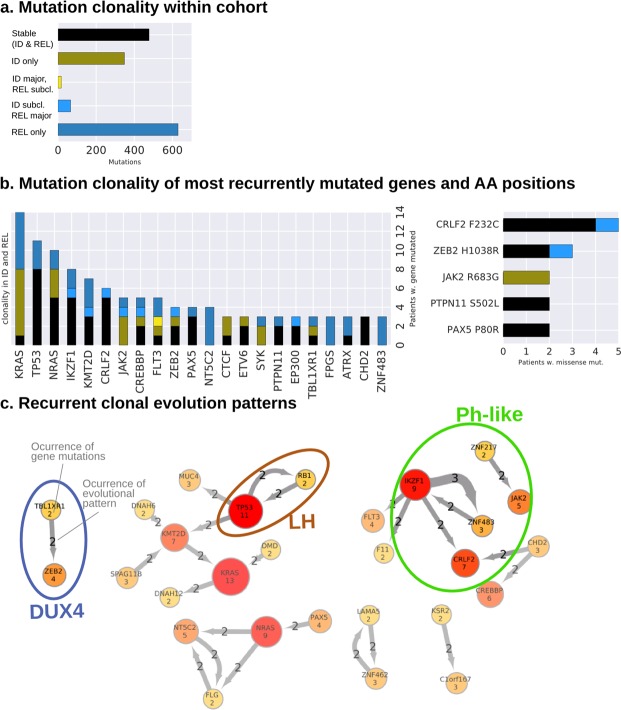
The clonal evolution of BCP-ALL patients reveal volatile and stable gene mutations. (**a**) The left and middle plot shows the mutation counts (y-axis) categorized into different mutational evolution (x-axis). The bar in black shows the stable mutations that were detected in both ID and REL over 10% variant allele frequency (VAF). The green bar shows mutations, which were only detected in ID and disappeared in REL, followed by the yellow bar, which shows mutations that dropped from over 10% VAF in ID to under 10% VAF in REL. The light blue bar shows the amount of mutations, which were increasing VAF from subclonal levels (under 10%), whereas the last bar reflects the mutations what were only detected in REL over 10% VAF. (**b**) The mutation count (y-axis) of the most recurrent genes (x-axis) and their categorization of the clonal evolution, maintaining the color scheme from (**a**). The right-hand plot the same information for recurrent amino acid substitutions by missense mutations with inverted axes. (**c**) The network shows mutations occurring together in the same and/or in a subsequent clone: the observed mutations (amount in gene nodes) are connected by the arrows. The number in the arrow shows how many times the mutations occurred in the same or a descending clone. Relations between mutations associated exclusively with molecular subgroups are highlighted by the ellipses and labels of the defined subgroups.

To dissect patterns of clonal evolution, mutations were categorized into three main categories: *stable* (occurring both at ID and REL; 31% of the mutations); *ID only*, if a mutation was not present in REL (22.6%); *REL only*, if a mutation was acquired between ID and REL (40.9%; Fig. 1a). At relapse, a minority of mutations dropped to subclonal levels (1.1%), meaning under 10% variant allele frequency (VAF), or have expanded from subclonal levels into a major clone (4.3%).

46% in pediatric and 58% in adult (52% overall) of the relapsed leukemia proceeded from a precursor clone characterized by the loss of the major clone, while maintaining a minor clone. 46% in pediatric and 42% in adult (44% overall) of the relapsed leukemia developed from the major clone observed at ID, thus called successor leukemia. Only 7% of the pediatric (4% overall) were classified as novel leukemia, sharing no common mutations between ID and REL (see methods). The different evolution classes distributed evenly across subtypes, pediatric, adults, early and late relapses. One exception was the NH-HeH group with an underrepresentation of the successor (n = 2) and predominance of precursor phenotypes (n = 7) and one novel leukemia (n = 1).

We identified *FLT3*, *JAK2* and *RAS* mutations as ‘volatile’ in the sense that the rise and fall of the clones containing such mutations is notorious (Fig. 1b). Particularly RAS mutations have been reported as subclonal^39^. In our cohort five patients had stable *NRAS* mutations, meanwhile other five *NRAS* mutations were observed either only at ID or REL. Opposed to those ‘volatile’ mutations were *TP53*, *KMT2D*, *CREBBP* and *PAX5* lesions, whose occurrence was either (re-)appearing or even rising into a major clone at relapse (Fig. S4). Recurrent stable amino acid changes were identified in *CRLF2* (n = 5 patients, F232C, all Ph-like) and *PAX5* P80R (n = 2). We observed novel recurring amino acid changes in the cancer-related genes *ZEB2* H1038R (n = 3) and *PTPN11* S502L (n = 2; Fig. 1b; Fig. S4).

To explore clonal hierarchies, we tracked co-occurrence of mutated genes and mutations occurring recurrently in the same or a descendant clone previously calculated for each individual patient (Figs S5–6; Table S5a). We subsequently screened the clonal evolutions for subgroup specificity: Patients with *TBL1XR1* mutations (n = 2; DUX4 subtype) also acquired *ZEB2* mutations in a later clone. The *ZNF483* (n = 3; Ph-like subtype) mutations associated to the Ph-like subtype is novel and only occurred with an *IKZF1* mutation background. The co-occurrence of *RB1* and *TP53* mutations have been exclusively observed in the LH patients (Fig. 1c). While some evolutionary paths of the mutations can be attributed to a genomic subgroup (highlighted in Fig. 1c), the overall pattern remains heterogeneous.

### Novel alterations in chromatin modifiers and metabolic genes define BCP-ALL relapse

**Figure 2:**
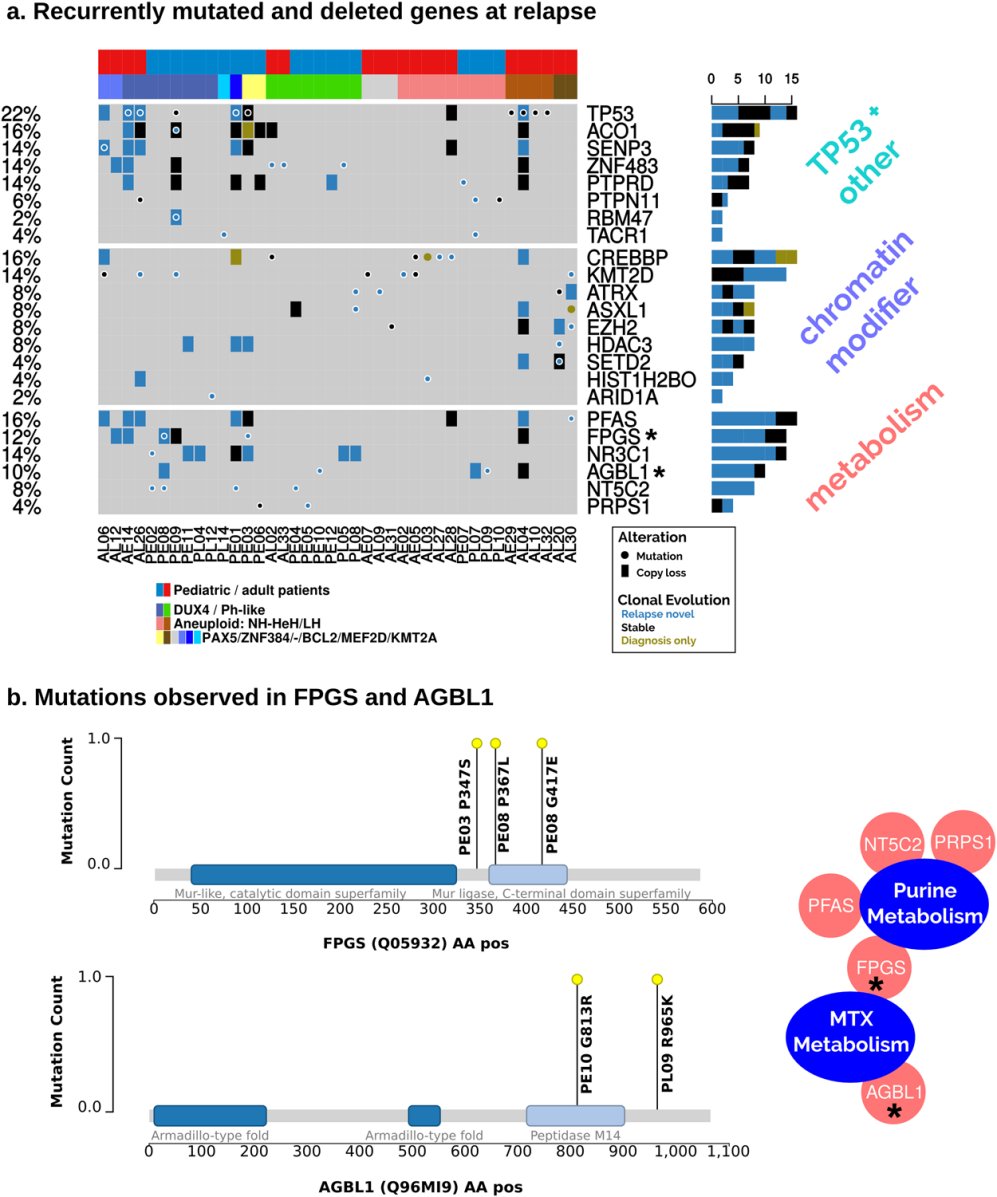
Relapse-specific alteration events of the BCP-ALL patients reveal enrichment of metabolism and chromatin modifier genes. (**a**) Shows relapse-specific mutations and copy number losses. Genes, which had at least one relapse-specific mutation, were selected for their implication in cancer or resistance mechanism. Genes (n = 21) have been classified into three the categories: metabolism, chromatin modifier and TP53 + others. The plot shows that 40 out of 50 (80%) patients have at least one relapse-specific alteration. (**b**) The relapse-specific missense mutation metabolism-related genes FPGS and AGBL1 are shown in the amino acid sequence. Domains have been obtained from InterPro.

At relapse we identified significantly more mutations as compared to ID (adult median mutations per sample: 16 at REL vs 13.5 at ID; pediatric median: 19.5 at REL vs 10 at ID, respectively; Wilcoxon signed-rank test p-values for pediatric and adult samples: 6.37e-05/1.14e-03). Importantly, lesions we identified as preferentially occurring at relapse including non-silent mutations and copy-number losses affected 80% of all patients (n = 40; Fig. 2a and Table S5b). These altered genes could be attributed to functions in epigenetic regulation, metabolism, or are associated with the TP53 pathway. Importantly, protein-protein interactions revealed that the altered metabolism and chromatin-modifying enzymes were tightly intertwined in their functions (Fig. S7).

We identified novel relapse-specific mutations in *FPGS* and *ABGL1*. Both genes showed relapse-specific lesions (n = 4 each) and stable losses (n = 3; Fig. 2a). *FPGS* is involved in the methotrexate metabolism through its function in glutamylation, which has been shown in a non-leukemic context^40^. Both patients have a mutation in or close-by the annotated Mur-ligase domain (IPR036615) related to ATP-binding and ligase activity (Fig. 2b). ATP/GTP Binding Protein Like 1 (*AGBL1)*, a glutamate decarboxylase, is the second gene bearing novel relapse-specific mutations, which is involved in the posttranscriptional glutamylation modifications, similar to FPGS. The observed mutations are located within and immediately downstream of the carboxypeptidase domain (Peptidase M14; IPR000834; Fig. 2b).

### Chemokines, interleukins and NF-kB up-regulation characterize the gene expression signature in DUX4 relapse samples

**Figure 3:**
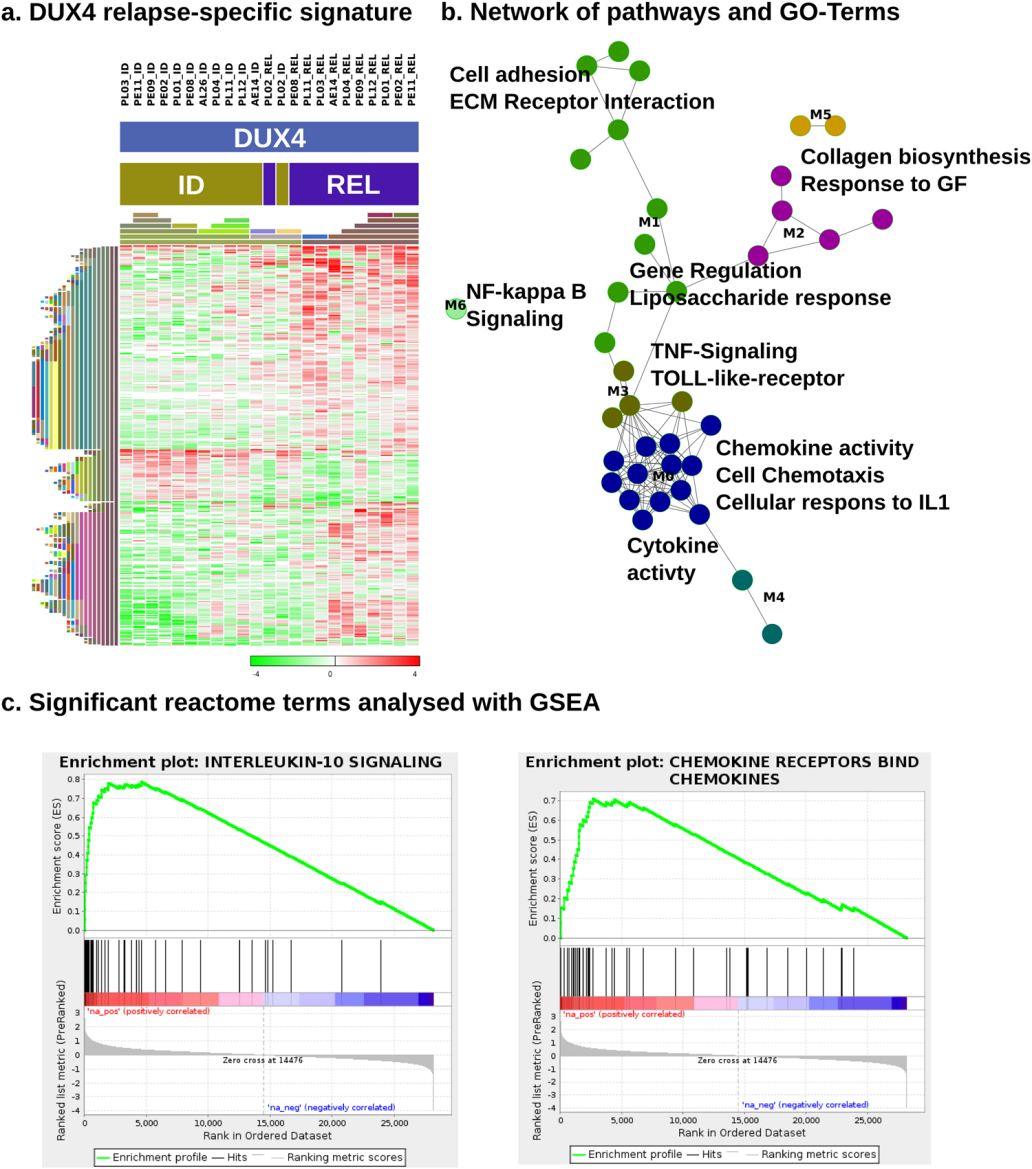
DUX4 relapse-specific gene expression, regulation and pathway analyses reveal a chemokine-driven gene signature. (**a**) Shows the genes involved in the pathways and GO-terms enriched in REL compared to ID (n = 11; ID n = 12) of the DUX4 subtype that are annotated to an enriched pathway in subfigure (**b**). The heatmap is clustered by columns and rows (euclidean median hierarchical clustering), represented by the color-coded bars on the left. The expression data show gene-centered fold changes of log2 TPM. (**b**) Relapse-specific expression signature pathways in the DUX4 subtype. Pathways of interest have been labeled and the edges between the nodes that represent pathways and GO-terms indicate that they share common genes. The FIPlugin for Cytoscape has been used to cluster the network. The resulting clusters are designated by node colors and with labels ranging from M0 to M6. (**c**) GSEA plots for Reactome pathways IL-10 Signaling and Chemokine Receptors. The strong up-regulation of both of these and other pathways corroborate the results in (**a**,**b**).

In addition to mutational alterations acquired at relapsed we investigated also transcriptional changes that contribute to recurrence of the disease. As the molecular subgroups are defined by specific expression signatures, we assayed relapse-specific expression patterns within the three largest subgroups separately. When comparing DUX4 ID to DUX4 REL samples, we observed 1291 (q-value < 0.1; Table S6a) protein coding genes as differentially expressed (Fig. 3a). No significantly deregulated genes were found comparing Ph-like ID to REL or comparing NH-HeH ID to REL samples.

A gene set over-representation of the specific DUX4 relapse-signature revealed that chemotaxis and cytokine interactions were strongly deregulated, many of them over-expressed in relapse compared to the corresponding DUX4 ID samples (Fig. 3b; Table S6b). Amongst the many up-regulated chemokines and interleukins, we found *CCL2*, *CCL20*, *CCL3*, *CCL3L1*, *CCL3L3*, *CCL4*, *CCL4L1*, *CCL4L2*, *CXCL1*, *CXCL2*, *IL1R1*, *IL1R2*, *IL1RN*, *IL3*, *IL4I1*, *IL6 and IL8*. Besides this, TNF-signaling and TOLL-like receptor, NF-kB-signaling and cellular response to interleukin-1 were over-represented terms in DUX4-REL.

A GSEA analysis confirmed multiple interleukin signaling, TOLL-Like and chemokine receptor gene sets as up-regulated (Fig. 3c; Table S6c). The chemokine ligand *CCL2*, Interleukin 8 *(IL8)* and the *PDFGB* gene were all amongst the top 30 up-regulated genes. *CCL2* and *IL8* are established targets of the NF-kB pathway. Regarding NF-kB-signaling, we observed that the genes *NFKBIA*, *NFKB1*, *NFKB2 and NFKBID* were all up-regulated at DUX4 REL and the GSEA results delivered various up-regulated NF-kB Reactome pathways in DUX4-REL. *CAMK2A*, a gene related to NF-kB-activation and has been demonstrated to act anti-apoptotic in cancer stem-like cells, is the third-most up-regulated gene at DUX4-REL^41^.

### Proteomics identifies up-regulation of metabolic pathways at relapse

**Figure 4:**
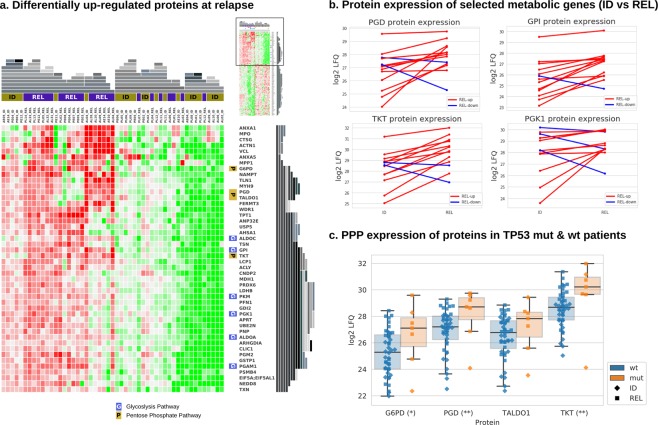
Proteomics of BCP-ALL relapse. (**a**) The heatmap shows the proteins that are significantly up-regulated at REL. Proteins of the Phosphate Pentose pathway (PPP) and Glycolysis pathway, both overrepresented pathways at REL, are indicated at the right side of the heatmap by a P or G, respectively. The heatmap has been hierarchically clustered (top and right side representations in grey) using Gitools^58^. (**b**) Shows expression change of four metabolic proteins at ID and REL. If the net increase in expression is positive, the line is shown in red, otherwise in blue. (**c**) The box plots show protein expression levels of the members of the PPP as presented in the heatmap in (**a**), itemized by TP53 wild type (wt; n = 44) and TP53 sequence mutations (mut; n = 7). The significances are represented by the stars next to the protein name as follows: 1 star (*): 0.05 ≥ p > 0.01, 2 stars (**): 0.01 ≥ p > 0.001.

With this study, we introduce the first comprehensive MASS-Spec proteome data set for relapsed BCP-ALL. We successfully quantified the expression of 1460 proteins across the 51 ID and REL samples from 36 patients, which had sufficient protein quality and quantity yielding high quality read-outs (Table S7a,b). De-regulated proteins at REL identified enrichment of proteins specifically involved in metabolic pathways including Glycolysis, Gluconeogenesis and the Pentose Phosphate Pathway (PPP; Fig. 4a; Table 2). The proteins GPI (Glycolysis), PGD and TKT (PPP) were concordantly up-regulated at relapse on protein abundance as well as mRNA transcript levels in over 50% of the samples (Figs 4b and S8). These two pathways, including the proteins G6PD, PGK1, GPI, PGD, and TKT, were particularly up-regulated in Ph-like relapse samples (Fig. S9).

**Table 2:** Top 10 canonical Ingenuity pathways for the differentially expressed proteins at BCP-ALL relapse.

Gene	P-value	Molecules (Proteins)
Glycolysis I	0.000227	ALDOA, ALDOC, GPI, PGAM1, PGK1, PKM
Gluconeogenesis I	0.000227	ALDOA, ALDOC, GPI, MDH1, PGAM1, PGK1
Pentose Phosphate Pathway	0.001333	G6PD, PGD, TALDO1, TKT
CDP-diacylglycerol Biosynthesis I	0.005917	AGPAT5, CDS2, LPCAT3, MBOAT7
Phosphatidylglycerol Biosynthesis II (Non-plastidic)	0.006806	AGPAT5, CDS2, LPCAT3, MBOAT7
EIF2 Signaling	0.009853	RPL14, RPL17, RPL23A, RPL3, RPL36, RPL7, RPL8, RPS17
Sirtuin Signaling Pathway	0.010153	ACLY, G6PD, H1FX, LDHB, NAMPT, PCK2, PGAM1, PGK1, TIMM9
Adenine and Adenosine Salvage I	0.010567	APRT, PNP
Antigen Presentation Pathway	0.013300	HLA-DRA, HLA-DRB1, TAP2, TAPBP
Pentose Phosphate Pathway (Oxidative Branch)	0.039164	G6PD, PGD

Correlating specific genetic lesions with differential protein expression at relapse, we detected 310 proteins significantly altered in TP53 mutated cases. We found that several PPP proteins described above to be up-regulated at relapse, show a significant up-regulation in TP53 mutated compared to TP53 wild type cases, linking TP53 mutations with activation of the Glycolysis and PPP at relapse. PGD and TKT were highly expressed in TP53 mutated samples (p-value < 0.01), while G6PD (p = 0.12) and TALDO1 (p = 0.07) display clear tendencies (Fig. 4c).

## Discussion

Relapse remains a major source of morbidity and mortality in BCP-ALL patients. To characterize the genomic basis and functional context of BCP-ALL relapse, we applied a multi-omics approach on matched diagnosis and relapse samples from pediatric and adult patients (n = 50) lacking well-studied recurrent cytogenetically detectable chromosomal rearrangements *BCR-ABL1*, *KMT2A-AFF1*, *ETV6-RUNX1*, *TCF3-PBX1*.

The samples were allocated into recently described subgroups as defined by drivers including fusion genes or ploidy patterns revealing specific gene expression and DNA methylation profiles. Said patterns were stable in all patients between ID and REL despite loss and acquisition of genetic lesions over extended time periods (maximum time to relapse: 8.6 years) and under the selective pressure of intensive chemotherapy. This supports the concept of driver alterations as founding lesions, which define a constant subgroup-specific biological framework for the individual disease^42^.

Analyzing acquired relapse-specific genomic alterations, we observed a high frequency of genes related to nucleotide metabolism, which has previously shown to be a key mechanism for resistance in leukemia. E.g. *NT5C2*^16^ and *PRPS1*^25^ have shown to promote resistance to purine analogue treatments, both also mutated within our cohort. We identified novel missense mutations p.G417E, p.P367L and p.P347S in two pediatric patients in the gene *FPGS*, which has been implicated in the resistance to methotrexate^40^. The majority of these alterations were either acquired at relapse or developed from a subclone, supporting a role in drug resistance mechanisms. In addition, we reveal novel relapse-acquired alterations in four patients were identified in the ATP/GTP-binding protein-like 1 gene *AGBL1*, including the two missense mutations p.G813R and p.R965K in two pediatric patients. *AGBL1* is a member of the cytosolic carboxypeptidase family, which catalyze the decarboxylation of polyglutamylated proteins^43^. Polyglutamylation is essential for the methotrexate activation and intracellular retention. Thus, alterations in *AGBL1* might affect the activity of methotrexate. Mutations on both proteins (FPGS and AGBL1) are within annotated functional domains (Mur-ligase and hydropeptidase) and the immediate neighborhood, but the absence of protein structures limits the interpretation of the exact mode of action. While the protocols of treatment were very similar for both pediatric and adult patients, *FPGS* and *AGBL1*, as well as mutations in genes with critical functions in response to purine analogues (*NT5C2* and *PRPS1*) or steroids (*NR3C1*) occurred exclusively in pediatric relapse patients. Adult patients on the other hand showed a higher likeliness to accumulate more mutations in chromatin remodeling factors (e.g. *CREBBP*, *KMT2D*, *ATRX*, *SETD2;* Fig. 2).

In addition to metabolism-related mutations, chromatin-remodeling factors were frequently altered at relapse^44^. Acquired alterations included the transcriptional coactivator *CREBBP*, involved in glucocorticoid responsiveness^23^, and the histone methyl-transferases *KMT2D*, *EZH2*, and *SETD2*. We show that the chromatin-remodeling factors and the metabolic proteins are tightly connected when mapped on a protein-protein interaction network^44^ (Fig. S7b). Together, alterations in both nucleotide metabolism genes and epigenetic regulators may cooperate in escape mechanisms, fostering chemo-resistant relapse.

So far, clonal plasticity and evolutional changes have been nearly exclusively studied on the genomic level. Here we show that relapse-specific changes are not restricted to the highlighted genetic alterations, but transcriptional deregulation on the mRNA as well as protein level contribute to leukemic evolution without losing their BCP-ALL subgroup-specific identity. We defined a novel relapse-associated gene expression signature for DUX4 leukemias. While DUX4 is not expressed in normal B-cells, the DUX4-translocation has been shown to impair its normal DNA-binding in leukemic cells^45^, which in our cohort results in deregulated pathways including NF-kB-signaling, chemokine/chemotaxis. Chemokines and interleukins were markedly up-regulated in the DUX4 relapse as our pathway and gene set enrichment analyses (GSEAs) showed. These results hint to an involvement of the microenvironment contributing to leukemic relapse in DUX4 patients, particularly as the chemokine signature play a central role in relapsed leukemia^46–48^. The cytokines with the most prominent relapse-specific upregulation in DUX4 are all synthesized by monocytes. Analysis of corresponding surface marker mRNA expression revealed that DUX4 samples acquired CD14 expression at relapse in 7 of 11 cases (>2.5-fold increase from ID expression level) while the remaining 4 cases retained a stable CD14 expression from ID to relapse (<1.5-fold change). In addition monocytic differentiation factors CEBPA and CEBPB were upregulated in DUX4 relapse samples (data not shown). These data provide evidence that DUX4 ALL represent a switchALL phenotype and that relapse in DUX4 ALL occurs with monocytic differentiation, as previously shown for a small proportion of BCP-ALL^49^.

In addition, results from myoblasts cells have shown that expression of DUX4 leads to overexpression of chemotaxis-related genes and to a greater migration potential by CXCR4^50^. CXCR4 and chemotaxis genes in DUX4-translocated leukemia may contribute and enhance leukemia-stroma interactions facilitating DUX4 blasts to survive treatment. Additionally, *CAMK2A*, the third most up-regulated gene at DUX4 relapse, acts as an anti-apoptosis regulator by activating NF-kB in metabolic stress-resistant cancer stem cells^41^. The prominent up-regulation the NF-kB pathway targets *CCL2* and *IL8* further hint at a crucial involvement of the pathway.

The analysis matched proteomics and genomics data for BCP-ALL is yet unexplored territory^51^ as the small size of the B-cell precursor blasts and the material- and time-intensive nature of MASS-SPEC render the acquisition of homogeneous BCP-ALL proteome challenging. We here provide novel insights into the proteomic consequences by dissecting the relapse-specific proteomics data set. We unraveled that proteins involved in glycolysis (GPI, PGK1, PGAM1) and PPP (G6PD, PGD, TKT, TALDO1) are specifically up-regulated at relapse, particularly in non-DUX4 samples. Multiple adaptations have been identified in malignant cells for increased energy demands and oxidative stress imposed by oncogenic transformation^52^. In BCP-ALL, frequently deleted B cell transcription factors (IKZF1, PAX5) have been characterized as gatekeepers toward malignant transformation through limiting the energy supply below a minimum required threshold^53^. Moreover, BCP-ALL cells critically depend on the PPP to salvage oxidative stress^27^. Given that oxidative stress is a major effector of chemotherapy, it seems likely that our observation of an increased PPP at BCP-ALL relapse represents an adaptation and a resistance mechanism to first line chemotherapy. Beyond its function in energy supply, glycolysis has a major role in providing metabolic intermediates for the biosynthesis of cellular components required during proliferation^54^. Thus, an increase in glycolysis in relapsed BCP-ALL possibly reflects an adaptation to the increased demand for cellular building blocks for the rapid expansion of chemoresistant relapse clones. Interestingly, in our cohort the up-regulation of PPP proteins was not only linked to relapse but also to the presence of TP53 mutations. Given that mutant TP53 can also exert gain-of-functions^55^, our observation strengthens clinical evidence of preclinical models showing that TP53 promotes PPP through transcriptional activation of its target gene TIGAR^27,56^.

In addition to the dissection of relapse-specificity identified at different levels (DNA, RNA, protein changes), our study provides a comprehensive analysis of pediatric and adult patients (Table S8). Data acquisition was performed using the same platforms and analysis pipelines, allowing for direct comparison with all patients were treated based on a pediatric-inspired protocols. With this approach, we show that in adults unfavorable features were enriched, such as lower frequency of DUX4 fusions and a tendency towards a higher TP53 mutations frequency. Additionally we show that the relapse-specific mutations reveal a chromatin modifier profile in adults whereas a metabolic profile was prominent in relapsed pediatric patients.

Moreover, we explored molecular differences between early or late relapses and identified enrichment of CDKN2A/B deletions as well as novel alterations (predominantly deletions) in the gene protein tyrosine phosphatases receptor type D (*PTPRD*), specifically in early relapse (7/25 early vs. 0/25 late relapse). The co-deletion of *PTPRD*, a candidate tumor suppressor, and *CDKN2A* has been reported to accelerate tumorigenesis, facilitating an early escape^57^.

## Conclusion

We conclude that alterations in metabolic pathways are one major hallmarks of relapsed leukemia as (a) metabolic genes were frequently mutated on the genomic level and confer resistance to chemotherapeutic agents and (b) our proteomic signature defines glycolysis and PPP as key deregulated pathways in relapsed BCP-ALL. We also provide novel insights into clonal evolution, age and relapse-specific molecular alterations and show that the molecular BCP-ALL subtype eclipses age and treatment. The pediatric-dominated DUX4 relapse-specific signature is characterized by chemokine interaction, interleukin signaling and NF-kB pathway extends also to the adult. We think that the presented results contribute to the better understanding of relapsed ALL in adult and pediatric patients facilitating tailored therapeutic concepts.

## Supplementary information

Supplementary Material and Figures

Supplementary Dataset 1-10

## Data Availability

The analysed and primary datasets supporting the conclusions of this article are available in the and at the European Genome-phenome Archive repository under the accession number EGAS00001002856.
