# Supplementary material for: Integrated analysis of relapsed B-cell precursor Acute Lymphoblastic Leukemia identifies subtype-specific cytokine and metabolic signatures
Source: Sci Rep. 2019 Mar 12;9:4188. doi: 10.1038/s41598-019-40786-1 (PMC6414622; doi:10.1038/s41598-019-40786-1)
Supplement: Supplementary file 1 — Supplementary Material and Figures [file 41598_2019_40786_MOESM1_ESM.pdf]

Supplementary Material & Methods: Integrated analysis of relapsed B-cell precursor Acute Lymphoblastic Leukemia identifies subtype-specific cytokine and metabolic signatures

Michael P Schroeder[1], Lorenz Bastian[1,2,3], Cornelia Eckert[2,3,4], Nicola Gökbuget[2,3,5], Alva Rani James[1,2,3], Jutta Ortiz Tanchez[1], Cornelia Schlee[1], Konstandina Isaakidis[1], Björn Häupl[2,3,5], Katharina Baum[6], Oscar Arturo Migueles Lozano[6], Khouloud Kouidri[5], Kuan-Ting Pan[7], Henning Urlaub[7,8], Stefan Schwartz[1], Thomas Burmeister[1], Arend von Stackelberg[2,3,4], Dieter Hoelzer[5], Heike Pfeiffer[5], Michael A Rieger[2,3,5], Stefanie Göllner[9], Thomas Oellerich[2,3,5] , Martin Horstman[10], Martin Schrappe[11], Jana Wolf[6], Renate Kirschner-Schwabe[2,3,4], Monika Brüggemann[12], Carsten Müller-Tidow[9], Hubert Serve[2,3,5], Martin Neumann[1,2,3], Claudia D Baldus[1,2,3,12]

1. Charité, University Hospital Berlin, Campus Benjamin Franklin, Department of Hematology and Oncology, Berlin, Germany
2. German Cancer Research Center (DKFZ), Heidelberg, Germany
3. German Cancer Consortium (DKTK), Heidelberg, Germany
4. Charité, University Hospital Berlin, Pediatric Hematology/Oncology, Campus Rudolf Virchow, Berlin, Germany
5. Goethe University Hospital, Department of Medicine II, Hematology/Oncology, Frankfurt/M., Germany
6. Max Delbrück Center for Molecular Medicine in the Helmholtz Association, Berlin, Germany
7. Max Planck Institute for Biophysical Chemistry, Göttingen, Germany
8. University Medical Center, Göttingen, Germany
9. University Clinic Heidelberg, Department of Hematology, Oncology & Rheumatology, Heidelberg, Germany
10. Research Institute Children's Cancer Center, Dept. of Pediatric Hematology and Oncology, University Medical Center Hamburg, Hamburg, Germany
11. University Hospital Schleswig-Holstein, Campus Kiel, Department of Pediatrics, Kiel, Germany.
12. University Hospital Schleswig-Holstein, Campus Kiel, Department of Hematology and Oncology, Kiel, Germany

Supplementary Material & Methods

Patients samples selection requirements. The sample triplets retrieved at initial diagnosis (ID), complete remission (CR) and relapse (REL) with the requirement of a minimal residual disease level at CR below 0.01. As the clinical protocol for adult and pediatric patients relapse time are different, the samples have been selected to evenly distribute into the categories of early (ER; time of REL < 700 days) and late relapse (LR; time of REL => 700 days) in order to have comparable clinical settings (Table S2a-b).

Alignments. Whole-Exome-Seq and Panel-Seq data have been aligned with bwa to the human genome build GRCh37.75[28] using the bcbio-nextgen pipeline v0.9.1a-7da8dce (https://github.com/chapmanb/bcbio-nextgen) employing a series of tools (Table S9). RNA-sequencing data were aligned with STAR-aligner (2-pass mode)[29] to the same genome build and with the transcript annotation from Ensembl GRCh37 distributed by bcbio-nextgen. Samples failing quality control performed with RNA-SeQC[59] were not used for expression analyses.

Mutation detection. ID and REL patient samples were whole-exome sequenced and compared to the matched CR sample to detect somatic mutations in ID and REL. The bcbio-nextgen pipeline was used to employ Mutect, Freebayes, Vardict, Varscan as mutation callers. Mutations (nucleotide polymorphisms) with the following criteria have been accepted: two out of four tools reported the mutation, variant allele frequency of 10% in either ID or REL, at least 10 reads at the genomic position were aligned, at least 3 reads supporting the mutation and variant allele frequency at CR must be under 5% (estimated maximum of minimal residual disease). Mutations were annotated with GEMINI (within bcbio-nextgen). For the 3 (AE07, AE10 and AL31) patients that underwent allogeneic transplant and no matching sample of the transplant donor could be retrieved, the calling relapse mutations was limited to mutations observed at ID.

Mutation validation via panel-sequencing. We used customized biotinylated RNA oligo pools (SureSelect, Agilent, Santa Clara, California) to hybridize the target regions comprising 362 kbp. The panel was designed to cover all single mutations. Genes that were mutated recurrently according to our mutation detection have been re-sequenced entirely. For all single mutations we designed probes specifically for the variant position. We sequenced the libraries in pools of 16 to 20 samples on a HiSeq2000 (100bp paired-end reads). We obtained an average coverage of 30.1 Mio mapped reads/sample. We screened the called exon-based mutations in the panel-sequencing data with the same (10%) alternate read frequency thresholds has been applied after correcting for cellular contents in order to obtain the final mutation set. The cellular contents were measured at sample collection using flow cytometry and/or MRD. It was additionally verified and corrected if necessary through the analysis of heterogeneous polymorphisms observed at CR (1).

Recurrent gene mutations and amino acid substitutions. The data of Figure 1 and Figure S1 show the most recurrent mutations and amino acid substitutions filtered by the cancer gene list (see below). The genes *MUC4*, *TTN*, *NBPF1*, *MUC12* have been blacklisted for all recurrence analyses due to their known high passenger mutation rate.

Copy number variation. The aligned WES sample trios have been used to employ copywriteR to calculate the log-ratio counts in bins of 20-50kb (shortest possible to obtain a MAD score

around 0.25) between ID or REL and CR. With CNVkit[30], we called the copy number data using the log-ratio counts from copywriteR and single nucleotide variant (SNV) frequencies of SNVs being heterozygous in the matched CR sample and annotated in the EXAC database (all called segments are in 3c). CNA statistics have been calculated without samples that were too noisy or have received an allo-transplant (AE07_REL, AE10_REL, AL18_ID, AL18_REL, AL31_REL, PE07_ID, PE07_REL). Before proceeding to clonality analysis and chromosomal stats analysis, we corrected the b-allele frequency for sample purity.

Cancer gene prioritization. In order to incorporate prior knowledge about genes involved in cancer in various entities, we have created a cancer gene list based on multiple databases (IntOGen[60], Tumorportal[61], Cancer Gene Census[62], GMALL panel (manually curated list of genes known to be frequently altered in acute leukemia; Table S10)

Clonality analysis. The variant allele frequencies (VAF), estimated blast contents and the called copy number distributions were used to perform a clonality analysis of each ID and REL sample pair. Pyclone[32] was used to estimate the clonal composition of each sample and Schism[33] was used to reconstruct the clonality tree for all samples (Figure S5). To incorporate all possible CNA calls, all sample data have been considered and CNAs have been manually double-checked for putative two-hit events for problematic samples (see copy number analysis). Cellular prevalences of the cancer-associated genes can be found in Table S5 accompanied by the classification of the mutations into activating and loss of function, which have been obtained using OncodriveROLE[63] via the Cancer Genome Interpreter[64] platform can be found in. MRD markers were confirmed to persist for samples with a seemingly novel mutational phenotype.

Expression estimation and differential expression (DE). The aligned RNA-seq data have been used to employ Stringtie[67] to estimate both annotated and possible novel transcripts where TPM (transcripts per million) and FPKM values were extracted. FPKM values were used with R Bioconductor Linear Models for Microarray (LIMMA) Voom package for DE analysis as LIMMA leverages the sample dependency in our dataset with its duplicate correlation feature. Additionally, we included the diagnosis and relapse time factors into the design (makeContrasts) to avoid the inflation of the variance due to time factor for each subtype. Finally, we filtered limma results by p-value <= 0.01 and abs(fold change) >= 1.5. For the DUX4-relapse ID versus REL comparison, the output of LIMMA Voom[65] the mis-regulated genes have been filtered at q-value < 0.1 and abs(fold change) >= 1.5. **DUX4 RELvsID DE pathway enrichment analyses** were performed using the DAVID online resource[66] (Table S6a).

GSEA DUX4 relapse analysis was performed with GSEA java-application v3.0[67] in pre-ranked list mode for the Reactome pathways performing 1000 permutations and considering gene sets between 15 and 500 genes. As input we used all genes ranked by log-fold change DUX4 REL versus ID obtained by from the limma voom analysis.

Methylation data analysis. The ID and REL samples have been assayed with the Illumina 450k methylation array. All values have been normalized using the SWAN algorithm. The 3rd quartile of all CpGs standard variation served as cutoff for differential methylation analyses. In order to detect differentially methylated regions we used the bumphunter[38] algorithm using the most variant quartile of CpG probes, which searches for differentially methylated regions in an annotation-unbiased manner. Separate bumphunter runs have been performed for ID and REL for

the subtypes DUX4, NH-HeH and Ph-like using all subtype samples versus the rest of the cohort. Additionally bumphunter runs have been performed for ID vs REL samples for the subtypes DUX4, NH-HeH and Ph-like. The output regions have been filtered by p-value < 0.05 and p-value Area < 0.05. The ensembl gene annotations and variation build has been used to annotated close-by genes and regulatory elements. Genes annotated as protein coding and overlapping with the differentially methylated regions (DMR) or located at a maximum distance of 5000 bp upstream or 1000 bp downstream were considered for further analysis. Regulatory features were allowed to be up to 100 bp away from the DMR, except for the CTCF sites where overlap was required. We also performed a region-biased promoter methylation analysis using ID versus REL samples for the whole cohort and for the three main subtypes separately.

Differentially expressed and methylated (DEM) genes analysis. For each subtype, we retrieved the filtered results from the bumphunter runs for methylation data and the filtered results from the limma voom runs for RNAseq data. We obtained the intersection, denominated thereafter as DEM genes. The genes were afterwards classified according to the two known methylation regulation mechanisms: The 'Meth silencing' classification for the genes with a decreased expression accompanied by an increased methylation status and the 'Meth facilitating' classification for the genes with an increased expression accompanied by a decreased methylation status. The DEM genes overlap within the top 1000 of both differentially expressed and differentially methylated analyses have been tested with Fishers's exact test (see 4a). Genes with hypermethylation co-occuring with under-expression were classified as meth silencing and whereas genes with occurrence of hypomethylation and over-expression were classified as meth facilitating (4b).

Fusion detection. All reported gene fusions have been detected with FusionCatcher v0.99.7c beta (https://github.com/ndaniel/fusioncatcher)[34] or deFuse v0.7.0 (https://bitbucket.org/dranew/defuse)[35] with default settings. The required fusion evidence has been set to a minimum of two supporting spanning reads for each fusion in each sample. In a first step, we identified fusions with high read count and reported in BCP-ALL subgroups and in a second step novel fusions which are enriched in the molecularly defined BCP-ALL subgroups.

DUX4 classifier. We used the TPM expression values for the 82 samples passing quality control and divided them into DUX4 (n=23) and other (n=59). We then filtered the expression table for DUX4 protein coding DE genes (FDR < 0.1) and added 7 house-keeping genes (*ACTB, GAPDH, RPLP1, LDHA, NONO, PGK1, PPIH*). Thereafter, we kept the genes having expression values in min. 30% of the samples and empty values were filled with the smallest available value in the matrix of the matrix divided by 2. For each non-housekeeping gene, we created an 8-gene submatrix with the gene itself and the 7 housekeeping genes. This submatrix was used for training 20 random forest machine learning models, each using 80% of the samples (selected randomly, but stratified by class). The remaining 20% of the samples were then predicted two times: once using the unmodified submatrix samples (predA) and once proceeding a shuffling of non-housekeeping gene (predB). The two predictions were assessed with the f1-score and the accuracy decrease due to value shuffling was obtained by accuarcy(predA) - accuracy(predB). The mean decrease accuracy was obtained from the 20 iterations (MDEC score). Genes were ranked by maximum MDEC score and filtered for positive fold change between DUX4 sample expression and the remaining samples. The top 15 genes were used to validate the classifier in

an external BCP-ALL RNA-Seq cohort using hierarchical clustering.

Sample preparation for proteomic analysis. Patient samples were resuspended in lysis buffer containing 0.2 % DCA and 50 mM TCEP followed by incubation at 90 °C for 1 hour. For filter-aided sample preparation (FASP)[68] lysates were mixed with 200 µl of exchange buffer (8 M urea, 0.2 % DCA, 100 mM ammonium bicarbonate) in spin column filter units and centrifuged at 14,000 g for 10 minutes. After repeating this step twice and discarding the eluates, 100µl of alkylation solution (8M urea, 50mM iodoacetamide, 100mM ammonium bicarbonate) were added and the samples were mixed at 300 rpm for 1 hour at 37 °C protected from light. Following centrifugation at 14,000g for 10min and addition of exchange buffer (200µl) the samples were washed three times with 100µl of digestion buffer containing 0.2% DCA and 50mM ammonium bicarbonate. For protein digestion, trypsin was added to the filter units at an enzyme/substrate ratio of 1:100 (w/w) and the samples were incubated over night at 37°C. Proteolytic peptides were collected by centrifugation at 14,000g for 10min followed by two washes with 50µl of peptide recovery solution (50mM ammonium bicarbonate). Next, 200µl of ethyl acetate were added to the peptide-containing filtrate, which resulted in the formation of an organic upper layer and an aqueous bottom layer. The aqueous layer was mixed with 2.5µl of TFA and the samples were sonicated for 10 seconds. After centrifugation of the filtrates at 16,000g for 10min, the upper organic layer was discarded and the last two steps were repeated twice. Afterwards, the samples were incubated at 60 °C for 15 min to allow for evaporation of ethyl acetate. In order to remove residual solvent and volatile salts the samples were dried in a vacuum concentrator three times after adding 50 µl of 50 % methanol.

Mass-spectrometric analysis. Peptides were resuspended in sample loading buffer (2% acetonitrile and 0.05% trifluoroacetic acid) and separated on an UltiMate 3000 RSLCnano HPLC system (Thermo Fisher Scientific) coupled online to a Q Exactive Plus mass spectrometer (Thermo Fisher Scientific). First, the peptides were desalted on a reverse-phase C18 pre-column (Dionex 5 mm long, 0.3 mm inner diameter) for 3 minutes. After 3 minutes the pre column was switched online with the analytical column (30 cm long, 75 µm inner diameter) packed in-house with ReproSil-Pur C18 AQ 1.9 µm reversed-phase resin (Dr. Maisch GmbH). Solvent A consisted of 0.1% formic acid in water, and solvent B consisted of 80% acetonitrile and 0.1% formic acid in water. The peptides were eluted from the column with solvent B (5% to 46% gradient) at a flow rate of 300nL/min over 106 min. The temperature of the pre-column and the column was set to 60°C. MS data were acquired on the Q Exactive Plus instrument in data-dependent Top20 mode, where the most intense 20 precursors within the m/z range of 350–1600 Th were selected from a survey MS1 scan for MS2 fragmentation with an isolation window of 1.6 Th and dynamic exclusion of 25 seconds. Selected precursors underwent HCD fragmentation with normalized collision energy of 28. MS1 and MS2 scans were acquired at a resolution of 70,000 or 17,500 with an Automatic Gain Control (AGC) target of 1E6 or 1E5, respectively. The maximum ion injection (IT) time of an MS2 scan was set to 50 ms.

MS data processing. Raw data from LC-MS/MS measurements were analyzed using the MaxQuant software[69]. MS/MS spectra were searched with the Andromeda engine[70] against the UniProtKB/Swiss-Prot human database containing 20,170 protein entries (download date: April 2017) and an integrated database containing frequently observed contaminants (245 entries). Precursor and fragment-ion mass tolerances were set to 6 and 20 ppm, respectively, after initial

recalibration. Protein N-terminal acetylation and methionine oxidation were allowed as variable modifications. Carbamidomethylation of cysteine was defined as a fixed modification. Minimum peptide length was set to seven amino acids, with a maximum of two missed cleavages. The false discovery rate (FDR) was set to 1% at both the peptide and the protein level by using a forward-and-reverse concatenated decoy database approach. Label-free quantification was performed with the “match between run” option enabled[71]. Matching time window and alignment time window were set to 1 and 20 min, respectively.

In order to maintain high analysis quality, stringent filtering criteria were applied to data derived from patient sample preparations. First, samples with Pearson correlations between technical replicates less than 0.9 were removed. Next, Proteomics Quality Control (PTXQC)[72] was applied in order to assess the overall qualities of the MaxQuant-processed results from the remaining patient samples. Samples with QC scores less than 0.6 or with protein hits less than 1200 were removed from the following analysis. Proteomic changes in the filtered samples were analyzed using the Perseus software[73]. Decoy proteins, potential contaminants and protein groups that were quantified in less than 75% of all patient samples were removed. Afterwards, missing values were imputed with default settings. LFQ intensity differences between sample groups representing protein expression profiles at initial diagnosis (ID) and relapse (REL) were statistically evaluated. For this, the log-transformed LFQ intensity values were compared using a Student’s t-test with a significance threshold of $p<0.05$ and the respective LFQ intensity ratio thresholds were deduced by performing outlier analyses ($p<0.05$). In order to extract affected biological pathways, significantly regulated proteins were further subjected to Ingenuity pathway analysis[74].

Sample classification of the main groups. The samples have been classified into subtypes. All samples having DUX4-fusions have been classified as a subtype. DUX4 samples without RNA-seq data have been classified via the methylation data clustering. Ph-like samples have been classified via the published gene signature and CRLF2-, JAK2- and SH2B3-fusions. Two additional samples without RNA-seq data were classified as Ph-like based on their matched sample having a Ph-like expression, harboring CRLF2 mutations and clustering with the other Ph-like samples in the methylation PCA analysis. Aneuploid samples were defined as samples having 3 or more whole-chromosomal aneuploidies. This group was further classified into low-hypodiploid (LH), near-haploid (NH) and high hyperdiploid (HeH). The NH and HeH samples share a common expression signature and were therefore considered as one group for the study. We used reported differentially expressed genes[75] to confirm the NH-HeH gene expression pattern, which is shared amongst our masked NH-HeH and high hyper-diploid samples (data not shown). All these assignments were exclusive to each other.

Sample classification in-silico cross-validation of the main groups. For the four subgroups with most samples (Ph-like, DUX4, NH-HeH and low-hypodiploid), we performed an in-silico cross-validation of group assignment. We compared the amount of differentially methylated CpGs and differentially expressed genes of the assigned groups to randomly chosen sample groups of equal size. The assumption is that a biological meaningful sample group will have a stronger signature and thus reveal a greater amount of significantly different (one-way anova p-value < 0.01) entities when compared with the remaining cohort. This comparison was repeated for a 100 times in order to obtain an empirical p-value. For the gene expression data, we used all protein

coding genes for the comparison (Figure S7a). For the methylation data, we used 10'000 randomly chosen CpGs for each iteration (Figure S7b).

Supplementary References

59. DeLuca, D. S. *et al.* RNA-SeQC: RNA-seq metrics for quality control and process optimization. *Bioinforma. Oxf. Engl.* **28**, 1530–1532 (2012).
60. Gonzalez-Perez, A. *et al.* IntOGen-mutations identifies cancer drivers across tumor types. *Nat. Methods* **10**, 1081–1082 (2013).
61. Lawrence, M. S. *et al.* Discovery and saturation analysis of cancer genes across 21 tumour types. *Nature* **505**, 495–501 (2014).
62. Futreal, P. A. *et al.* A census of human cancer genes. *Nat. Rev. Cancer* **4**, 177–183 (2004).
63. Schroeder, M. P., Rubio-Perez, C., Tamborero, D., Gonzalez-Perez, A. & Lopez-Bigas, N. OncodriveROLE classifies cancer driver genes in Loss of Function and Activating mode of action. *Bioinformatics* **30**, (2014).
64. Tamborero, D. *et al.* Cancer Genome Interpreter annotates the biological and clinical relevance of tumor alterations. *Genome Med.* **10**, 25 (2018).
65. Law, C. W., Chen, Y., Shi, W. & Smyth, G. K. voom: precision weights unlock linear model analysis tools for RNA-seq read counts. *Genome Biol.* **15**, R29 (2014).
66. Huang, D. W., Sherman, B. T. & Lempicki, R. A. Systematic and integrative analysis of large gene lists using DAVID bioinformatics resources. *Nat. Protoc.* **4**, 44–57 (2008).
67. Subramanian, A., Kuehn, H., Gould, J., Tamayo, P. & Mesirov, J. P. GSEA-P: a desktop application for Gene Set Enrichment Analysis. *Bioinforma. Oxf. Engl.* **23**, 3251–3253 (2007).

68. Wiśniewski, J. R., Zougman, A., Nagaraj, N. & Mann, M. Universal sample preparation method for proteome analysis. *Nat. Methods* **6**, 359–362 (2009).
69. Cox, J. & Mann, M. MaxQuant enables high peptide identification rates, individualized p.p.b.-range mass accuracies and proteome-wide protein quantification. *Nat. Biotechnol.* **26**, 1367–1372 (2008).
70. Cox, J. *et al.* Andromeda: a peptide search engine integrated into the MaxQuant environment. *J. Proteome Res.* **10**, 1794–1805 (2011).
71. Cox, J. *et al.* MaxLFQ allows accurate proteome-wide label-free quantification by delayed normalization and maximal peptide ratio extraction. *Mol. Cell. Proteomics* mcp.M113.031591 (2014). doi:10.1074/mcp.M113.031591
72. Bielow, C., Mastrobuoni, G. & Kempa, S. Proteomics Quality Control: Quality Control Software for MaxQuant Results. *J. Proteome Res.* **15**, 777–787 (2016).
73. Tyanova, S. *et al.* The Perseus computational platform for comprehensive analysis of (prote)omics data. *Nat. Methods* **13**, 731–740 (2016).
74. Krämer, A., Green, J., Pollard, J. & Tugendreich, S. Causal analysis approaches in Ingenuity Pathway Analysis. *Bioinforma. Oxf. Engl.* **30**, 523–530 (2014).
75. Holmfeldt, L. *et al.* The genomic landscape of hypodiploid acute lymphoblastic leukemia. *Nat. Genet.* **45**, 242–252 (2013).

Supplementary Figures

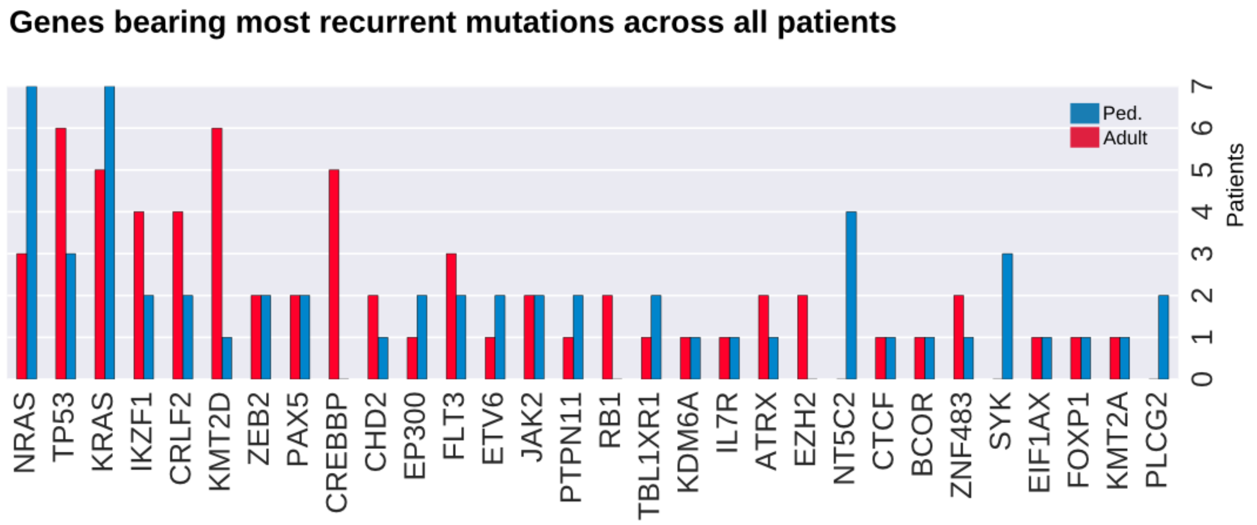

Figure S1: Mutational burden and gene mutation recurrence in adult and pediatric patients. The most recurrently mutated genes (n >= 3) associated to cancer and their occurrence in the pediatric and adult samples. The most recurrent genes have been prioritized by implication in leukemia and other cancer entities. The most frequently mutated genes associated with cancer (see methods) amongst adult and pediatric patients were KRAS (28% of patients), NRAS (20%), TP53 (18%), KMT2D (14%), IKZF1 (12%), JAK2 (10%), CREBBP (10%) and FLT3 (10%). NT5C2 and SYK were exclusively mutated in the pediatric (n>=3) cohort samples and CREBBP and CHD2 (n>=3) in the adult cohort.

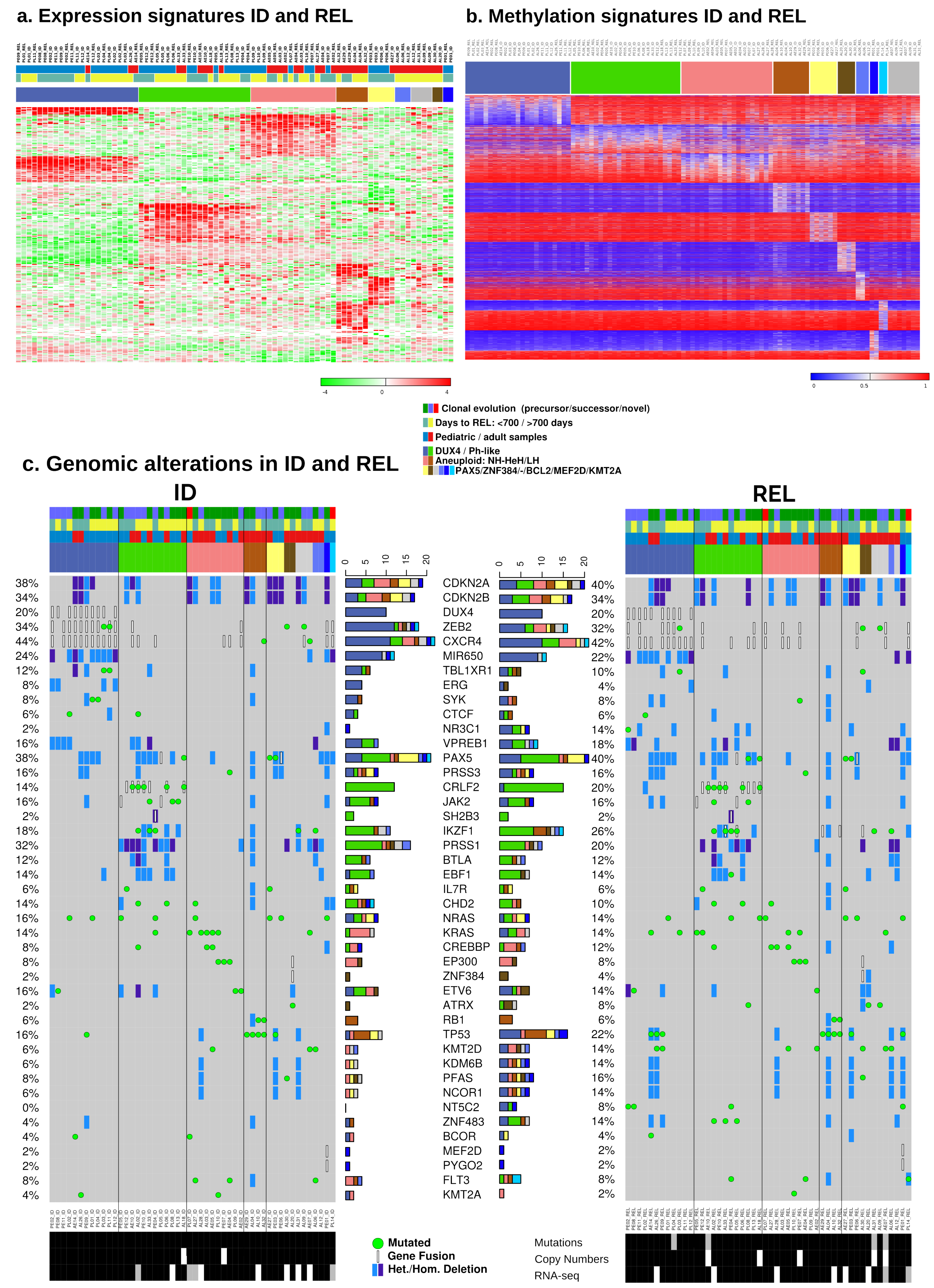

a. Expression signatures ID and REL
b. Methylation signatures ID and REL
-4
0
4
0
0.5
1
Clonal evolution (precursor/successor/novel)
Days to REL: <700 / >700 days
Pediatric / adult samples
DUX4 / Ph-like
Aneuploid: NH-HeH/LH
PAX5/ZNF384/-/BCL2/MEF2D/KMT2A
c. Genomic alterations in ID and REL
ID
REL
CDKN2A
CDKN2B
DUX4
ZEB2
CXCR4
MIR650
TBL1XR1
ERG
SYK
CTCF
NR3C1
VPREB1
PAX5
PRSS3
CRLF2
JAK2
SH2B3
IKZF1
PRSS1
BTLA
EBF1
IL7R
CHD2
NRAS
KRAS
CREBBP
EP300
ZNF384
ETV6
ATRX
RB1
TP53
KMT2D
KDM6B
PFAS
NCOR1
NT5C2
ZNF483
BCOR
MEF2D
PYGO2
FLT3
KMT2A
Mutated
Gene Fusion
Het./Hom. Deletion
Mutations
Copy Numbers
RNA-seq

Figure S2: Gene expression, DNA methylation and somatic alteration signatures of the various BCP-ALL subtypes. The samples of all subfigures have been ordered by their BCP-ALL subgroup assignment. (a) and (b) show the expression and methylation signatures for the classified subgroups. The expression data show gene-centered fold change of log2 transcripts per million (TPM). The methylation is represented by the beta values. Both subfigures show the subtype-specific altered genes and CpGs respectively, grouped accordingly to subtype-specificity. (c) Genomic alterations in molecular subtypes (mutations, fusions, deletions and alternative transcripts; legend below) in ID and REL of adult and pediatric BCP-ALL. The percentage on the left shows the frequency of samples bearing an alteration, and the bar plots show in which BCP-ALL subtypes they occur indicated by color-coding (legend above). The lowest part of each heatmap shows the analyses that have been performed for each sample. The black color stands for normal analysis, white stands for unavailable data and gray stands for limited analyses (see methods).

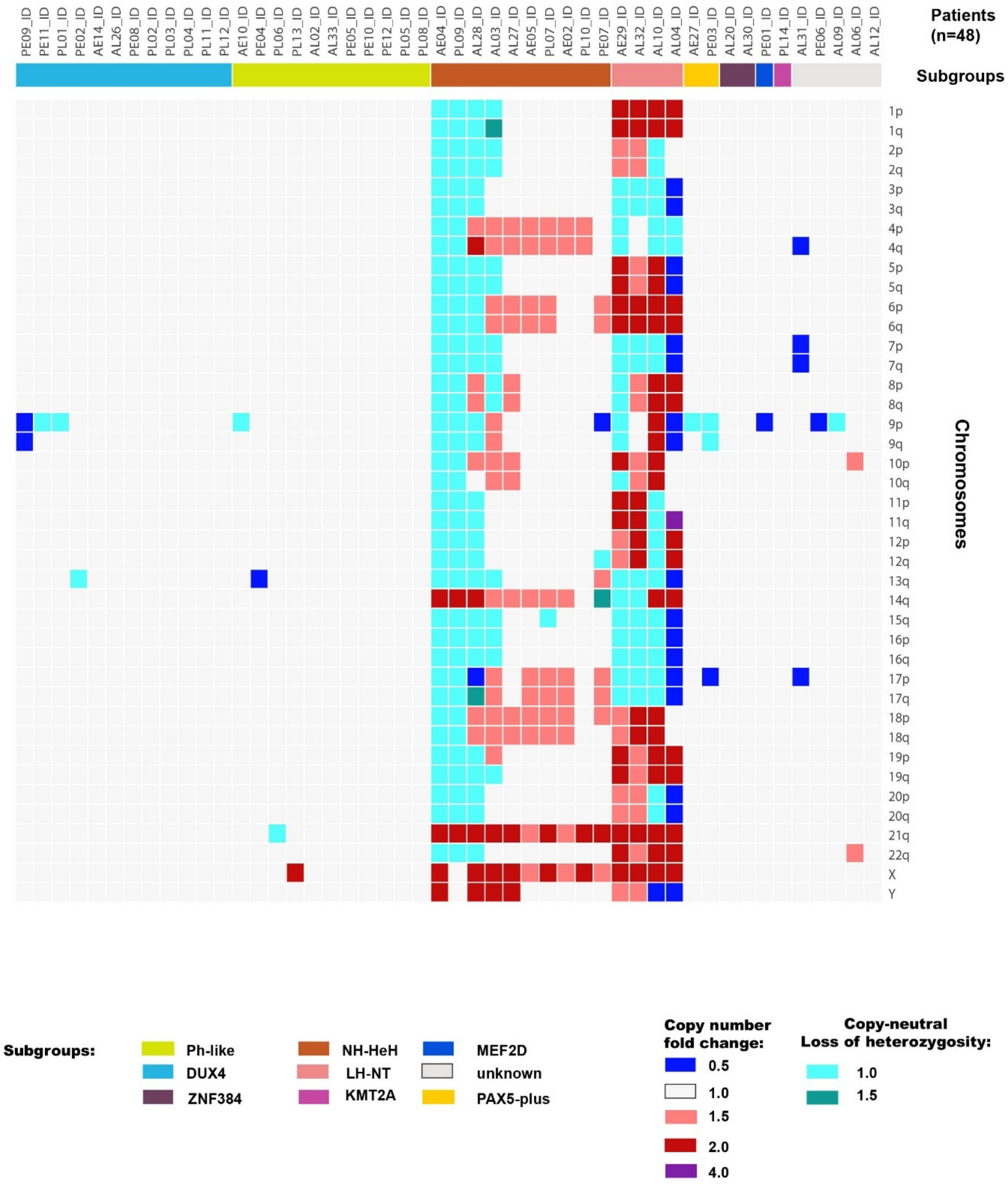

Figure S3: Chromosomal status for 48 samples ID of the cohort, including 14 aneuploid patients, 10 of which were classified as NH-HeH and 4 of which were classified as LH.

a. Clonal evolution of recurrently mutated genes with a volatile pattern

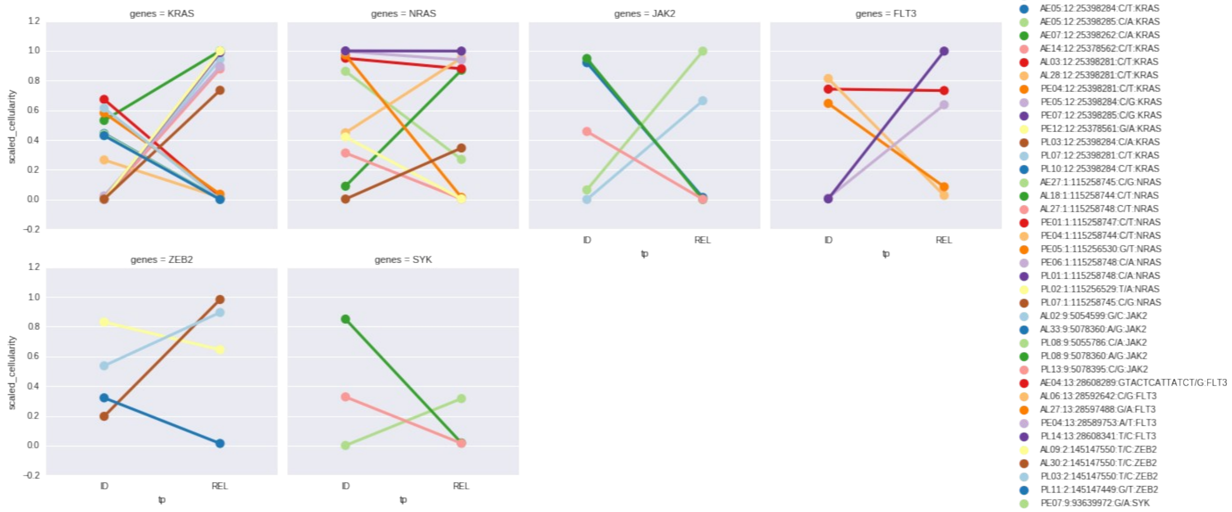

b. Clonal evolution of recurrently mutated genes with a stabilizing pattern

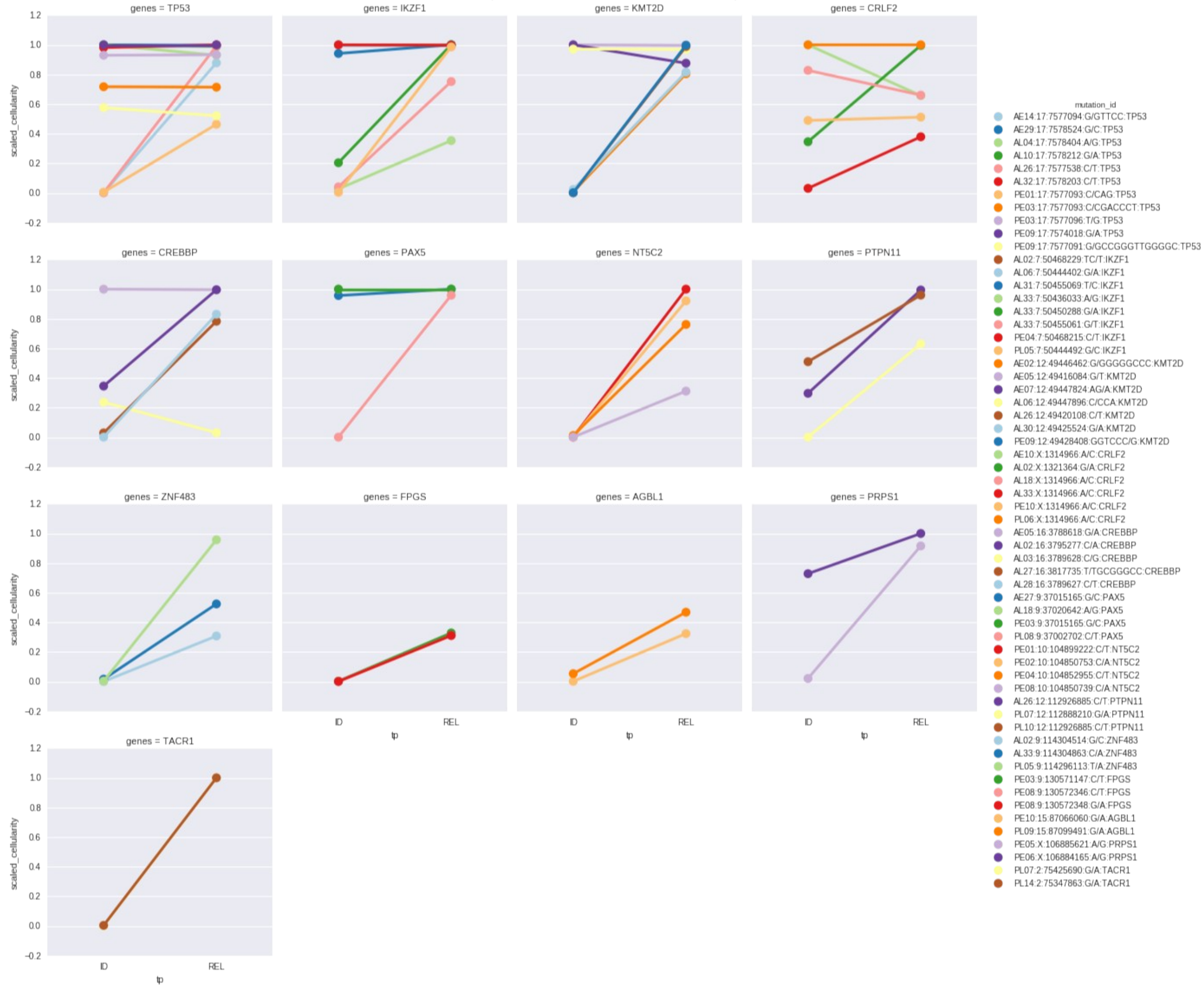

Figure S4: Clonal evolution of mutations of genes with volatile and stabilizing patterns. (a) Clonal evolution of REL-specificof most recurrently mutated genes with a volatile pattern. (b) Clonal evolution of REL-specific mutations in genes identified by high recurrence in REL, high cellularity in REL-specific mutations or REL-specific double-hit: PTPN11, SYK, CXCR4, LAMA2, LAMA1, TACR1 and PRPS1 bearing a stabilizing pattern.

Clonal evolution in PL11

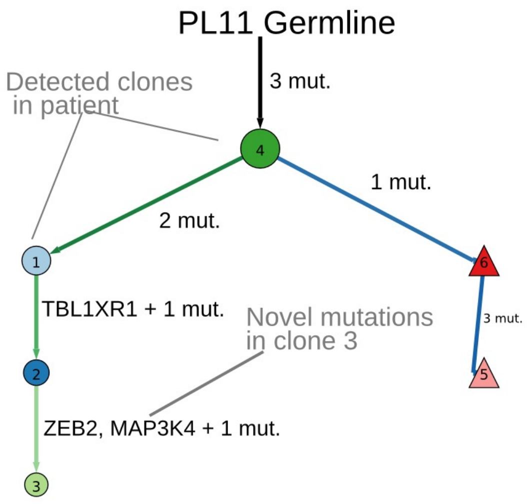

Figure S5: This figure shows the clonal evolution trees between ID and REL for the patient PL11 calculated with PyClone and Schism. The GL node represents the germline, the starting point. The greener the arrow, the more specific to ID, the bluer the arrow the more specific to REL. The darker the luminosity reflects the higher cellularity of the clone. These highlighted genes represent genes in our curated cancer gene (Supplementary Table S10) and genes of which one copy has been lost according to the CNA analysis.

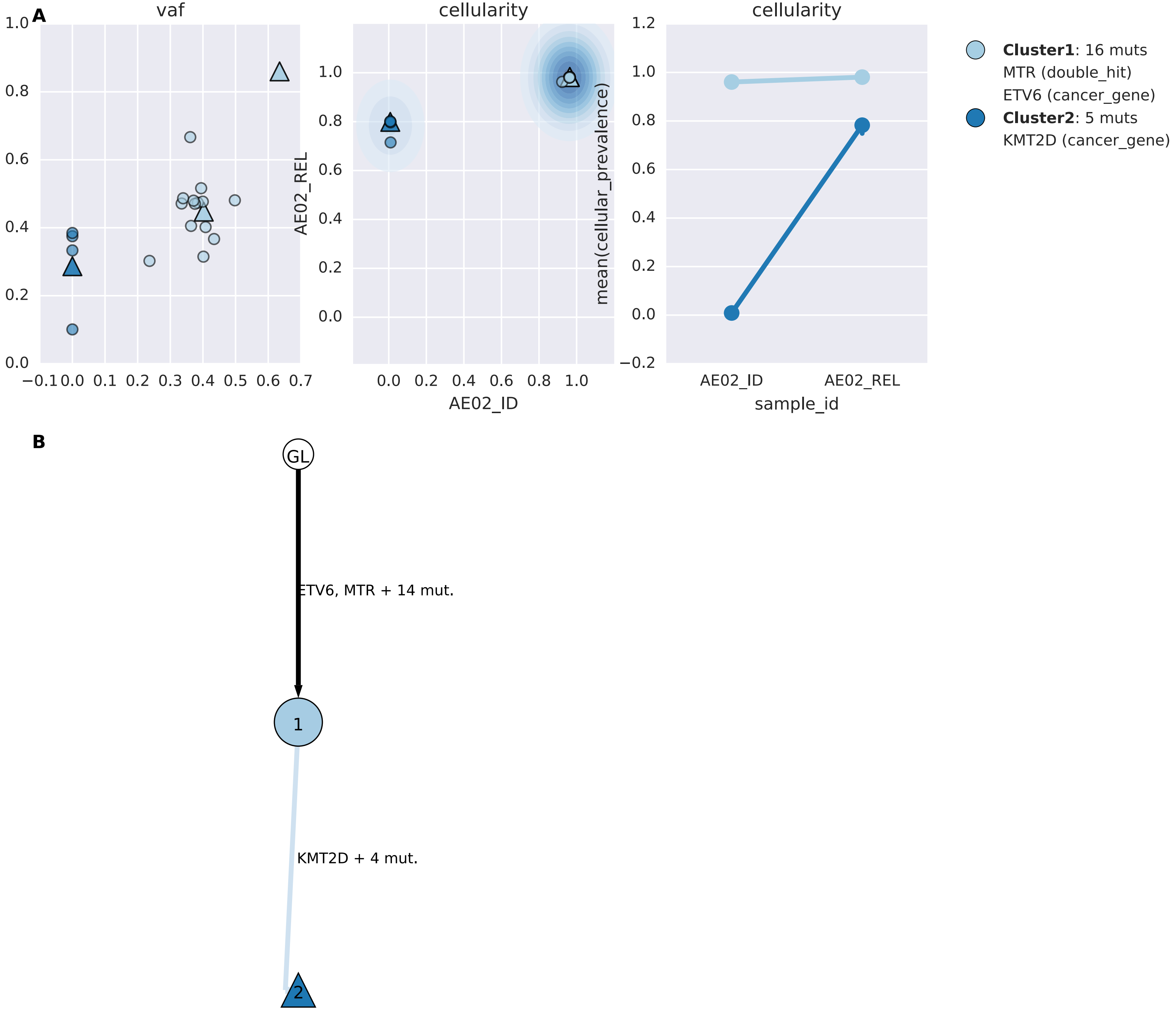
A
vaf
cellularity
AE02_REL
AE02_ID
cellularity
mean(cellular_prevalence)
sample_id
AE02_ID
AE02_REL
Cluster1: 16 muts
MTR (double_hit)
ETV6 (cancer_gene)
Cluster2: 5 muts
KMT2D (cancer_gene)
B
GL
ETV6, MTR + 14 mut.
1
KMT2D + 4 mut.
2

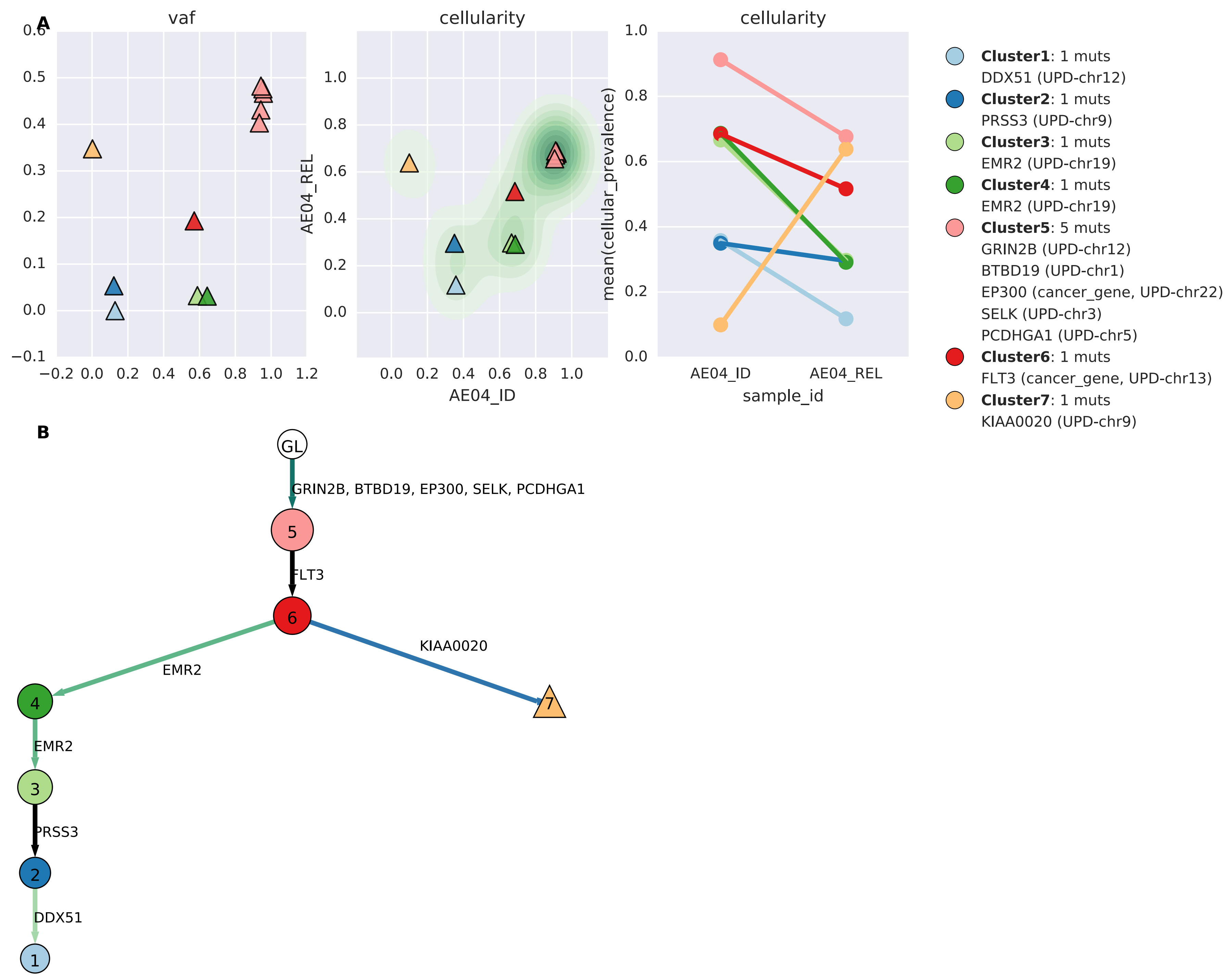
A
vaf
cellularity
AE04_ID
AE04_REL
cellularity
mean(cellular_prevalence)
AE04_ID
AE04_REL
sample_id
Cluster1: 1 muts
DDX51 (UPD-chr12)
Cluster2: 1 muts
PRSS3 (UPD-chr9)
Cluster3: 1 muts
EMR2 (UPD-chr19)
Cluster4: 1 muts
EMR2 (UPD-chr19)
Cluster5: 5 muts
GRIN2B (UPD-chr12)
BTBD19 (UPD-chr1)
EP300 (cancer_gene, UPD-chr22)
SELK (UPD-chr3)
PCDHGA1 (UPD-chr5)
Cluster6: 1 muts
FLT3 (cancer_gene, UPD-chr13)
Cluster7: 1 muts
KIAA0020 (UPD-chr9)
B
GL
GRIN2B, BTBD19, EP300, SELK, PCDHGA1
5
FLT3
6
EMR2
KIAA0020
4
7
EMR2
3
PRSS3
2
DDX51
1

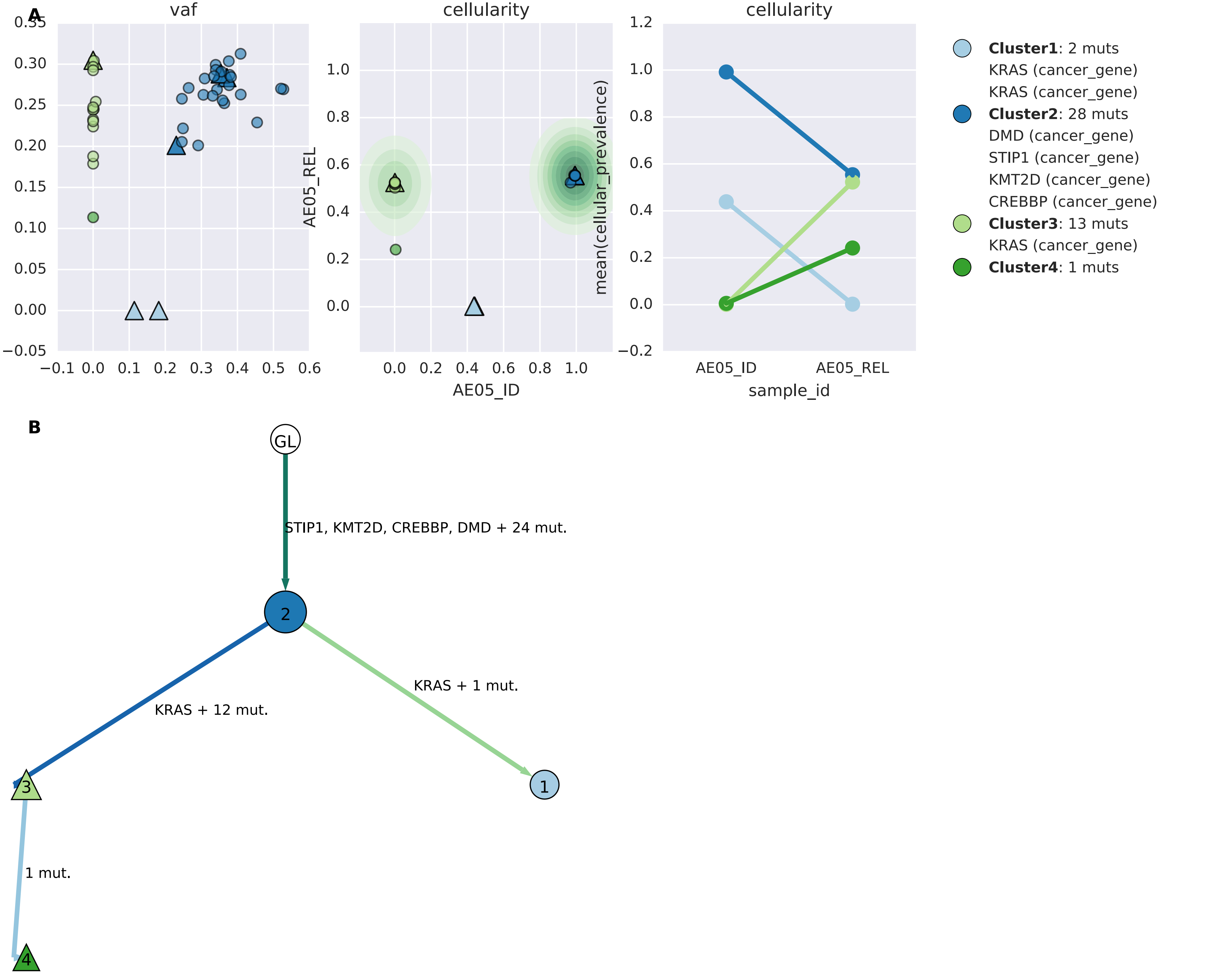

A
vaf
cellularity
AE05_ID
AE05_REL
cellularity
mean(cellular_prevalence)
sample_id
AE05_ID
AE05_REL
Cluster1: 2 muts
KRAS (cancer_gene)
KRAS (cancer_gene)
Cluster2: 28 muts
DMD (cancer_gene)
STIP1 (cancer_gene)
KMT2D (cancer_gene)
CREBBP (cancer_gene)
Cluster3: 13 muts
KRAS (cancer_gene)
Cluster4: 1 muts
B
GL
STIP1, KMT2D, CREBBP, DMD + 24 mut.
2
KRAS + 12 mut.
KRAS + 1 mut.
3
1
1 mut.
4

A

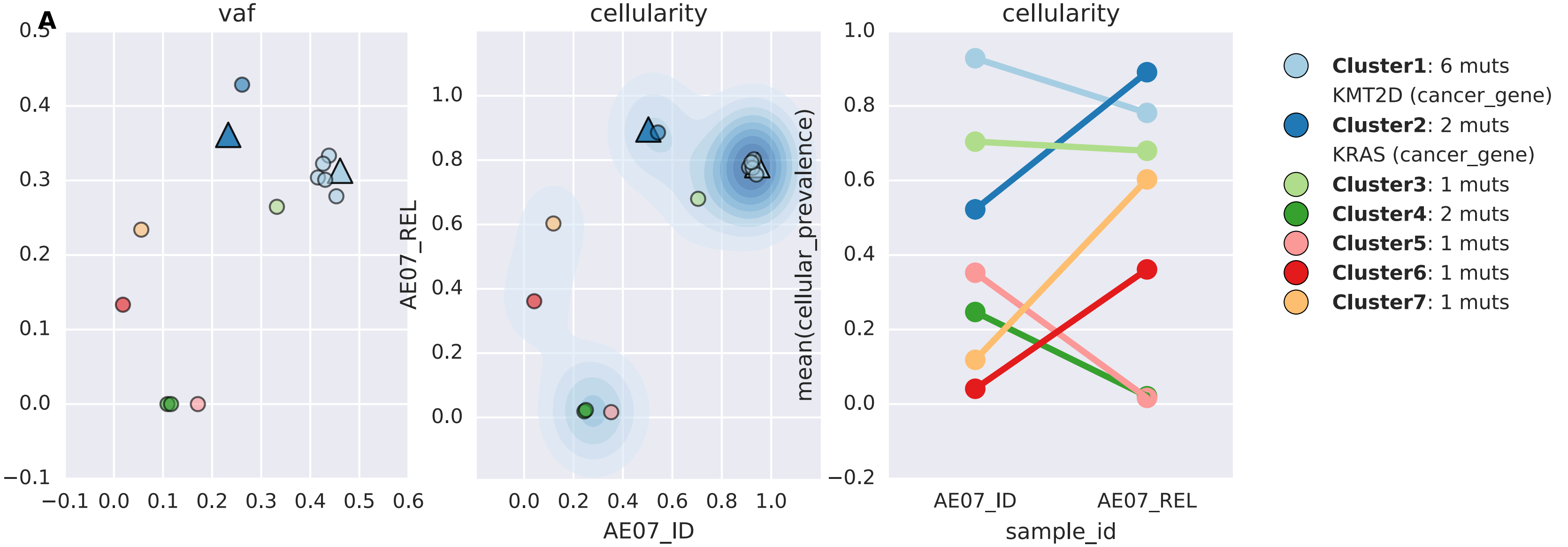

vaf
cellularity
AE07_REL
AE07_ID
cellularity
mean(cellular_prevalence)
AE07_ID
AE07_REL
sample_id
Cluster1: 6 muts
KMT2D (cancer_gene)
Cluster2: 2 muts
KRAS (cancer_gene)
Cluster3: 1 muts
Cluster4: 2 muts
Cluster5: 1 muts
Cluster6: 1 muts
Cluster7: 1 muts

B

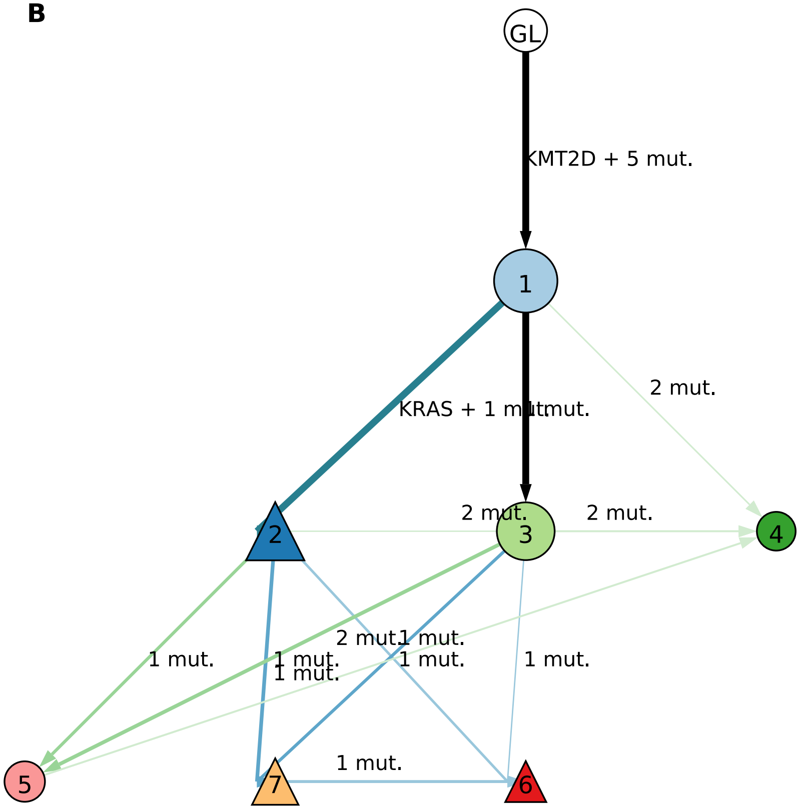

GL
KMT2D + 5 mut.
1
KRAS + 1 mut.
1 mut.
2 mut.
2
3
4
2 mut.
2 mut.
1 mut.
1 mut.
1 mut.
2 mut.
1 mut.
1 mut.
1 mut.
1 mut.
5
7
6

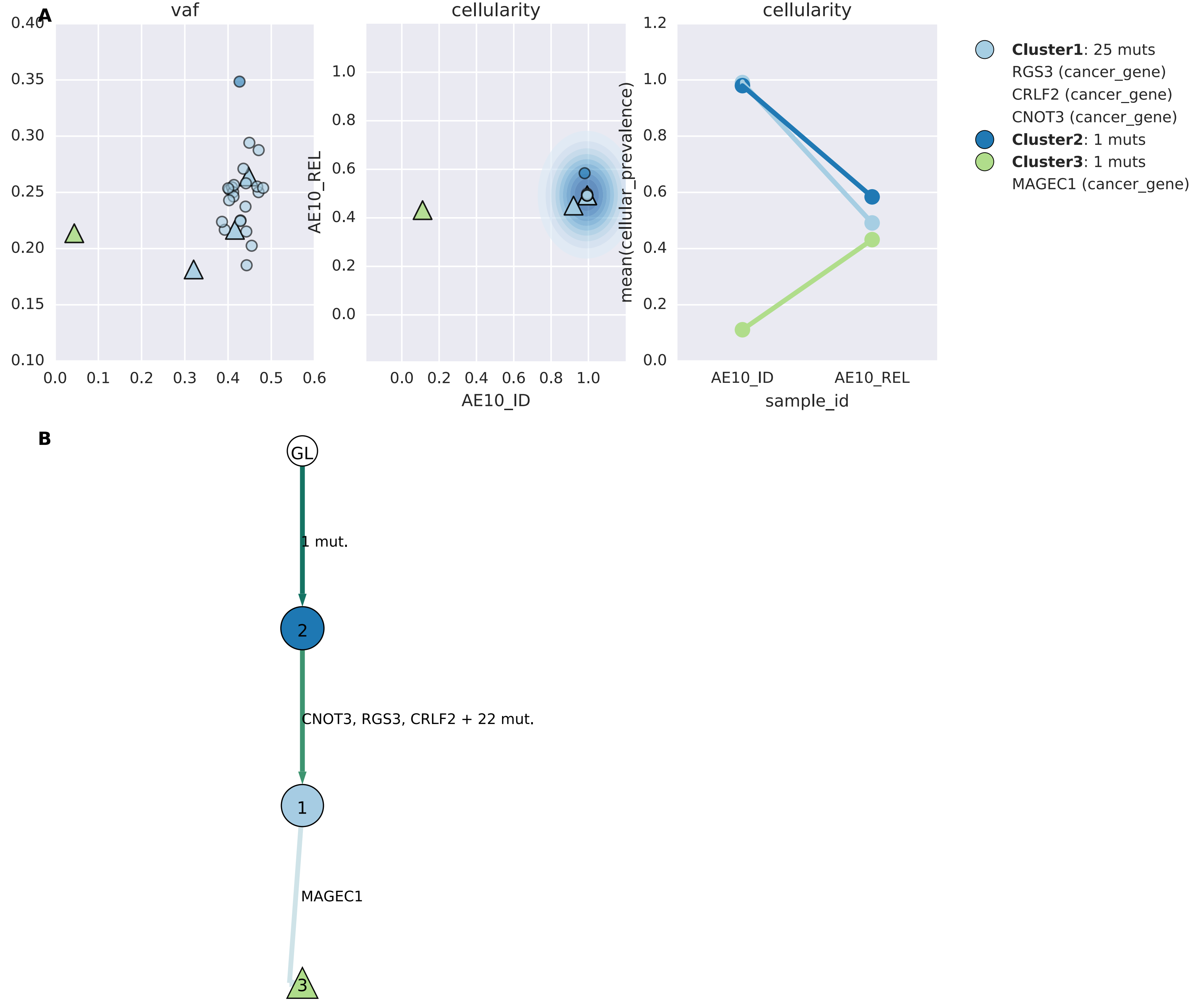
A
vaf
cellularity
AE10_REL
AE10_ID
cellularity
mean(cellular_prevalence)
AE10_ID
AE10_REL
sample_id
Cluster1: 25 muts
RGS3 (cancer_gene)
CRLF2 (cancer_gene)
CNOT3 (cancer_gene)
Cluster2: 1 muts
Cluster3: 1 muts
MAGEC1 (cancer_gene)
B
GL
1 mut.
2
CNOT3, RGS3, CRLF2 + 22 mut.
1
MAGEC1
3

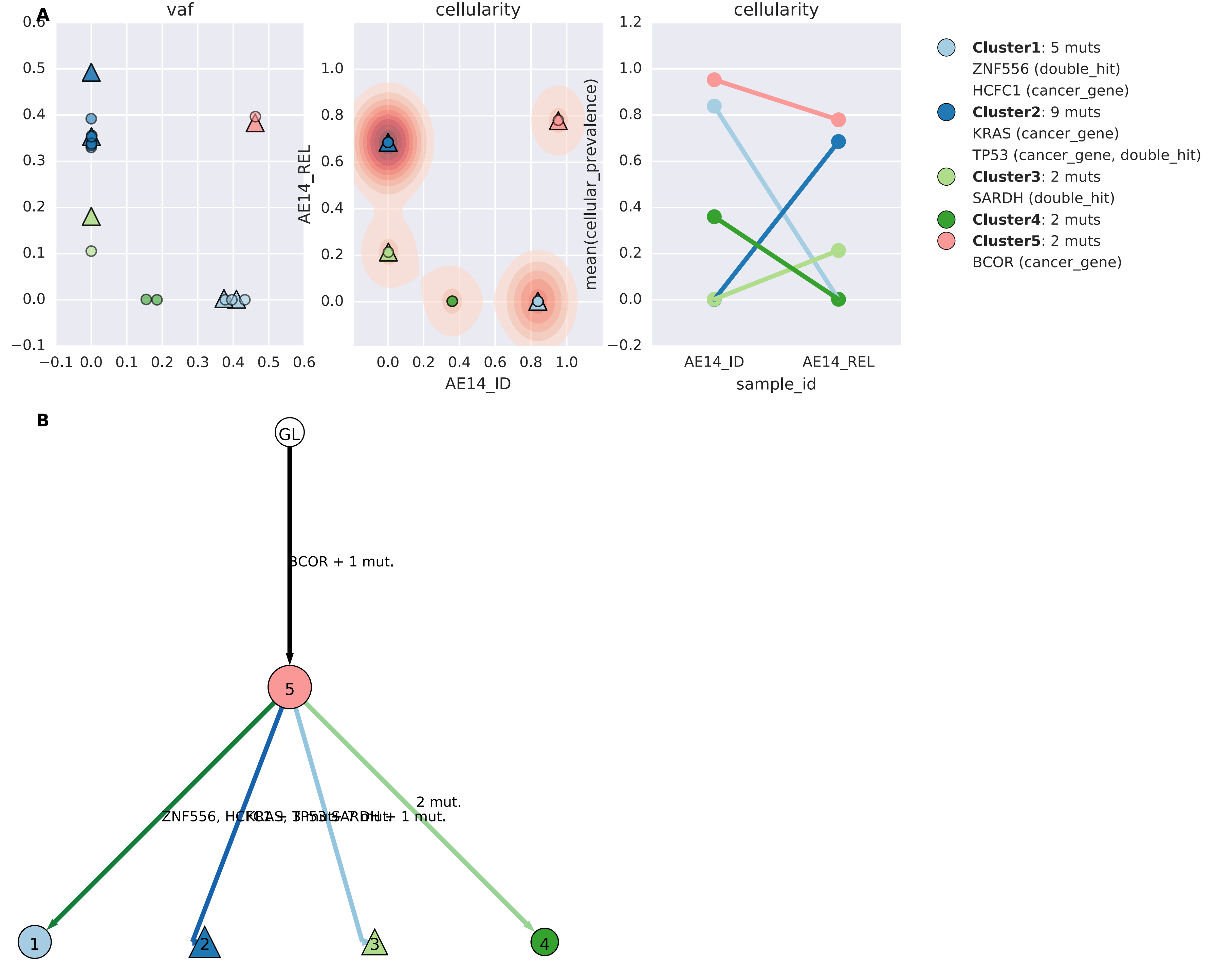

A
vaf
cellularity
cellularity
AE14_REL
AE14_ID
mean(cellular_prevalence)
sample_id
AE14_ID
AE14_REL
Cluster1: 5 muts
ZNF556 (double_hit)
HCFC1 (cancer_gene)
Cluster2: 9 muts
KRAS (cancer_gene)
TP53 (cancer_gene, double_hit)
Cluster3: 2 muts
SARDH (double_hit)
Cluster4: 2 muts
Cluster5: 2 muts
BCOR (cancer_gene)
B
GL
BCOR + 1 mut.
5
2 mut.
1
2
3
4

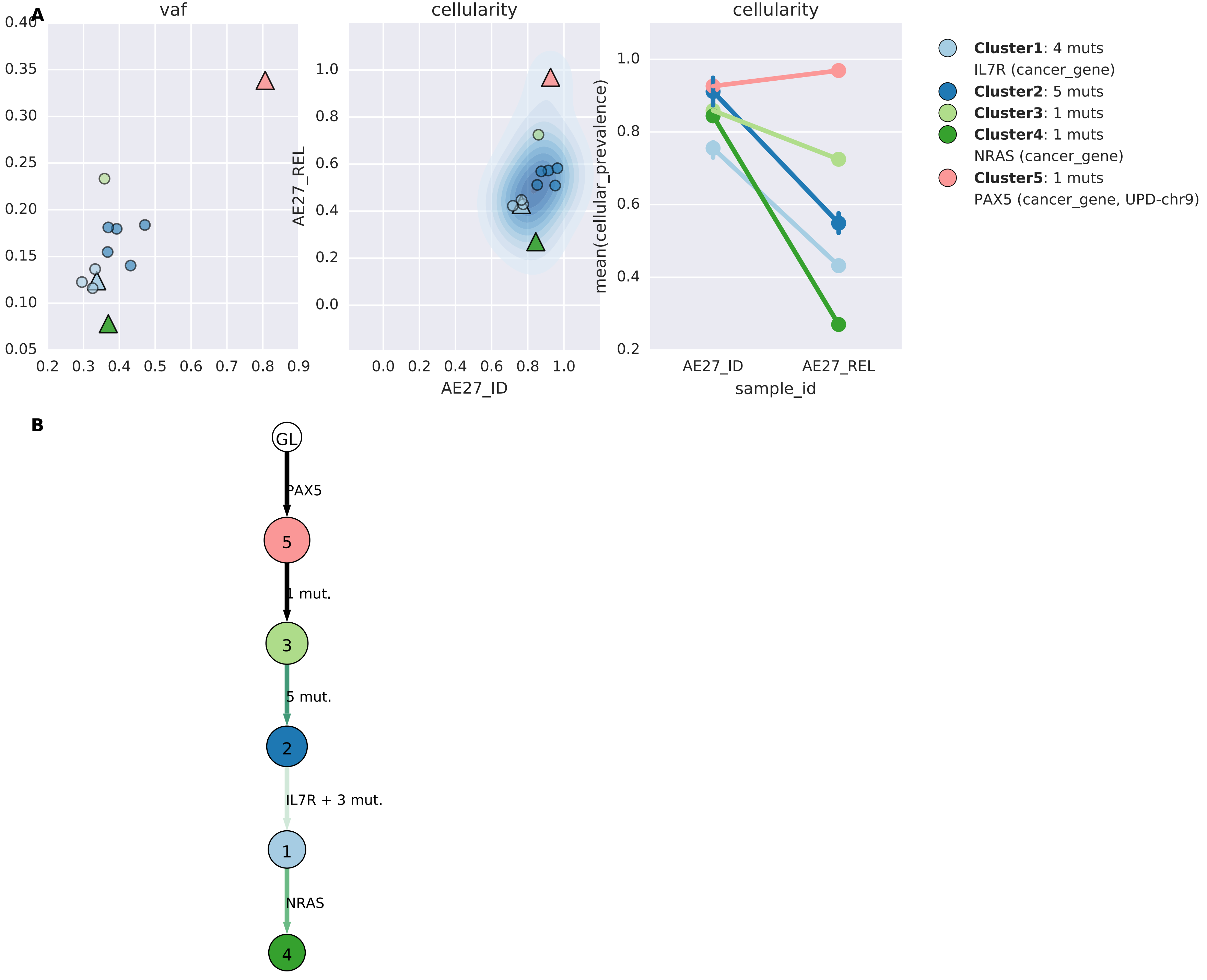
A
vaf
cellularity
cellularity
AE27_REL
AE27_ID
mean(cellular_prevalence)
sample_id
AE27_ID
AE27_REL
0.40
0.35
0.30
0.25
0.20
0.15
0.10
0.05
0.2
0.3
0.4
0.5
0.6
0.7
0.8
0.9
1.0
0.8
0.6
0.4
0.2
0.0
Cluster1: 4 muts
IL7R (cancer_gene)
Cluster2: 5 muts
Cluster3: 1 muts
Cluster4: 1 muts
NRAS (cancer_gene)
Cluster5: 1 muts
PAX5 (cancer_gene, UPD-chr9)
B
GL
PAX5
5
1 mut.
3
5 mut.
2
IL7R + 3 mut.
1
NRAS
4

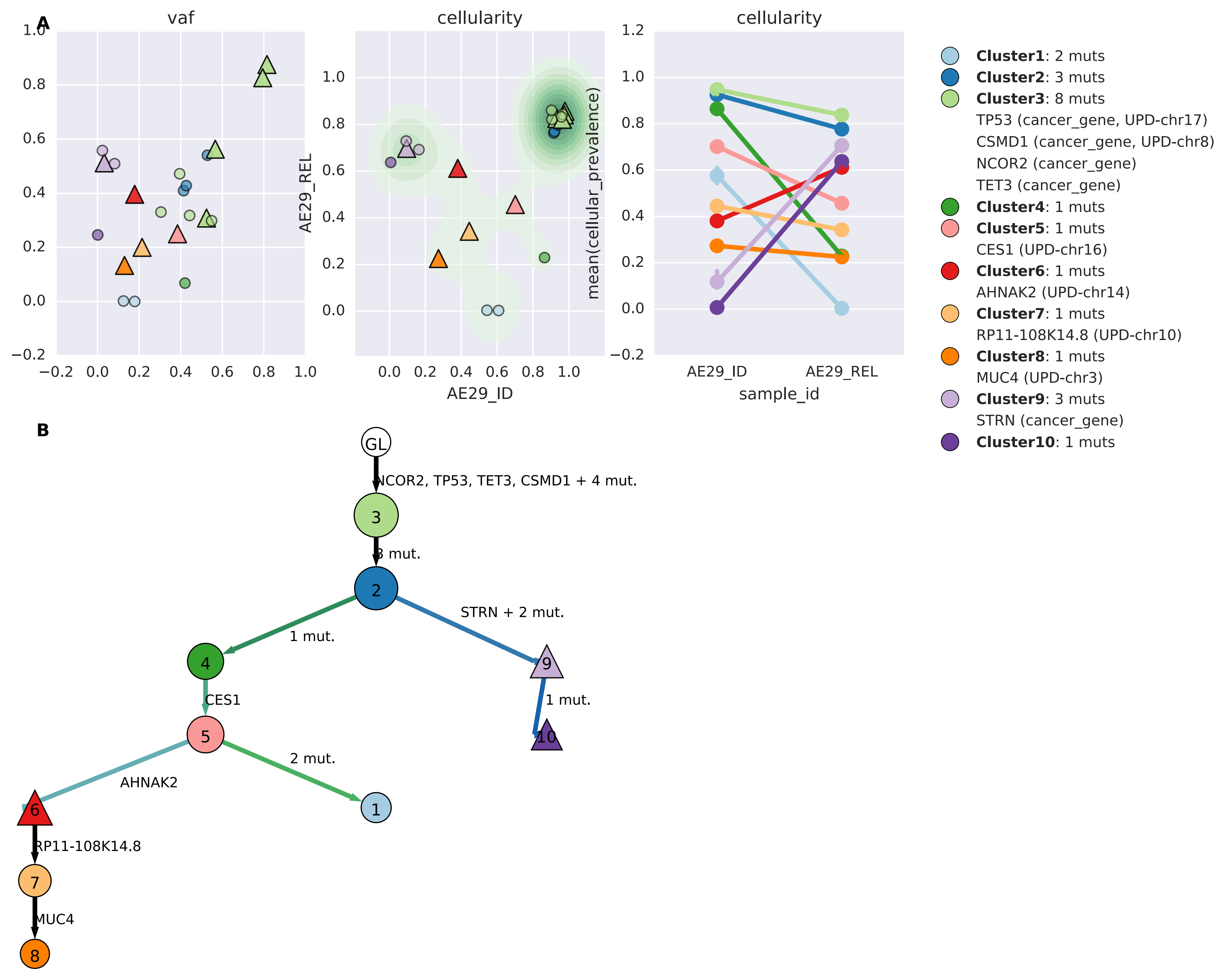
A
vaf
cellularity
AE29_REL
AE29_ID
cellularity
mean(cellular_prevalence)
sample_id
AE29_ID
AE29_REL
Cluster1: 2 muts
Cluster2: 3 muts
Cluster3: 8 muts
TP53 (cancer_gene, UPD-chr17)
CSMD1 (cancer_gene, UPD-chr8)
NCOR2 (cancer_gene)
TET3 (cancer_gene)
Cluster4: 1 muts
Cluster5: 1 muts
CES1 (UPD-chr16)
Cluster6: 1 muts
AHNAK2 (UPD-chr14)
Cluster7: 1 muts
RP11-108K14.8 (UPD-chr10)
Cluster8: 1 muts
MUC4 (UPD-chr3)
Cluster9: 3 muts
STRN (cancer_gene)
Cluster10: 1 muts
B
GL
NCOR2, TP53, TET3, CSMD1 + 4 mut.
3
3 mut.
2
1 mut.
STRN + 2 mut.
4
9
CES1
1 mut.
5
10
2 mut.
AHNAK2
6
1
RP11-108K14.8
7
MUC4
8

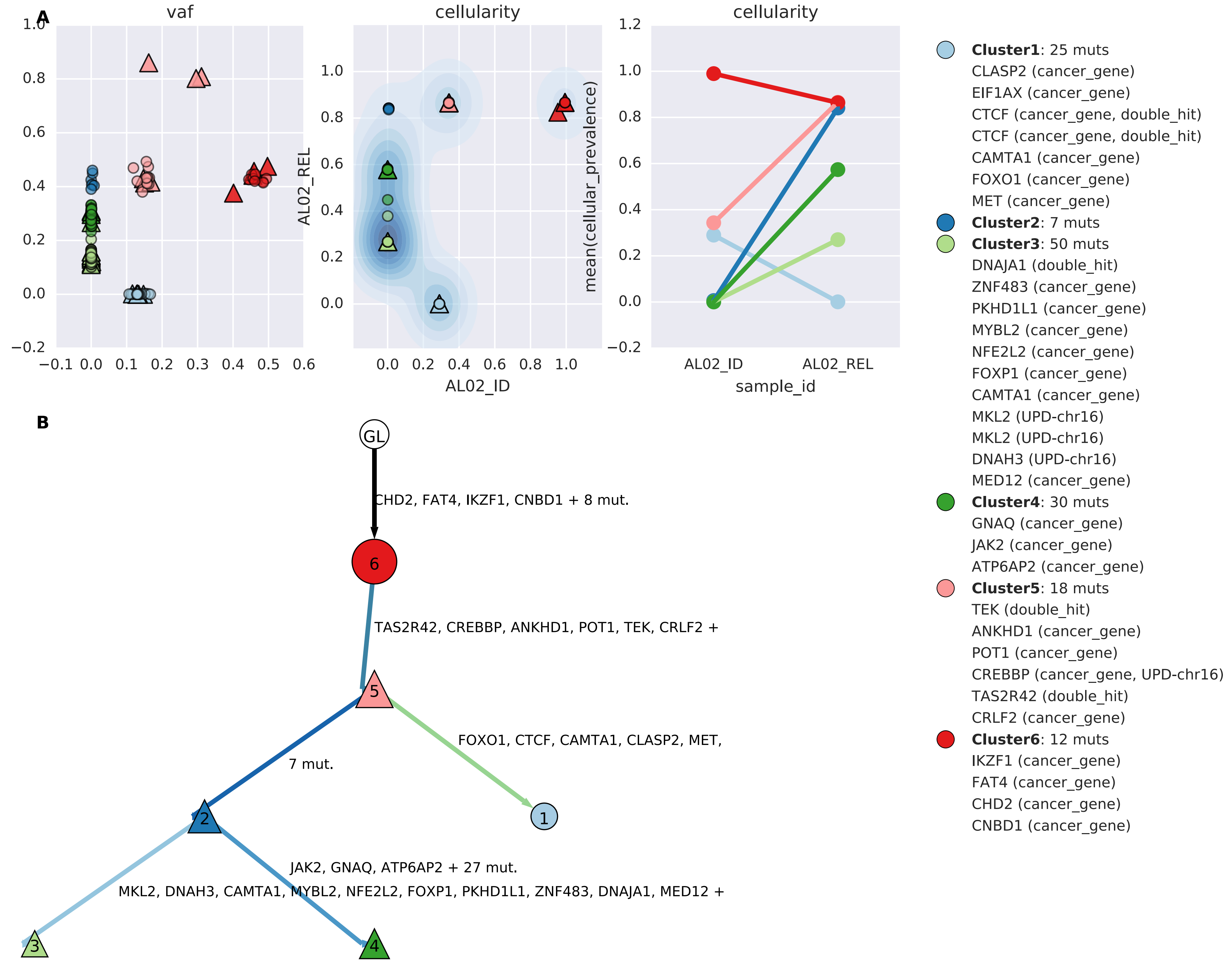
A
vaf
cellularity
cellularity
AL02_REL
AL02_ID
mean(cellular_prevalence)
AL02_ID
AL02_REL
sample_id
Cluster1: 25 muts
CLASP2 (cancer_gene)
EIF1AX (cancer_gene)
CTCF (cancer_gene, double_hit)
CTCF (cancer_gene, double_hit)
CAMTA1 (cancer_gene)
FOXO1 (cancer_gene)
MET (cancer_gene)
Cluster2: 7 muts
Cluster3: 50 muts
DNAJA1 (double_hit)
ZNF483 (cancer_gene)
PKHD1L1 (cancer_gene)
MYBL2 (cancer_gene)
NFE2L2 (cancer_gene)
FOXP1 (cancer_gene)
CAMTA1 (cancer_gene)
MKL2 (UPD-chr16)
MKL2 (UPD-chr16)
DNAH3 (UPD-chr16)
MED12 (cancer_gene)
Cluster4: 30 muts
GNAQ (cancer_gene)
JAK2 (cancer_gene)
ATP6AP2 (cancer_gene)
Cluster5: 18 muts
TEK (double_hit)
ANKHD1 (cancer_gene)
POT1 (cancer_gene)
CREBBP (cancer_gene, UPD-chr16)
TAS2R42 (double_hit)
CRLF2 (cancer_gene)
Cluster6: 12 muts
IKZF1 (cancer_gene)
FAT4 (cancer_gene)
CHD2 (cancer_gene)
CNBD1 (cancer_gene)
B
GL
CHD2, FAT4, IKZF1, CNBD1 + 8 mut.
6
TAS2R42, CREBBP, ANKHD1, POT1, TEK, CRLF2 +
5
FOXO1, CTCF, CAMTA1, CLASP2, MET,
7 mut.
2
1
JAK2, GNAQ, ATP6AP2 + 27 mut.
MKL2, DNAH3, CAMTA1, MYBL2, NFE2L2, FOXP1, PKHD1L1, ZNF483, DNAJA1, MED12 +
3
4

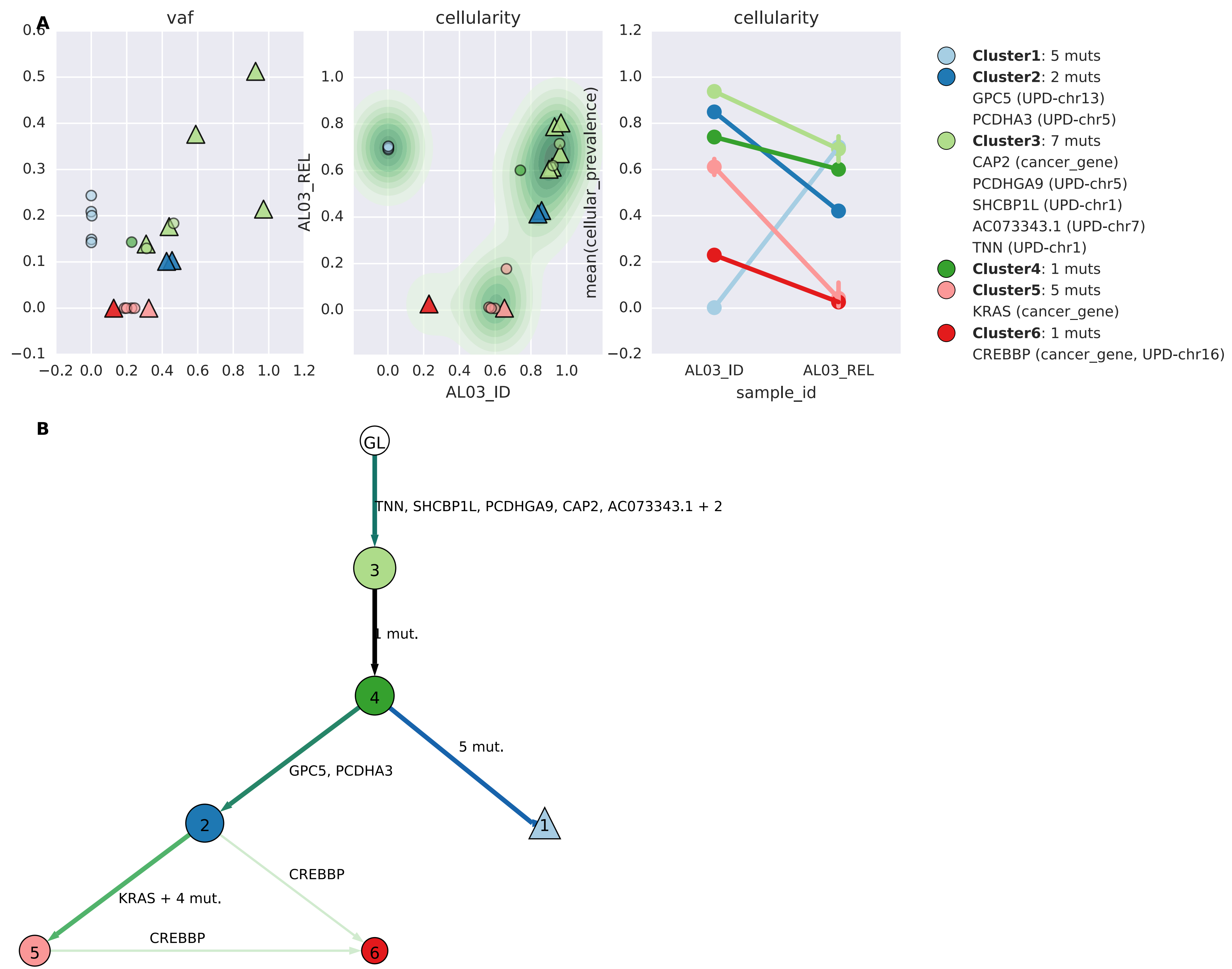
A
vaf
cellularity
cellularity
AL03_REL
AL03_ID
mean(cellular_prevalence)
AL03_ID
AL03_REL
sample_id
Cluster1: 5 muts
Cluster2: 2 muts
GPC5 (UPD-chr13)
PCDHA3 (UPD-chr5)
Cluster3: 7 muts
CAP2 (cancer_gene)
PCDHGA9 (UPD-chr5)
SHCBP1L (UPD-chr1)
AC073343.1 (UPD-chr7)
TNN (UPD-chr1)
Cluster4: 1 muts
Cluster5: 5 muts
KRAS (cancer_gene)
Cluster6: 1 muts
CREBBP (cancer_gene, UPD-chr16)
B
GL
TNN, SHCBP1L, PCDHGA9, CAP2, AC073343.1 + 2
1 mut.
5 mut.
GPC5, PCDHA3
CREBBP
KRAS + 4 mut.
CREBBP

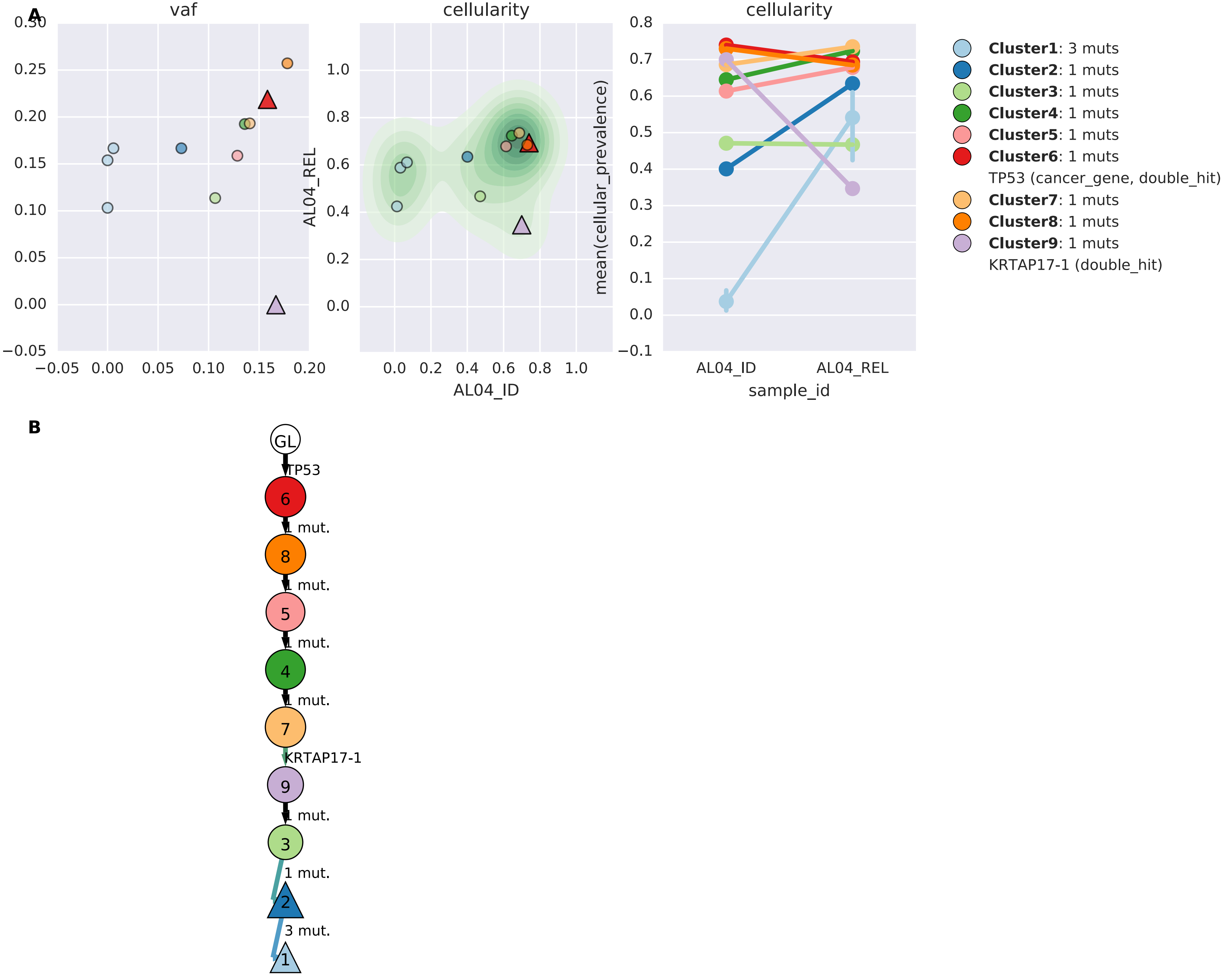
A
vaf
cellularity
AL04_REL
AL04_ID
cellularity
mean(cellular_prevalence)
sample_id
Cluster1: 3 muts
Cluster2: 1 muts
Cluster3: 1 muts
Cluster4: 1 muts
Cluster5: 1 muts
Cluster6: 1 muts
TP53 (cancer_gene, double_hit)
Cluster7: 1 muts
Cluster8: 1 muts
Cluster9: 1 muts
KRTAP17-1 (double_hit)
B
GL
TP53
KRTAP17-1
1 mut.
3 mut.

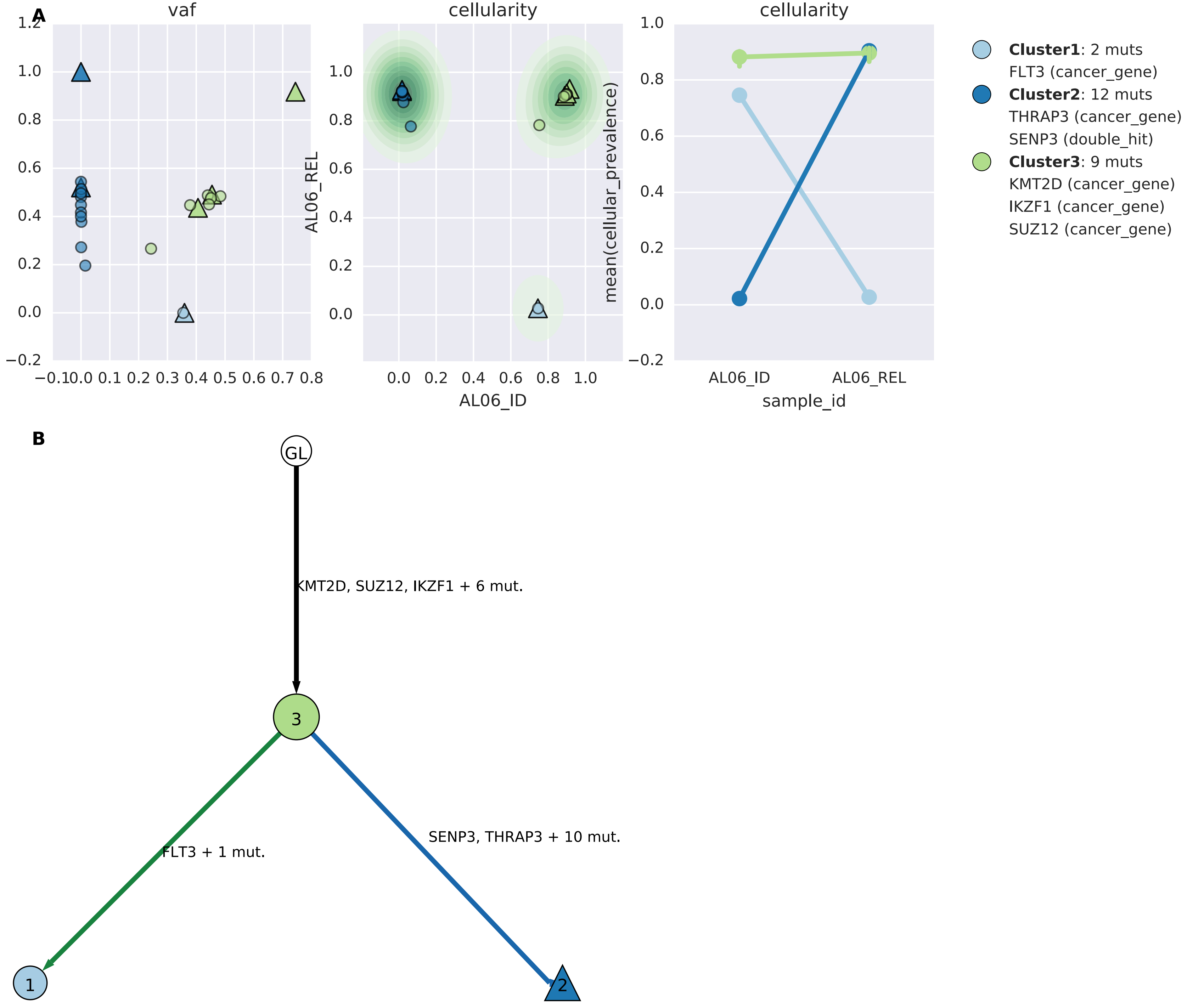
A
vaf
cellularity
AL06_REL
AL06_ID
cellularity
mean(cellular_prevalence)
AL06_ID
AL06_REL
sample_id
Cluster1: 2 muts
FLT3 (cancer_gene)
Cluster2: 12 muts
THRAP3 (cancer_gene)
SENP3 (double_hit)
Cluster3: 9 muts
KMT2D (cancer_gene)
IKZF1 (cancer_gene)
SUZ12 (cancer_gene)
B
GL
KMT2D, SUZ12, IKZF1 + 6 mut.
3
FLT3 + 1 mut.
SENP3, THRAP3 + 10 mut.
1
2

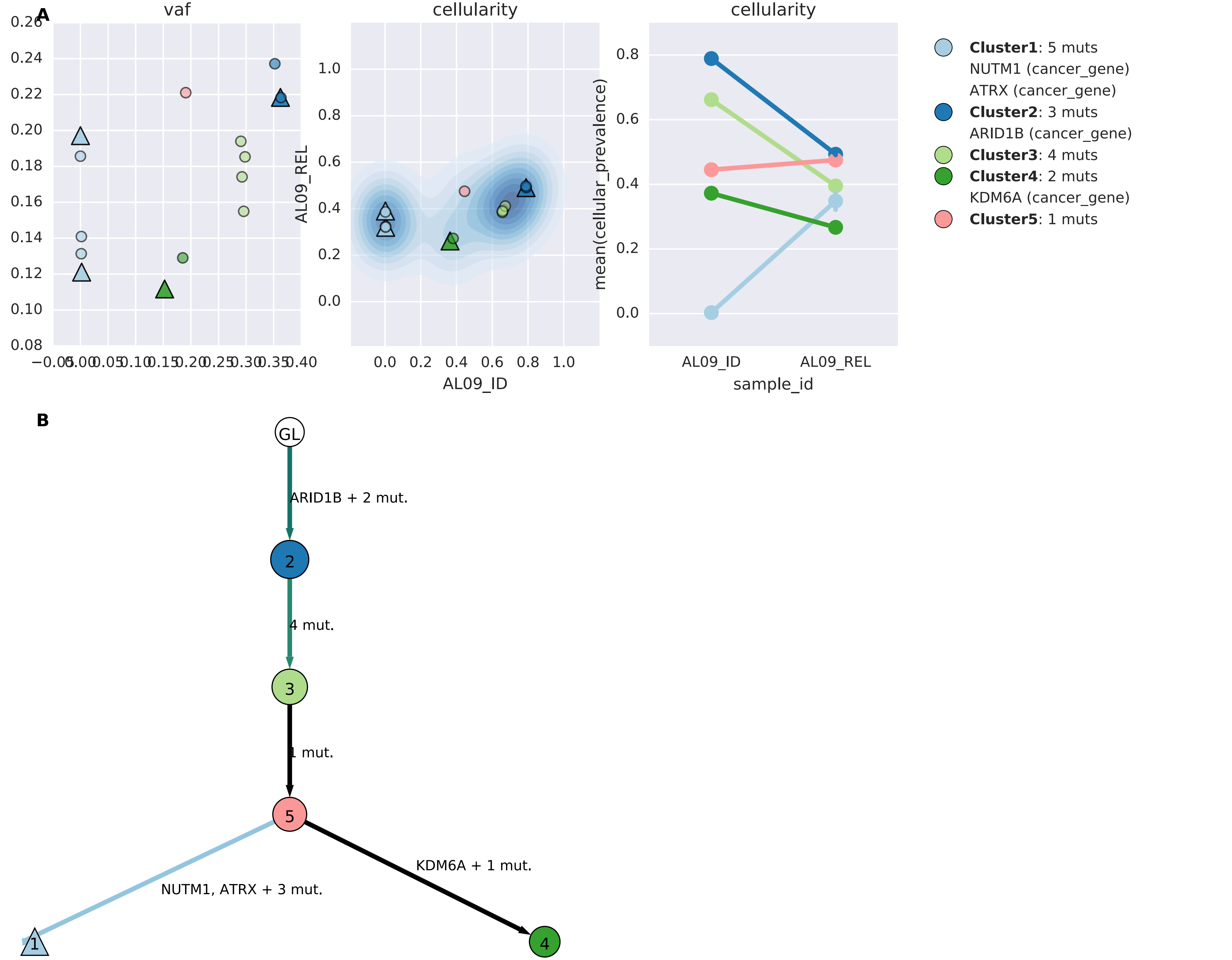
A
vaf
0.26
0.24
0.22
0.20
0.18
0.16
0.14
0.12
0.10
0.08
cellularity
AL09_REL
AL09_ID
1.0
0.8
0.6
0.4
0.2
0.0
cellularity
mean(cellular_prevalence)
AL09_ID
AL09_REL
sample_id
Cluster1: 5 muts
NUTM1 (cancer_gene)
ATRX (cancer_gene)
Cluster2: 3 muts
ARID1B (cancer_gene)
Cluster3: 4 muts
Cluster4: 2 muts
KDM6A (cancer_gene)
Cluster5: 1 muts
B
GL
ARID1B + 2 mut.
2
4 mut.
3
1 mut.
5
NUTM1, ATRX + 3 mut.
KDM6A + 1 mut.
1
4

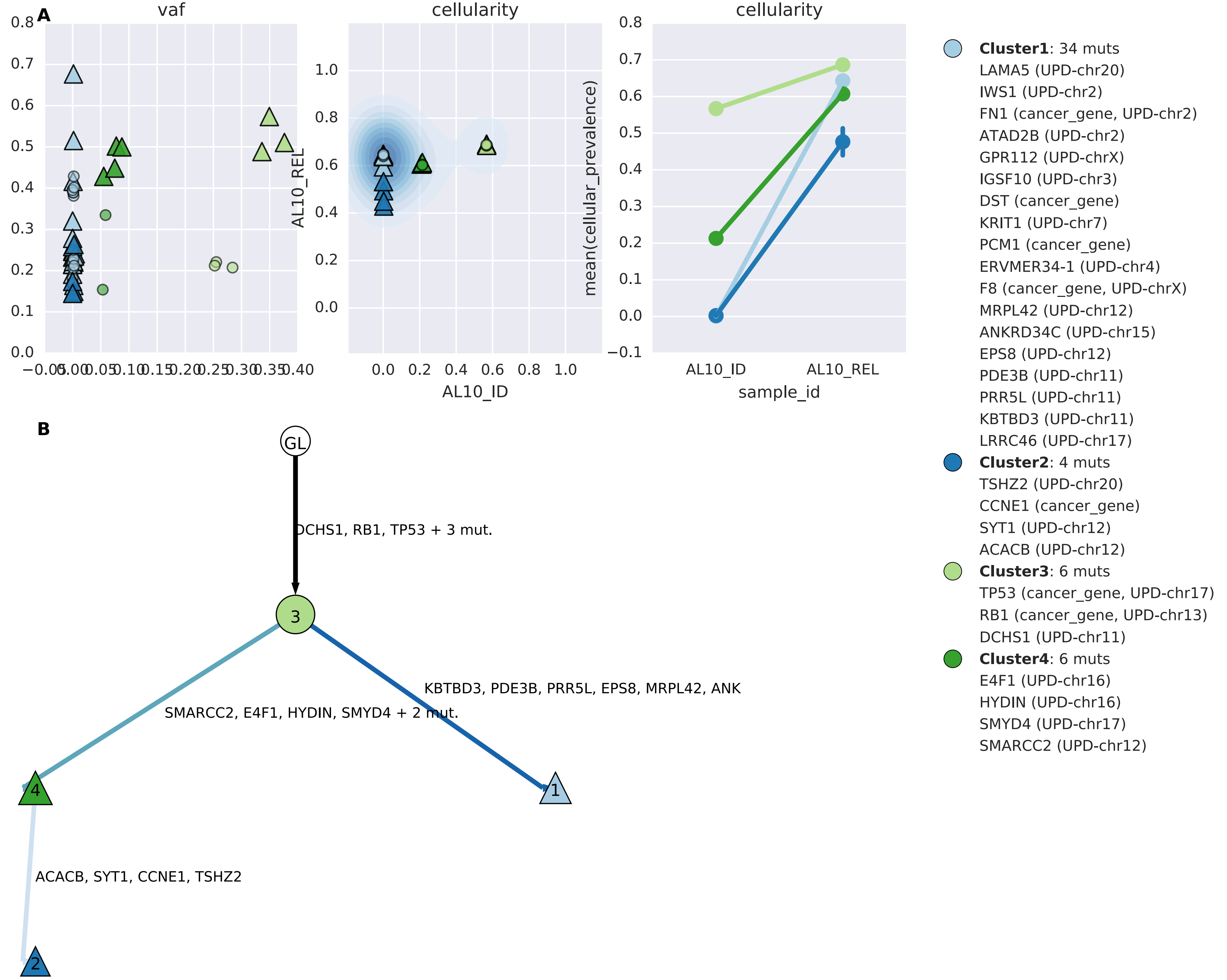

A
vaf
cellularity
cellularity
AL10_REL
AL10_ID
mean(cellular_prevalence)
AL10_ID
AL10_REL
sample_id
Cluster1: 34 muts
LAMA5 (UPD-chr20)
IWS1 (UPD-chr2)
FN1 (cancer_gene, UPD-chr2)
ATAD2B (UPD-chr2)
GPR112 (UPD-chrX)
IGSF10 (UPD-chr3)
DST (cancer_gene)
KRIT1 (UPD-chr7)
PCM1 (cancer_gene)
ERVMER34-1 (UPD-chr4)
F8 (cancer_gene, UPD-chrX)
MRPL42 (UPD-chr12)
ANKRD34C (UPD-chr15)
EPS8 (UPD-chr12)
PDE3B (UPD-chr11)
PRR5L (UPD-chr11)
KBTBD3 (UPD-chr11)
LRRC46 (UPD-chr17)
Cluster2: 4 muts
TSHZ2 (UPD-chr20)
CCNE1 (cancer_gene)
SYT1 (UPD-chr12)
ACACB (UPD-chr12)
Cluster3: 6 muts
TP53 (cancer_gene, UPD-chr17)
RB1 (cancer_gene, UPD-chr13)
DCHS1 (UPD-chr11)
Cluster4: 6 muts
E4F1 (UPD-chr16)
HYDIN (UPD-chr16)
SMYD4 (UPD-chr17)
SMARCC2 (UPD-chr12)
B
GL
DCHS1, RB1, TP53 + 3 mut.
3
SMARCC2, E4F1, HYDIN, SMYD4 + 2 mut.
KBTBD3, PDE3B, PRR5L, EPS8, MRPL42, ANK
4
1
ACACB, SYT1, CCNE1, TSHZ2
2

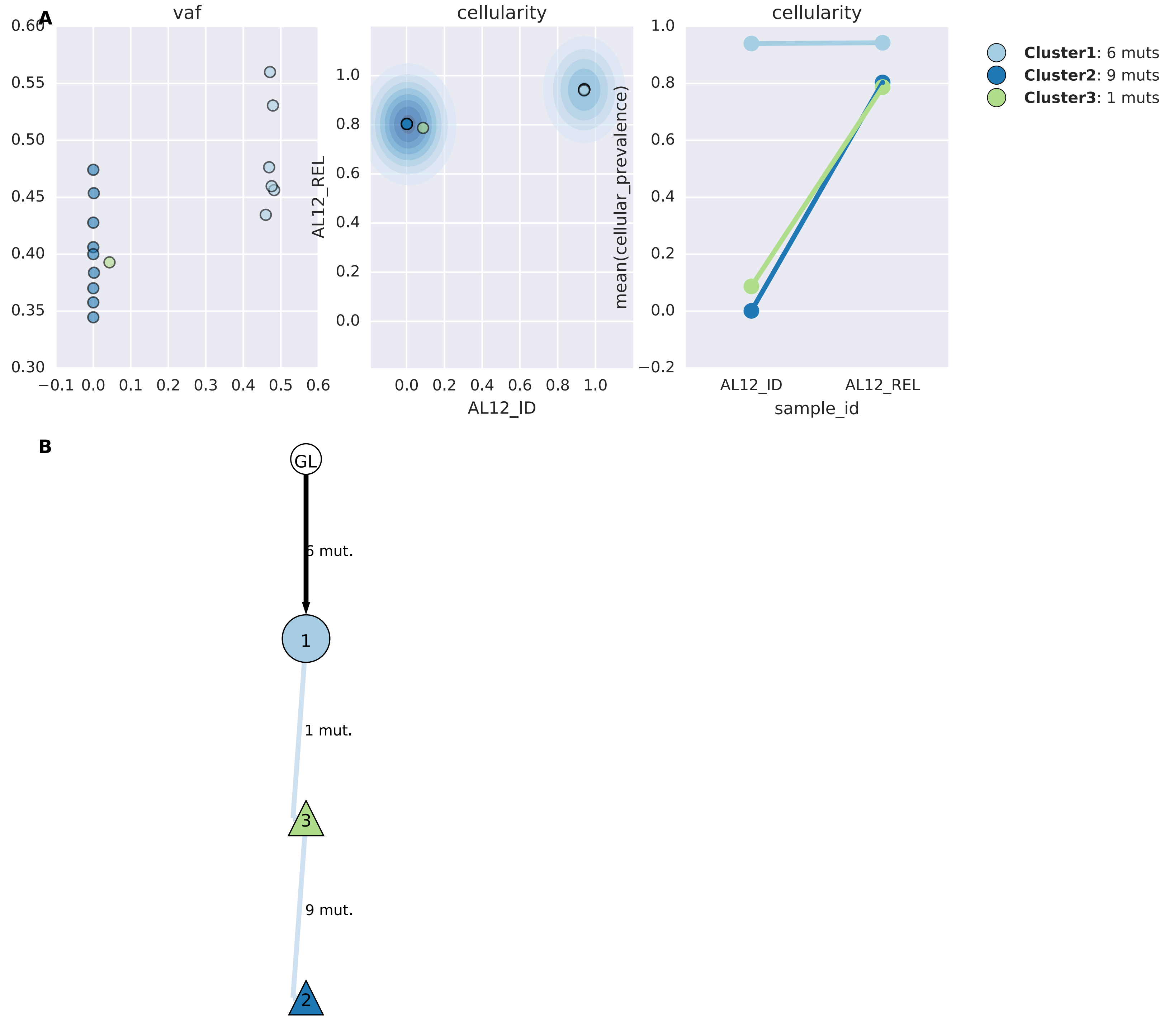
A
vaf
0.60
0.55
0.50
0.45
0.40
0.35
0.30
−0.1 0.0 0.1 0.2 0.3 0.4 0.5 0.6
cellularity
AL12_REL
1.0
0.8
0.6
0.4
0.2
0.0
0.0 0.2 0.4 0.6 0.8 1.0
AL12_ID
cellularity
mean(cellular_prevalence)
1.0
0.8
0.6
0.4
0.2
0.0
−0.2
AL12_ID
AL12_REL
sample_id
Cluster1: 6 muts
Cluster2: 9 muts
Cluster3: 1 muts
B
GL
6 mut.
1
1 mut.
3
9 mut.
2

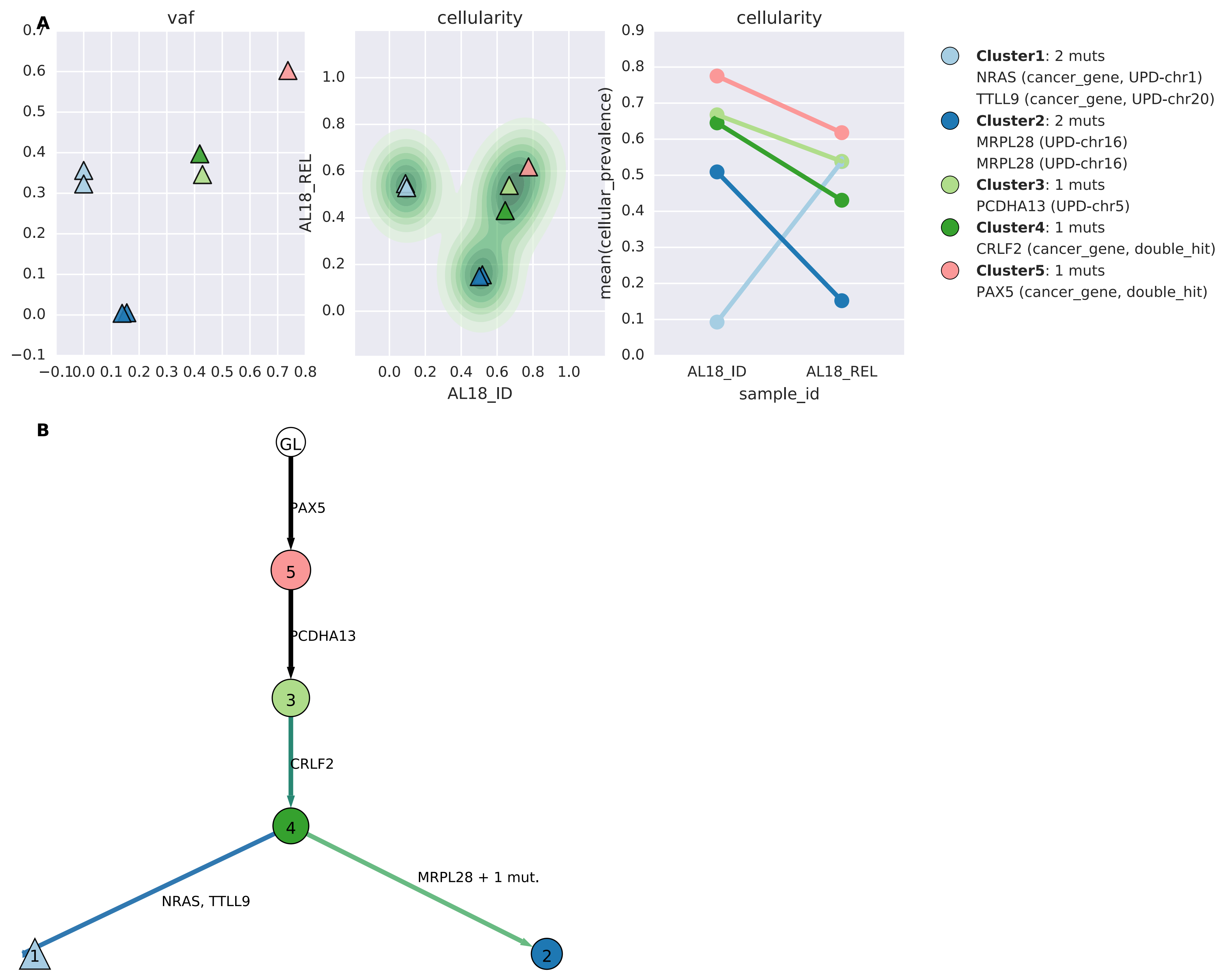
A
vaf
cellularity
cellularity
AL18_REL
AL18_ID
mean(cellular_prevalence)
AL18_ID
AL18_REL
sample_id
Cluster1: 2 muts
NRAS (cancer_gene, UPD-chr1)
TTLL9 (cancer_gene, UPD-chr20)
Cluster2: 2 muts
MRPL28 (UPD-chr16)
MRPL28 (UPD-chr16)
Cluster3: 1 muts
PCDHA13 (UPD-chr5)
Cluster4: 1 muts
CRLF2 (cancer_gene, double_hit)
Cluster5: 1 muts
PAX5 (cancer_gene, double_hit)
B
GL
PAX5
5
PCDHA13
3
CRLF2
4
NRAS, TTLL9
MRPL28 + 1 mut.
1
2

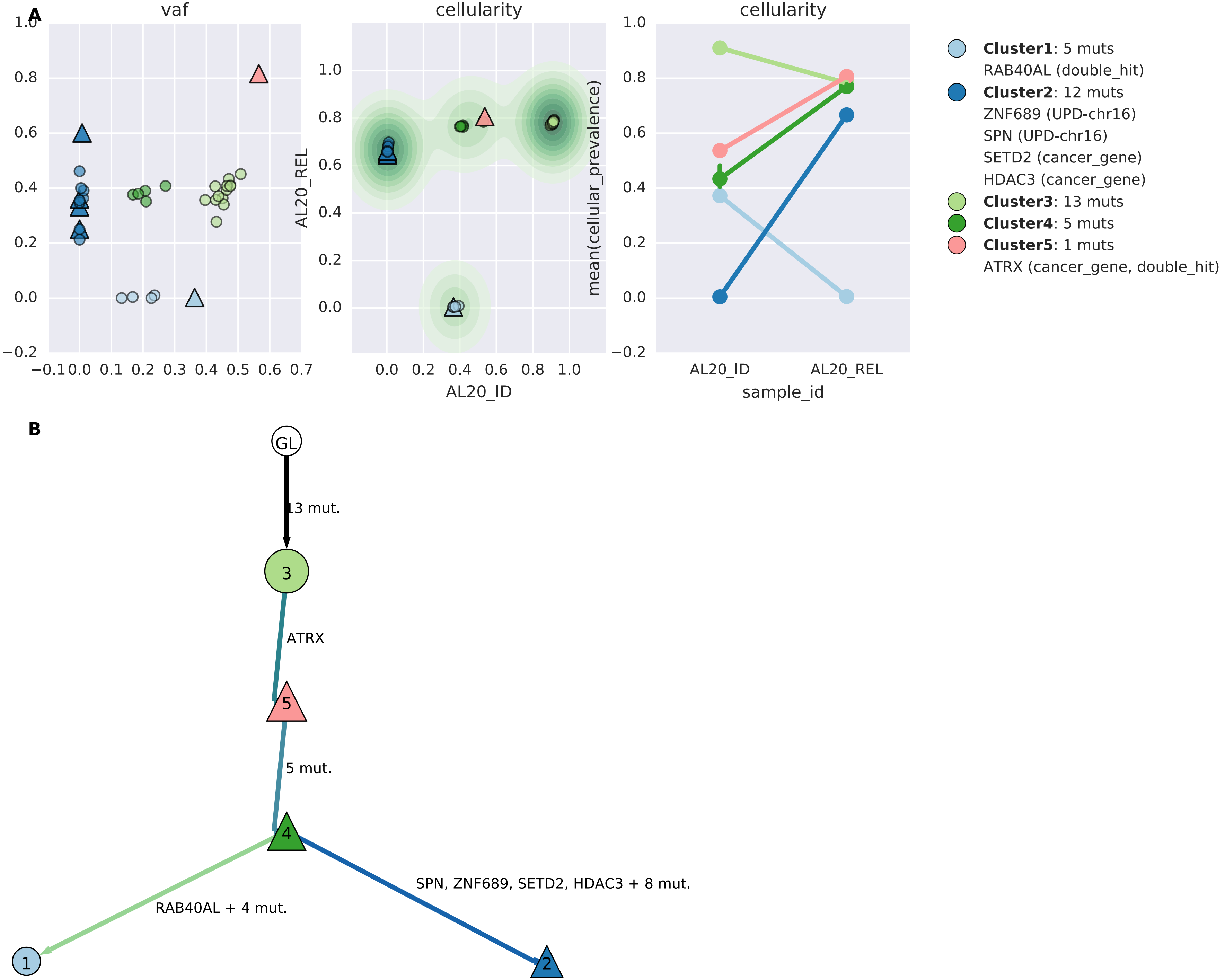

A
vaf
cellularity
cellularity
AL20_REL
AL20_ID
mean(cellular_prevalence)
AL20_ID
AL20_REL
sample_id
Cluster1: 5 muts
RAB40AL (double_hit)
Cluster2: 12 muts
ZNF689 (UPD-chr16)
SPN (UPD-chr16)
SETD2 (cancer_gene)
HDAC3 (cancer_gene)
Cluster3: 13 muts
Cluster4: 5 muts
Cluster5: 1 muts
ATRX (cancer_gene, double_hit)
B
GL
13 mut.
3
ATRX
5
5 mut.
4
RAB40AL + 4 mut.
SPN, ZNF689, SETD2, HDAC3 + 8 mut.
1
2

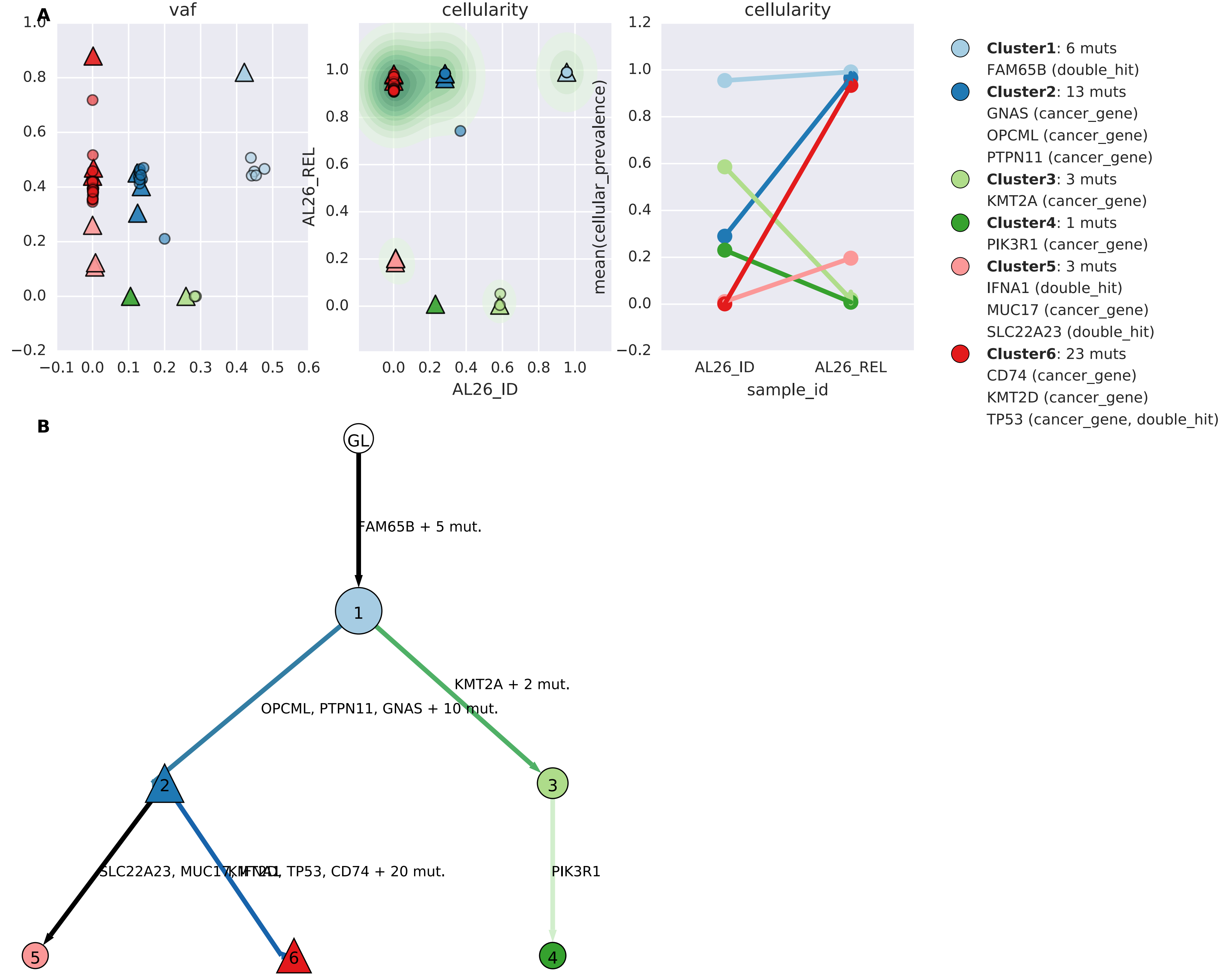
A
vaf
cellularity
AL26_REL
AL26_ID
cellularity
mean(cellular_prevalence)
sample_id
AL26_ID
AL26_REL
Cluster1: 6 muts
FAM65B (double_hit)
Cluster2: 13 muts
GNAS (cancer_gene)
OPCML (cancer_gene)
PTPN11 (cancer_gene)
Cluster3: 3 muts
KMT2A (cancer_gene)
Cluster4: 1 muts
PIK3R1 (cancer_gene)
Cluster5: 3 muts
IFNA1 (double_hit)
MUC17 (cancer_gene)
SLC22A23 (double_hit)
Cluster6: 23 muts
CD74 (cancer_gene)
KMT2D (cancer_gene)
TP53 (cancer_gene, double_hit)
B
GL
FAM65B + 5 mut.
1
KMT2A + 2 mut.
OPCML, PTPN11, GNAS + 10 mut.
2
3
PIK3R1
5
6
4

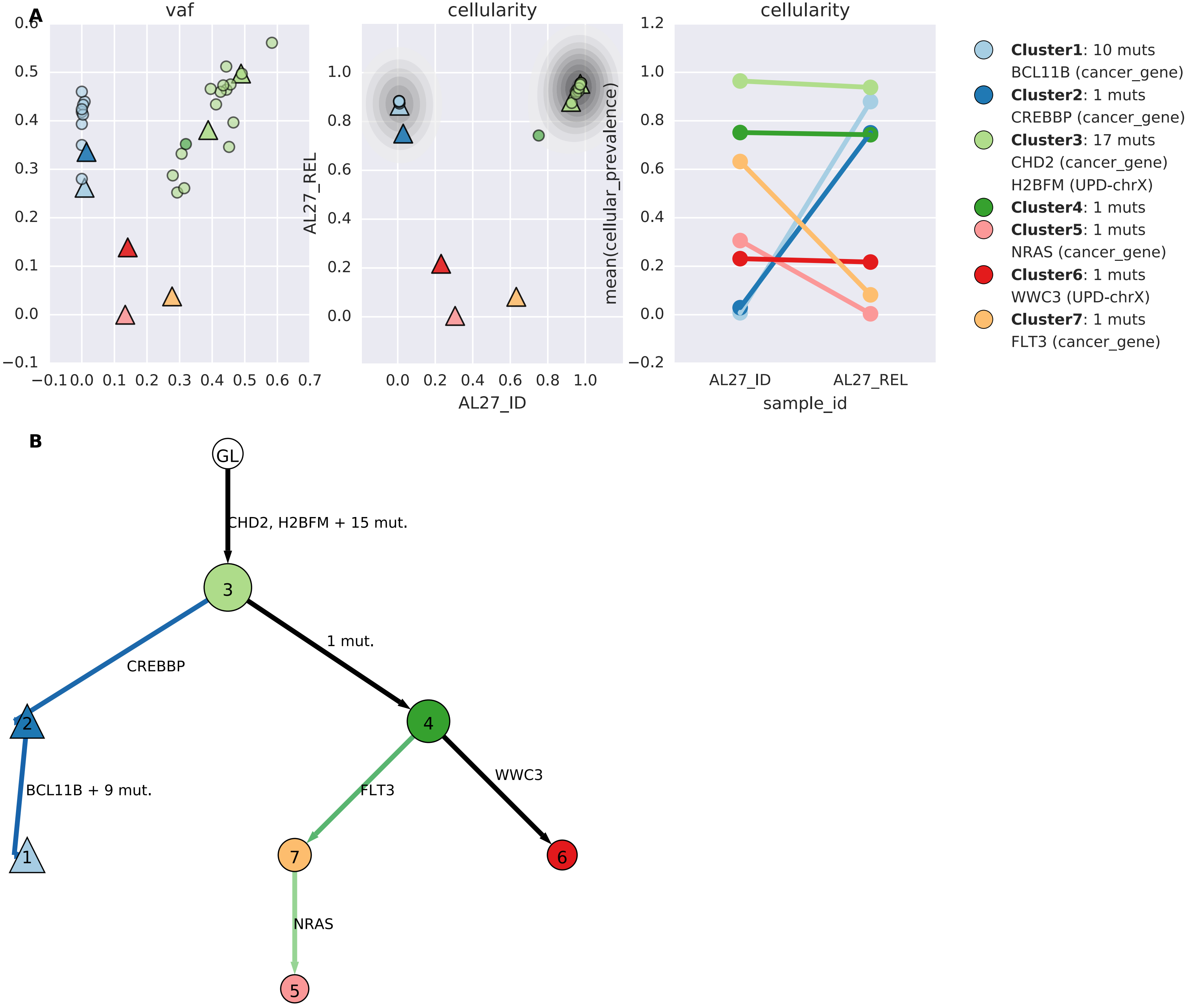
A
vaf
cellularity
cellularity
AL27_REL
AL27_ID
mean(cellular_prevalence)
sample_id
AL27_ID
AL27_REL
Cluster1: 10 muts
BCL11B (cancer_gene)
Cluster2: 1 muts
CREBBP (cancer_gene)
Cluster3: 17 muts
CHD2 (cancer_gene)
H2BFM (UPD-chrX)
Cluster4: 1 muts
Cluster5: 1 muts
NRAS (cancer_gene)
Cluster6: 1 muts
WWC3 (UPD-chrX)
Cluster7: 1 muts
FLT3 (cancer_gene)
B
GL
CHD2, H2BFM + 15 mut.
3
CREBBP
1 mut.
2
4
BCL11B + 9 mut.
FLT3
WWC3
1
7
6
NRAS
5

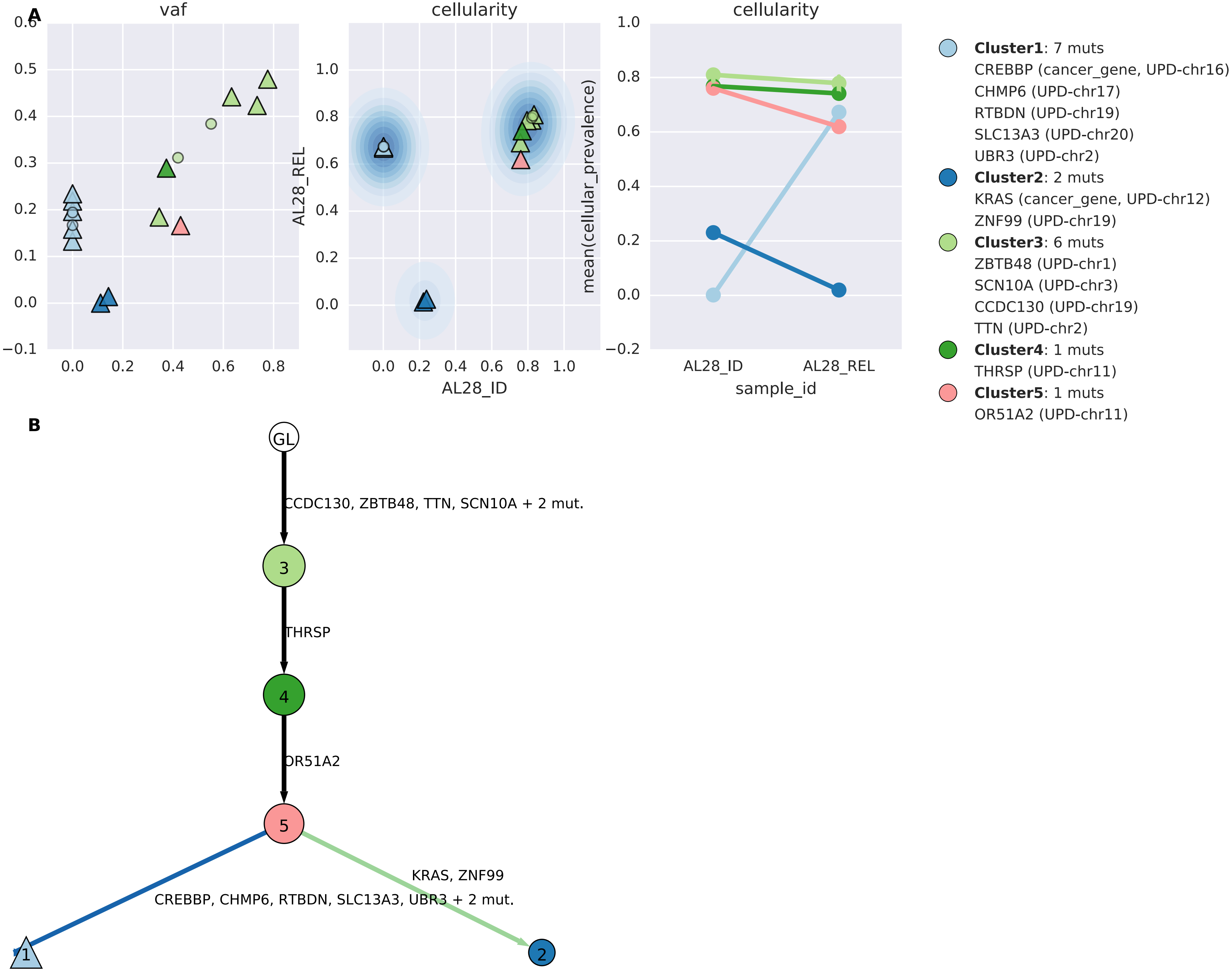

A
vaf
cellularity
AL28_REL
AL28_ID
cellularity
mean(cellular_prevalence)
AL28_ID
AL28_REL
sample_id
Cluster1: 7 muts
CREBBP (cancer_gene, UPD-chr16)
CHMP6 (UPD-chr17)
RTBDN (UPD-chr19)
SLC13A3 (UPD-chr20)
UBR3 (UPD-chr2)
Cluster2: 2 muts
KRAS (cancer_gene, UPD-chr12)
ZNF99 (UPD-chr19)
Cluster3: 6 muts
ZBTB48 (UPD-chr1)
SCN10A (UPD-chr3)
CCDC130 (UPD-chr19)
TTN (UPD-chr2)
Cluster4: 1 muts
THRSP (UPD-chr11)
Cluster5: 1 muts
OR51A2 (UPD-chr11)
B
GL
CCDC130, ZBTB48, TTN, SCN10A + 2 mut.
3
THRSP
4
OR51A2
5
KRAS, ZNF99
CREBBP, CHMP6, RTBDN, SLC13A3, UBR3 + 2 mut.
1
2

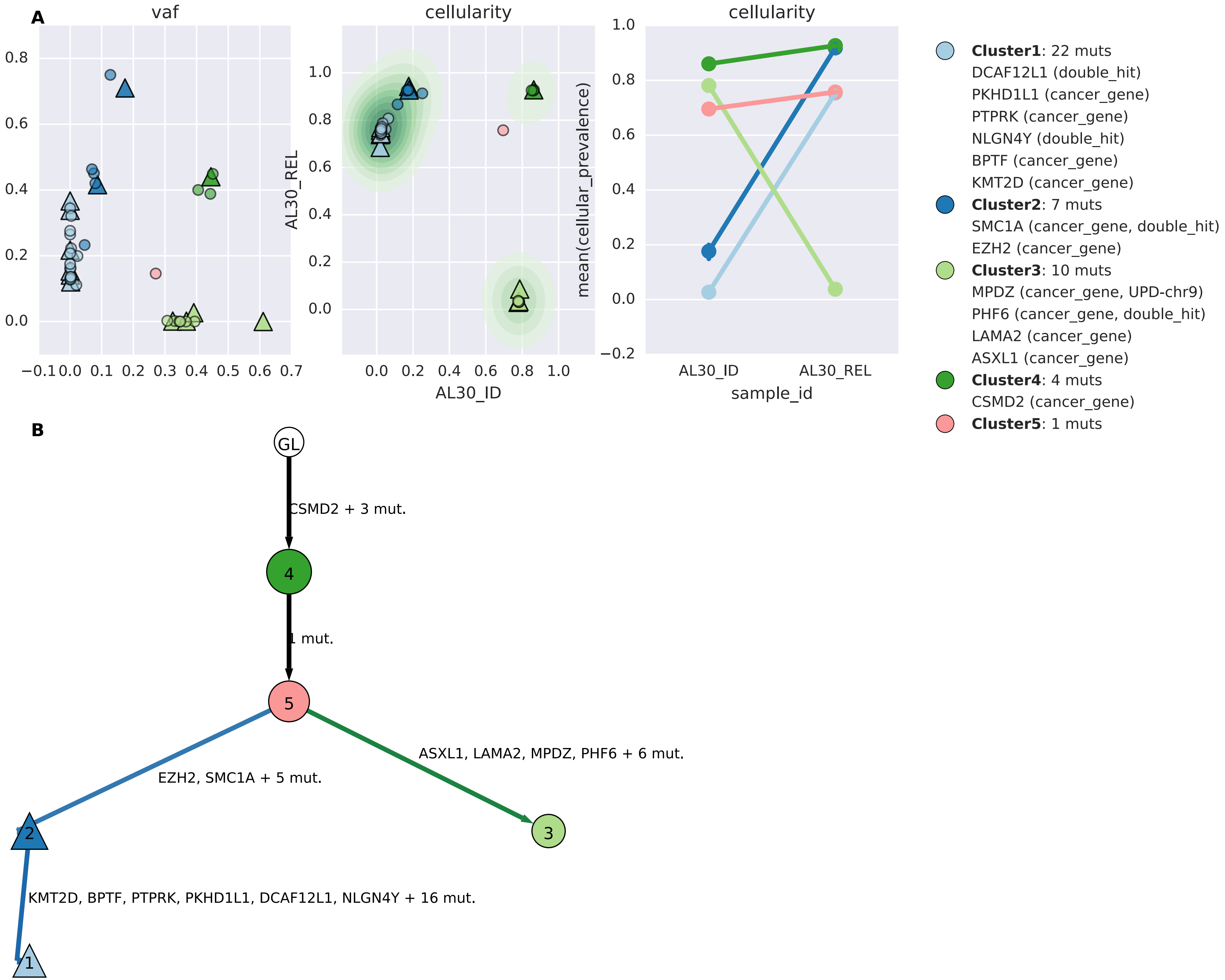
A
vaf
cellularity
AL30_REL
AL30_ID
cellularity
mean(cellular_prevalence)
AL30_ID
AL30_REL
sample_id
Cluster1: 22 muts
DCAF12L1 (double_hit)
PKHD1L1 (cancer_gene)
PTPRK (cancer_gene)
NLGN4Y (double_hit)
BPTF (cancer_gene)
KMT2D (cancer_gene)
Cluster2: 7 muts
SMC1A (cancer_gene, double_hit)
EZH2 (cancer_gene)
Cluster3: 10 muts
MPDZ (cancer_gene, UPD-chr9)
PHF6 (cancer_gene, double_hit)
LAMA2 (cancer_gene)
ASXL1 (cancer_gene)
Cluster4: 4 muts
CSMD2 (cancer_gene)
Cluster5: 1 muts
B
GL
CSMD2 + 3 mut.
4
1 mut.
5
EZH2, SMC1A + 5 mut.
ASXL1, LAMA2, MPDZ, PHF6 + 6 mut.
2
3
KMT2D, BPTF, PTPRK, PKHD1L1, DCAF12L1, NLGN4Y + 16 mut.
1

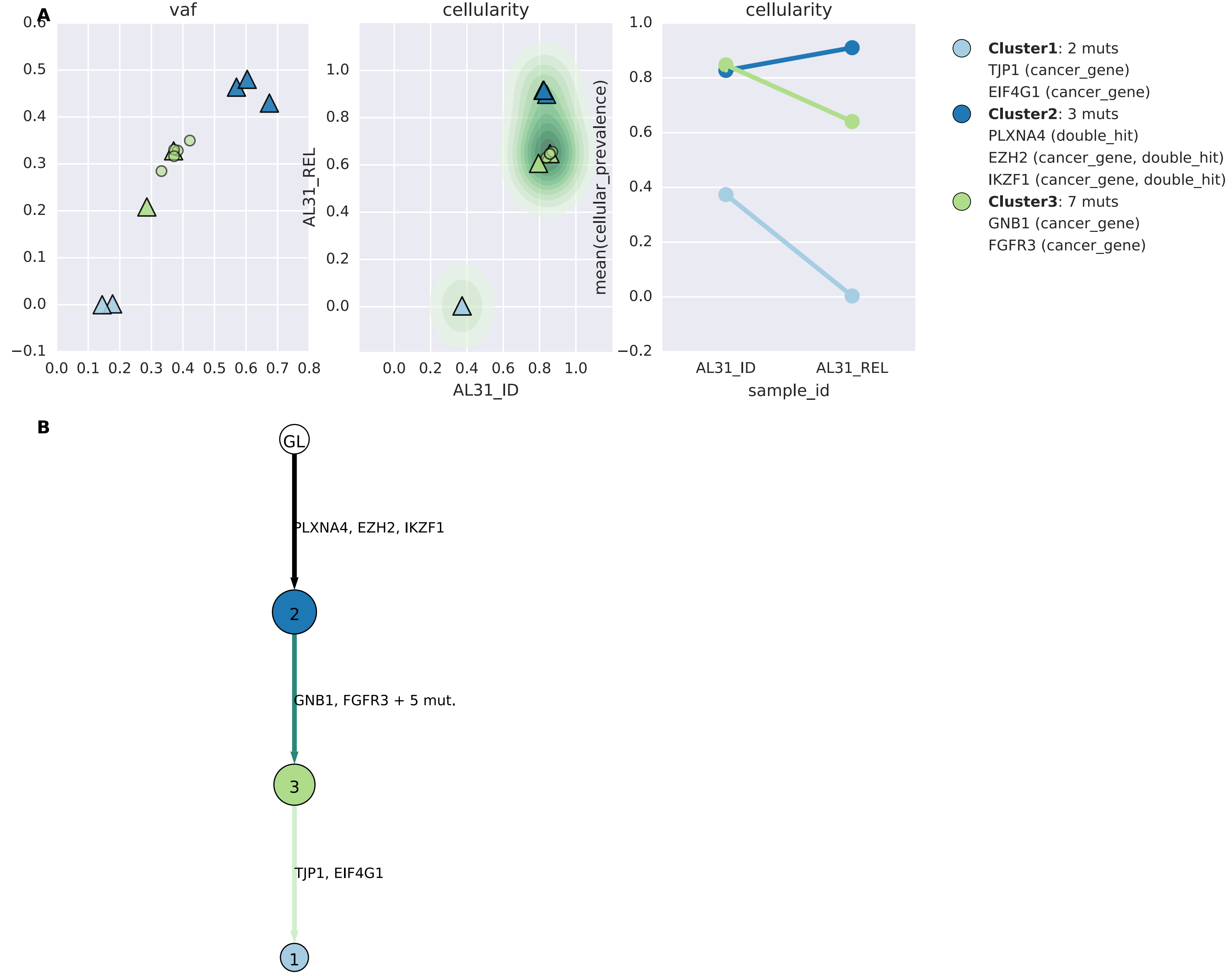
A
vaf
cellularity
AL31_REL
AL31_ID
cellularity
mean(cellular_prevalence)
AL31_ID
AL31_REL
sample_id
Cluster1: 2 muts
TJP1 (cancer_gene)
EIF4G1 (cancer_gene)
Cluster2: 3 muts
PLXNA4 (double_hit)
EZH2 (cancer_gene, double_hit)
IKZF1 (cancer_gene, double_hit)
Cluster3: 7 muts
GNB1 (cancer_gene)
FGFR3 (cancer_gene)
B
GL
PLXNA4, EZH2, IKZF1
2
GNB1, FGFR3 + 5 mut.
3
TJP1, EIF4G1
1

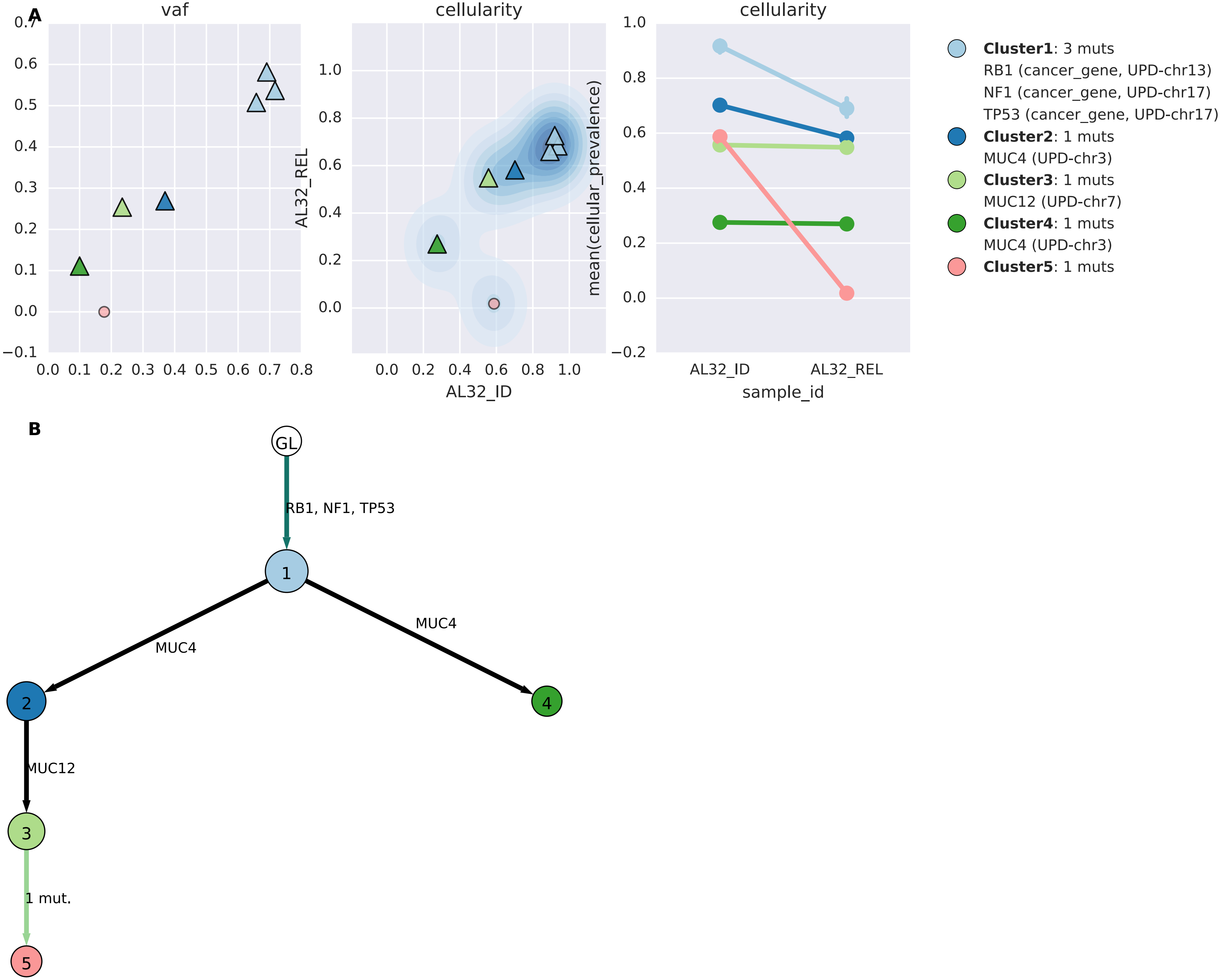
A
vaf
cellularity
AL32_REL
AL32_ID
cellularity
mean(cellular_prevalence)
AL32_ID
AL32_REL
sample_id
Cluster1: 3 muts
RB1 (cancer_gene, UPD-chr13)
NF1 (cancer_gene, UPD-chr17)
TP53 (cancer_gene, UPD-chr17)
Cluster2: 1 muts
MUC4 (UPD-chr3)
Cluster3: 1 muts
MUC12 (UPD-chr7)
Cluster4: 1 muts
MUC4 (UPD-chr3)
Cluster5: 1 muts
B
GL
RB1, NF1, TP53
1
MUC4
MUC4
2
4
MUC12
3
1 mut.
5

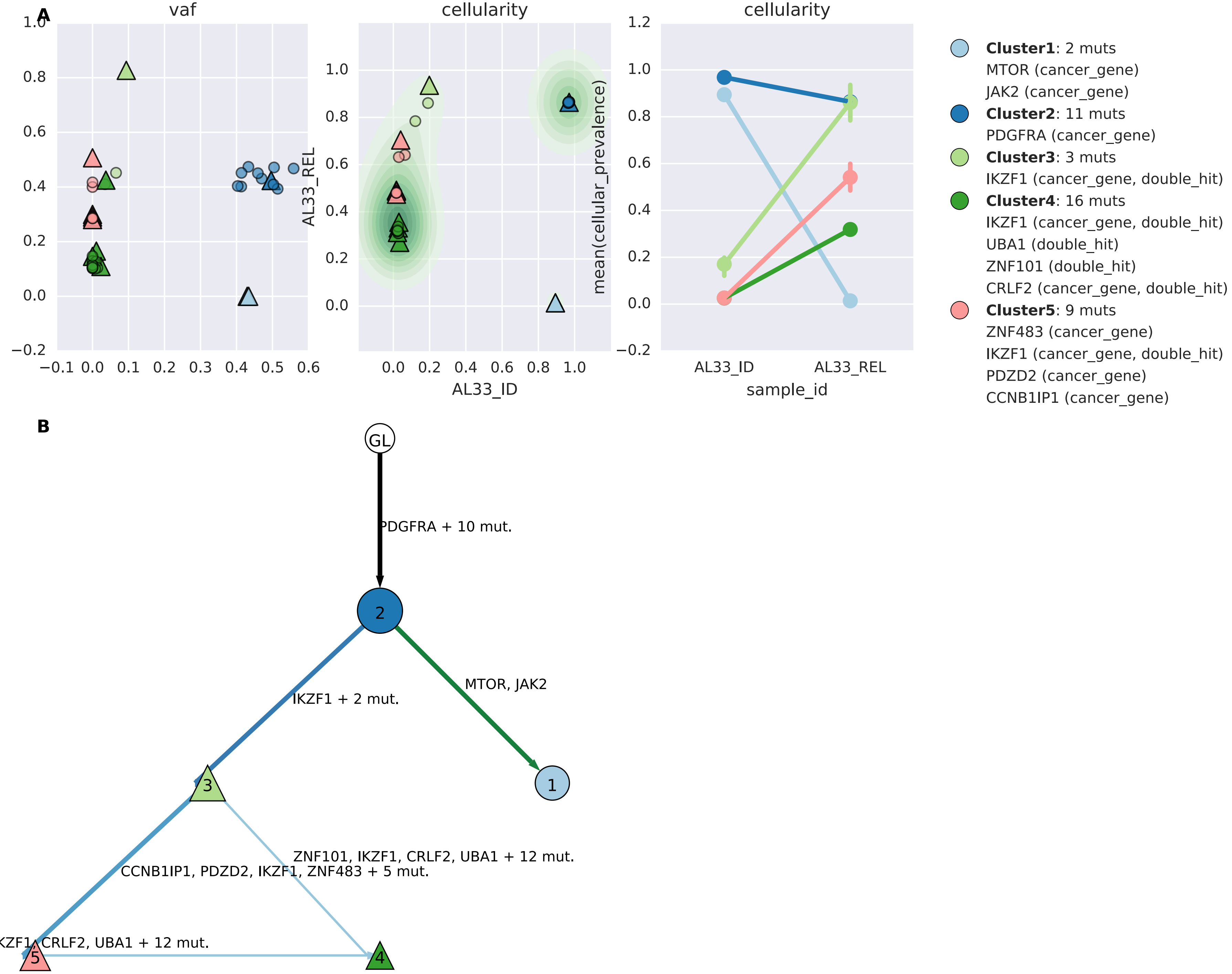
A
vaf
cellularity
cellularity
AL33_REL
AL33_ID
mean(cellular_prevalence)
sample_id
AL33_ID
AL33_REL
Cluster1: 2 muts
MTOR (cancer_gene)
JAK2 (cancer_gene)
Cluster2: 11 muts
PDGFRA (cancer_gene)
Cluster3: 3 muts
IKZF1 (cancer_gene, double_hit)
Cluster4: 16 muts
IKZF1 (cancer_gene, double_hit)
UBA1 (double_hit)
ZNF101 (double_hit)
CRLF2 (cancer_gene, double_hit)
Cluster5: 9 muts
ZNF483 (cancer_gene)
IKZF1 (cancer_gene, double_hit)
PDZD2 (cancer_gene)
CCNB1IP1 (cancer_gene)
B
GL
PDGFRA + 10 mut.
2
IKZF1 + 2 mut.
MTOR, JAK2
3
1
ZNF101, IKZF1, CRLF2, UBA1 + 12 mut.
CCNB1IP1, PDZD2, IKZF1, ZNF483 + 5 mut.
KZF1, CRLF2, UBA1 + 12 mut.
5
4

A

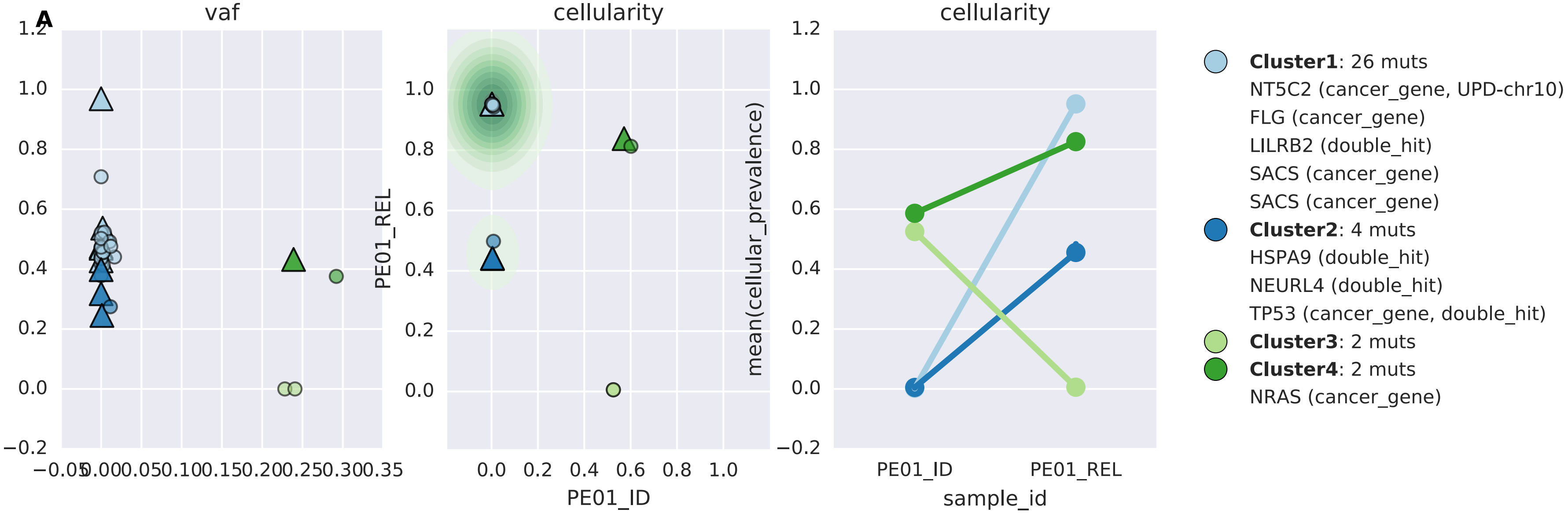
vaf
cellularity
cellularity
PE01_REL
PE01_ID
mean(cellular_prevalence)
sample_id
PE01_ID
PE01_REL
Cluster1: 26 muts
NT5C2 (cancer_gene, UPD-chr10)
FLG (cancer_gene)
LILRB2 (double_hit)
SACS (cancer_gene)
SACS (cancer_gene)
Cluster2: 4 muts
HSPA9 (double_hit)
NEURL4 (double_hit)
TP53 (cancer_gene, double_hit)
Cluster3: 2 muts
Cluster4: 2 muts
NRAS (cancer_gene)

B

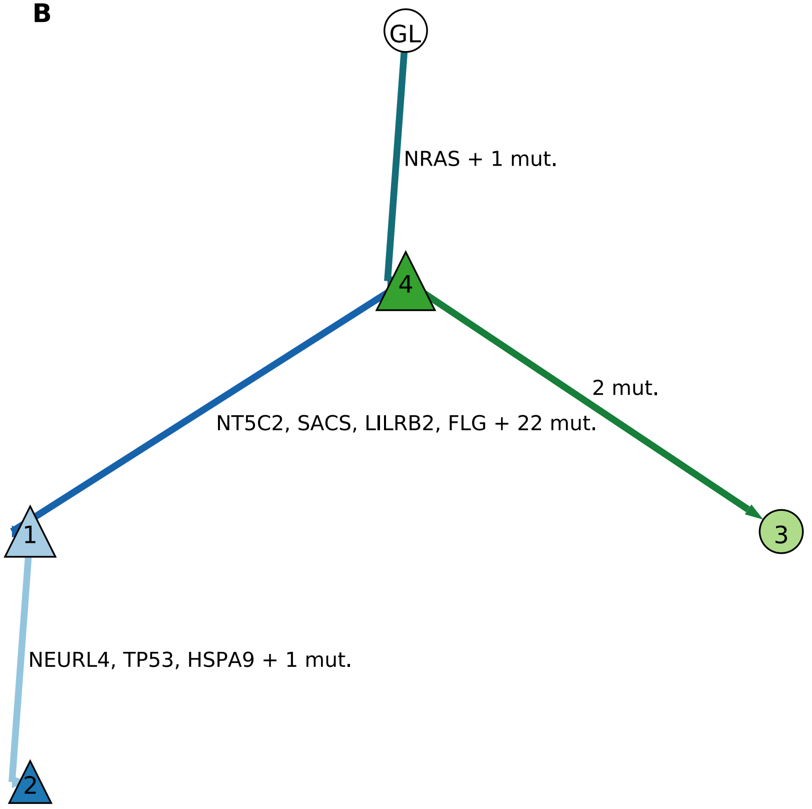
GL
NRAS + 1 mut.
4
2 mut.
NT5C2, SACS, LILRB2, FLG + 22 mut.
1
3
NEURL4, TP53, HSPA9 + 1 mut.
2

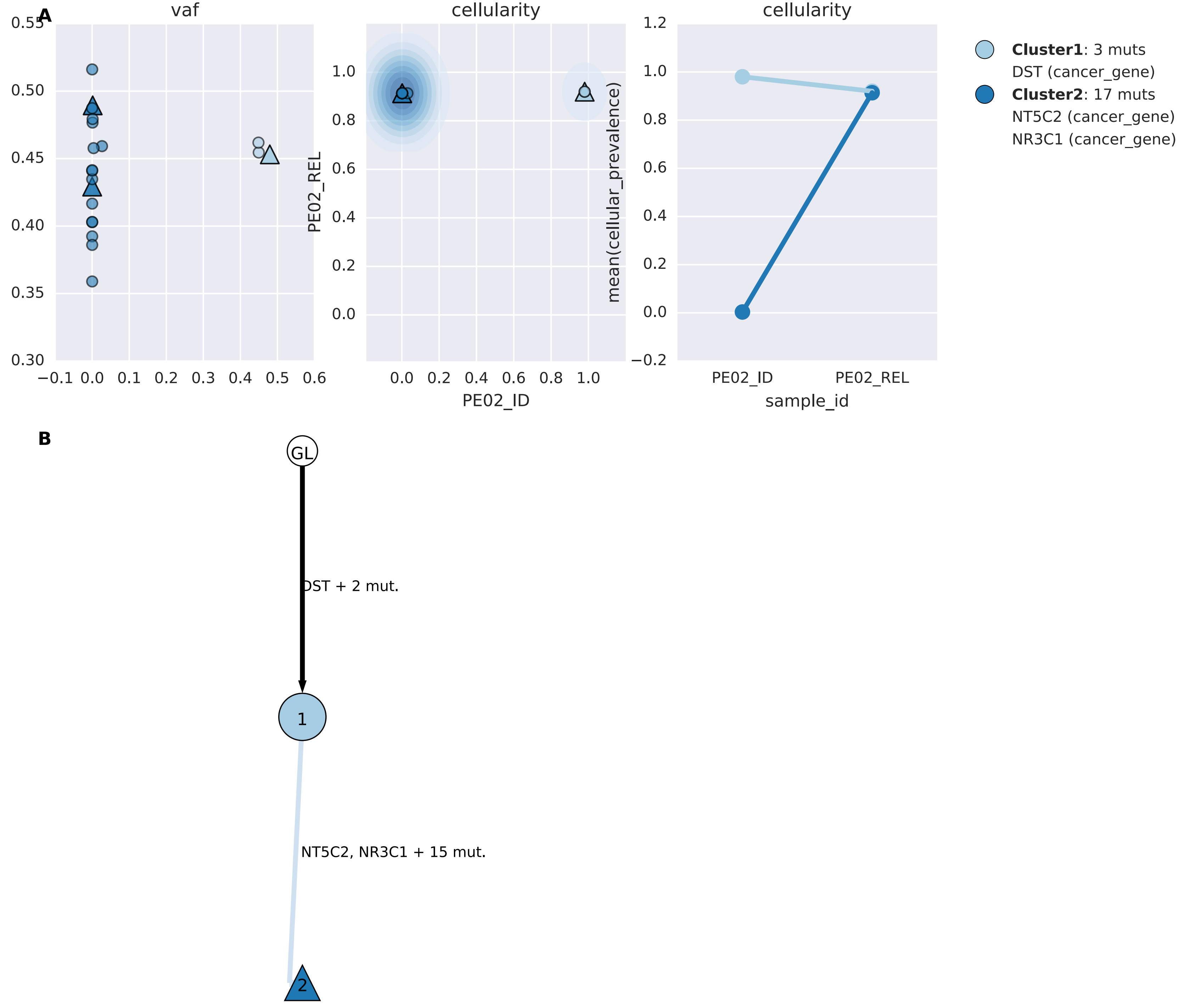
A
vaf
0.55
0.50
0.45
0.40
0.35
0.30
−0.1 0.0 0.1 0.2 0.3 0.4 0.5 0.6
cellularity
PE02_REL
1.0
0.8
0.6
0.4
0.2
0.0
0.0 0.2 0.4 0.6 0.8 1.0
PE02_ID
cellularity
mean(cellular_prevalence)
1.2
1.0
0.8
0.6
0.4
0.2
0.0
−0.2
PE02_ID
PE02_REL
sample_id
Cluster1: 3 muts
DST (cancer_gene)
Cluster2: 17 muts
NT5C2 (cancer_gene)
NR3C1 (cancer_gene)
B
GL
DST + 2 mut.
1
NT5C2, NR3C1 + 15 mut.
2

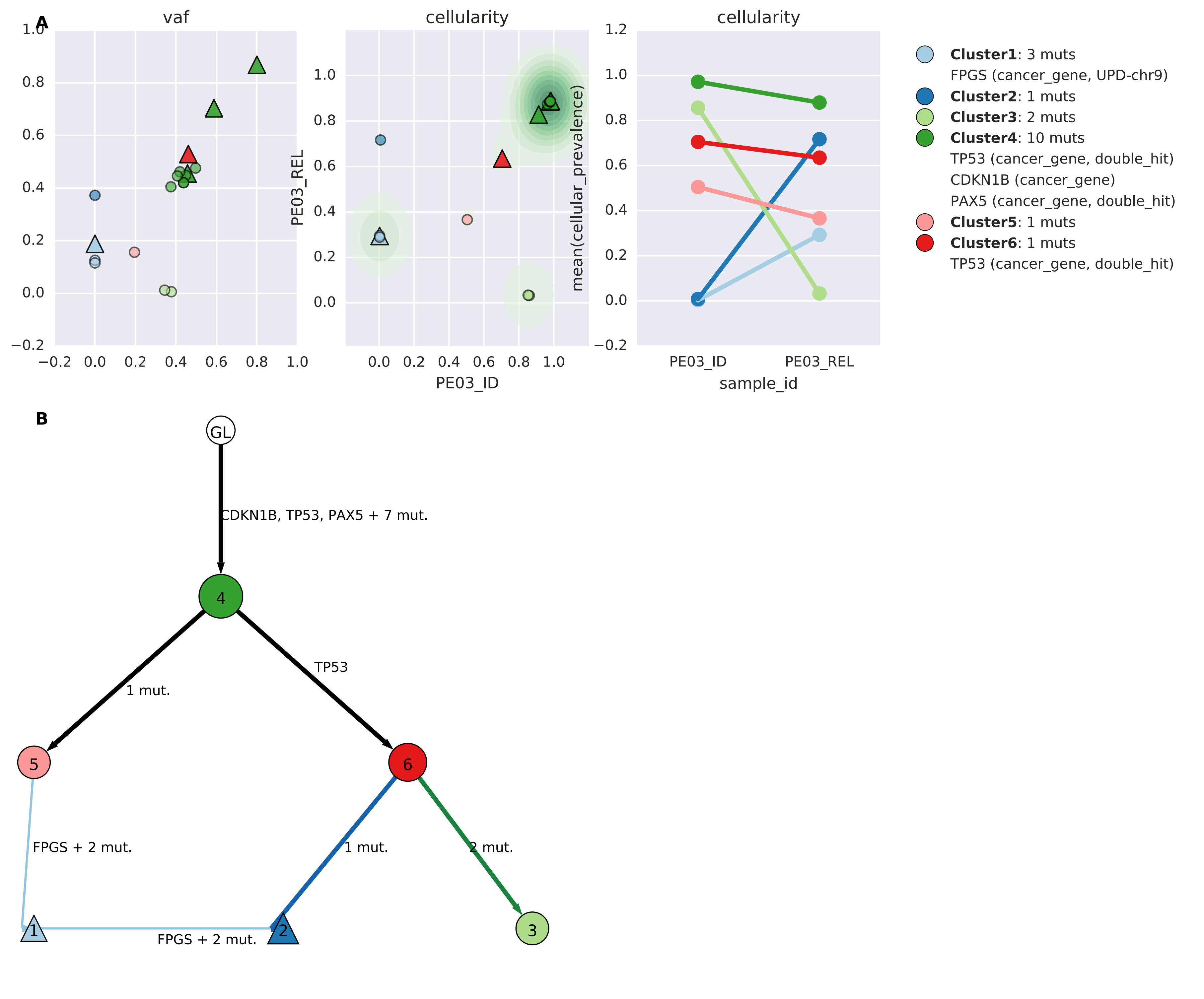
A
vaf
cellularity
PE03_REL
PE03_ID
cellularity
mean(cellular_prevalence)
PE03_ID
PE03_REL
sample_id
Cluster1: 3 muts
FPGS (cancer_gene, UPD-chr9)
Cluster2: 1 muts
Cluster3: 2 muts
Cluster4: 10 muts
TP53 (cancer_gene, double_hit)
CDKN1B (cancer_gene)
PAX5 (cancer_gene, double_hit)
Cluster5: 1 muts
Cluster6: 1 muts
TP53 (cancer_gene, double_hit)
B
GL
CDKN1B, TP53, PAX5 + 7 mut.
4
1 mut.
TP53
5
6
FPGS + 2 mut.
1 mut.
2 mut.
1
2
3
FPGS + 2 mut.

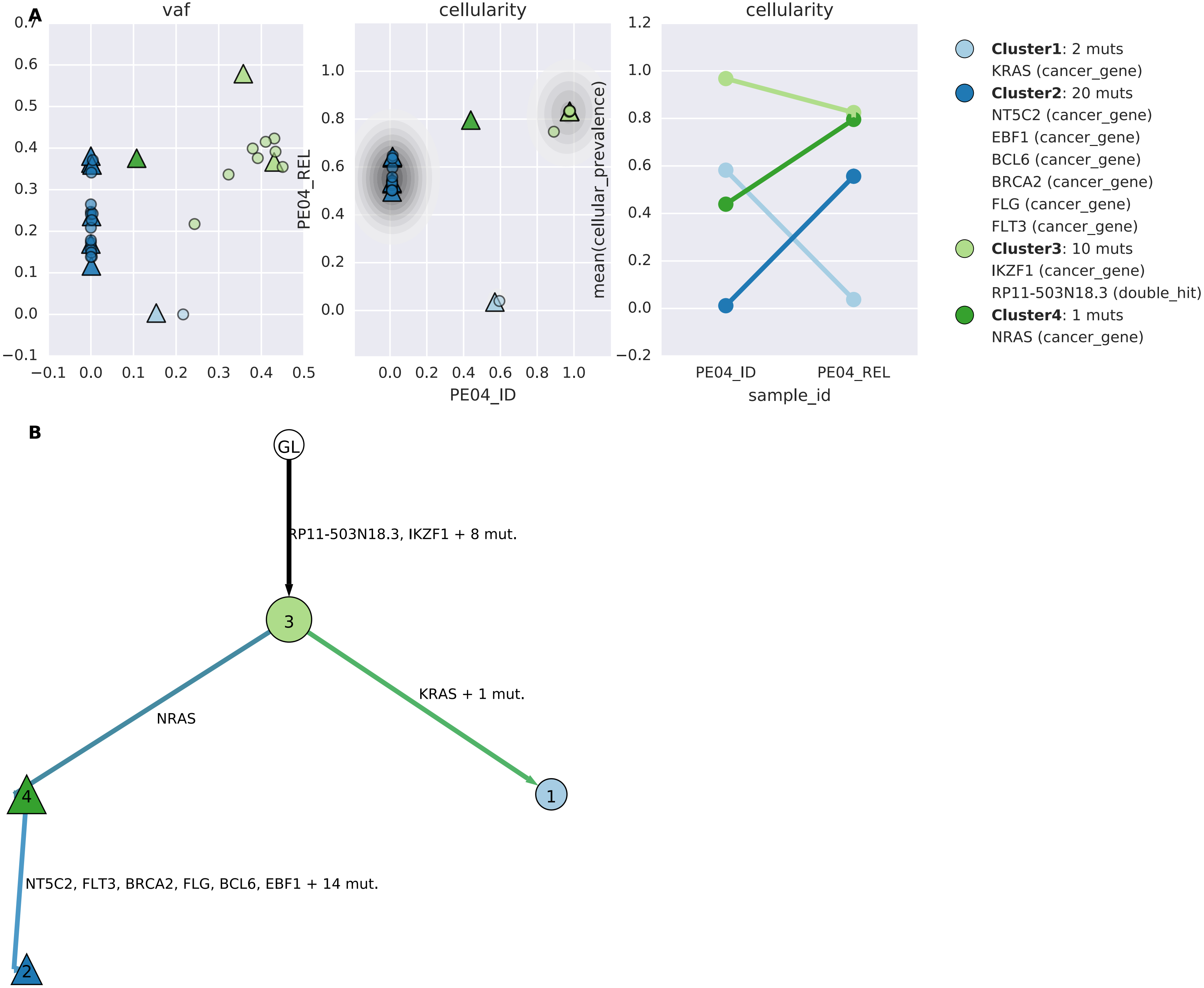
A
vaf
cellularity
cellularity
PE04_REL
PE04_ID
mean(cellular_prevalence)
sample_id
PE04_ID
PE04_REL
Cluster1: 2 muts
KRAS (cancer_gene)
Cluster2: 20 muts
NT5C2 (cancer_gene)
EBF1 (cancer_gene)
BCL6 (cancer_gene)
BRCA2 (cancer_gene)
FLG (cancer_gene)
FLT3 (cancer_gene)
Cluster3: 10 muts
IKZF1 (cancer_gene)
RP11-503N18.3 (double_hit)
Cluster4: 1 muts
NRAS (cancer_gene)
B
GL
RP11-503N18.3, IKZF1 + 8 mut.
3
NRAS
KRAS + 1 mut.
4
1
NT5C2, FLT3, BRCA2, FLG, BCL6, EBF1 + 14 mut.
2

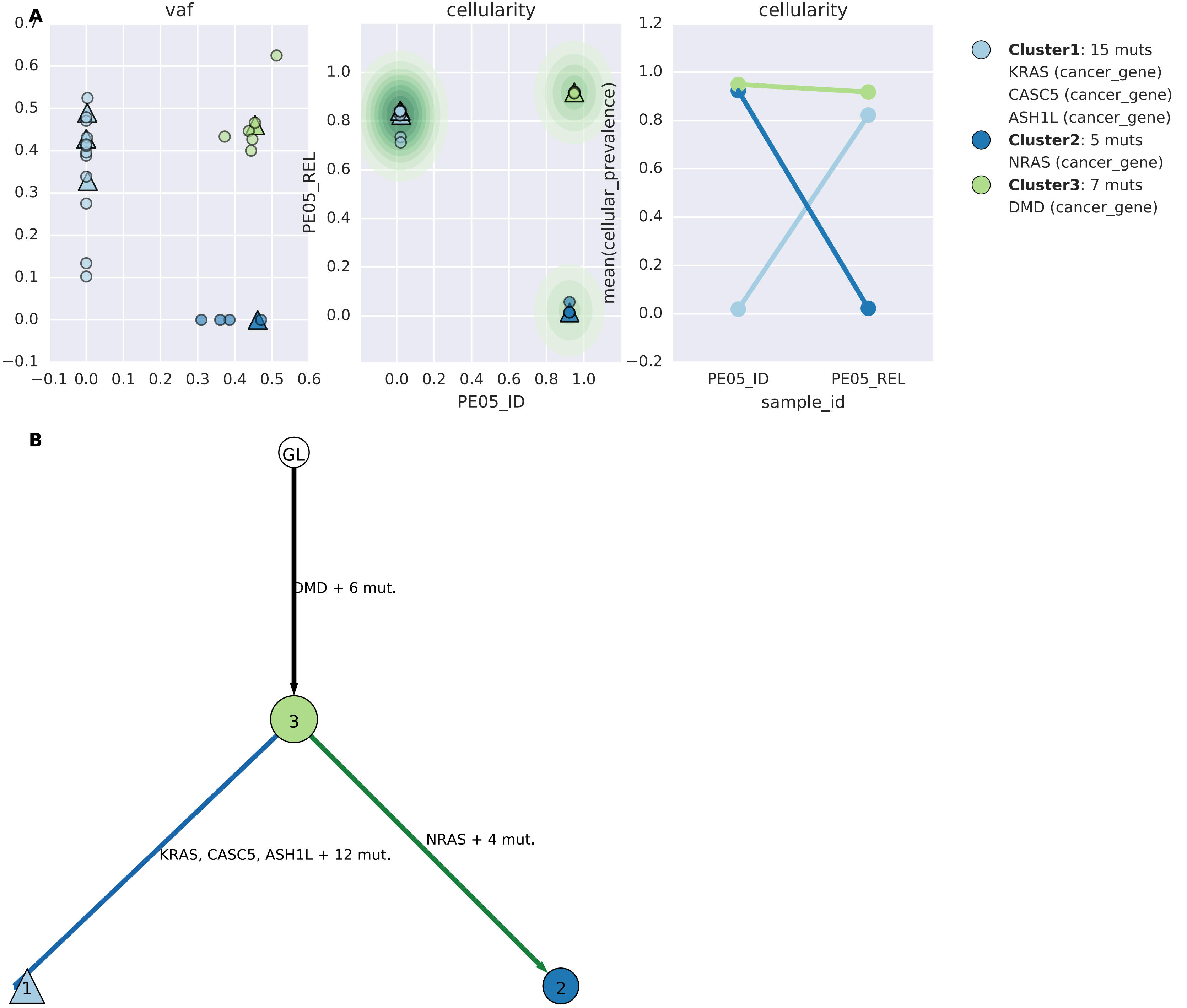
A
vaf
cellularity
PE05_REL
PE05_ID
cellularity
mean(cellular_prevalence)
sample_id
PE05_ID
PE05_REL
Cluster1: 15 muts
KRAS (cancer_gene)
CASC5 (cancer_gene)
ASH1L (cancer_gene)
Cluster2: 5 muts
NRAS (cancer_gene)
Cluster3: 7 muts
DMD (cancer_gene)
B
GL
DMD + 6 mut.
3
KRAS, CASC5, ASH1L + 12 mut.
NRAS + 4 mut.
1
2

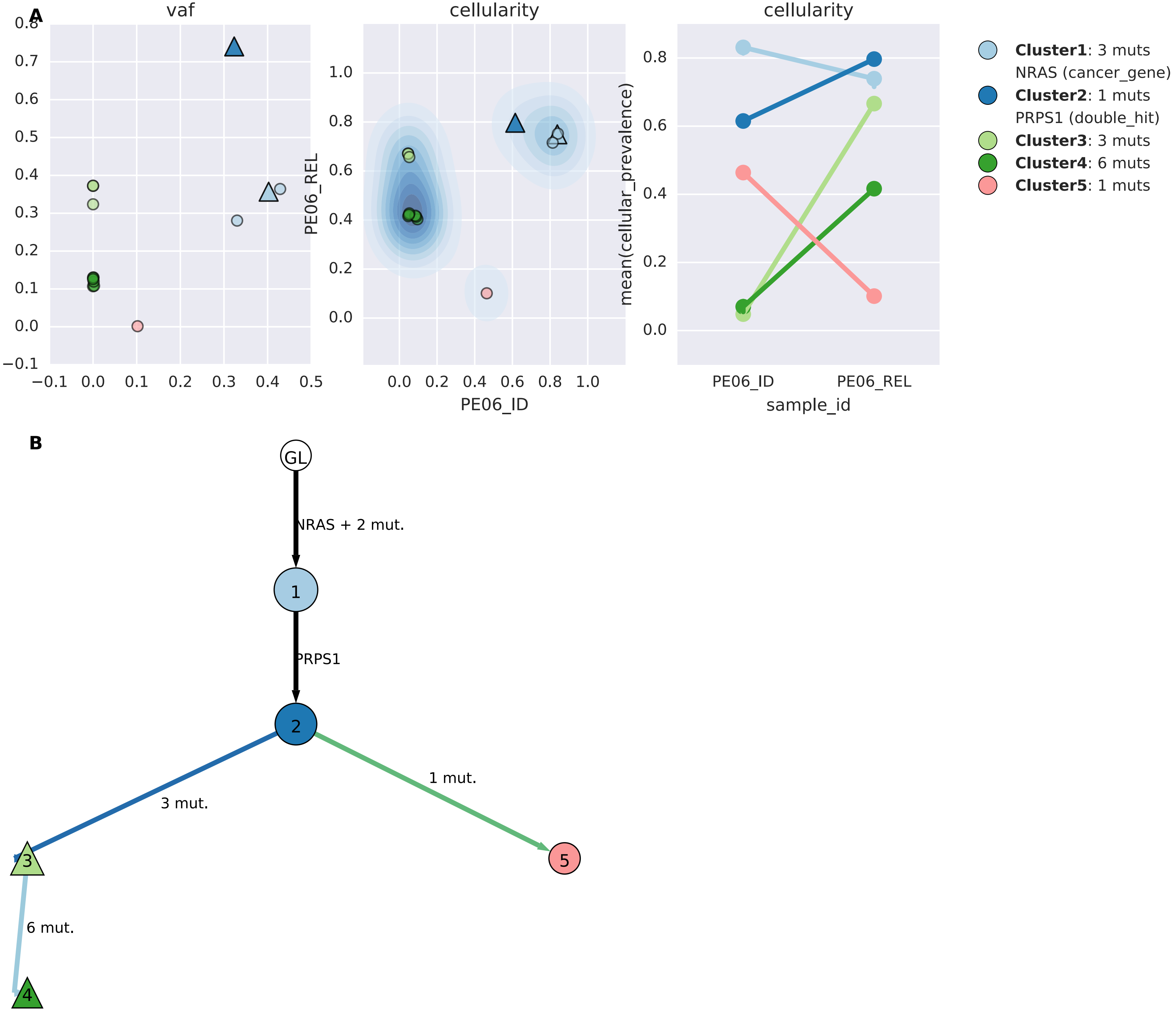
A
vaf
cellularity
PE06_REL
PE06_ID
cellularity
mean(cellular_prevalence)
PE06_ID
PE06_REL
sample_id
Cluster1: 3 muts
NRAS (cancer_gene)
Cluster2: 1 muts
PRPS1 (double_hit)
Cluster3: 3 muts
Cluster4: 6 muts
Cluster5: 1 muts
B
GL
NRAS + 2 mut.
1
PRPS1
2
3 mut.
1 mut.
3
5
6 mut.
4

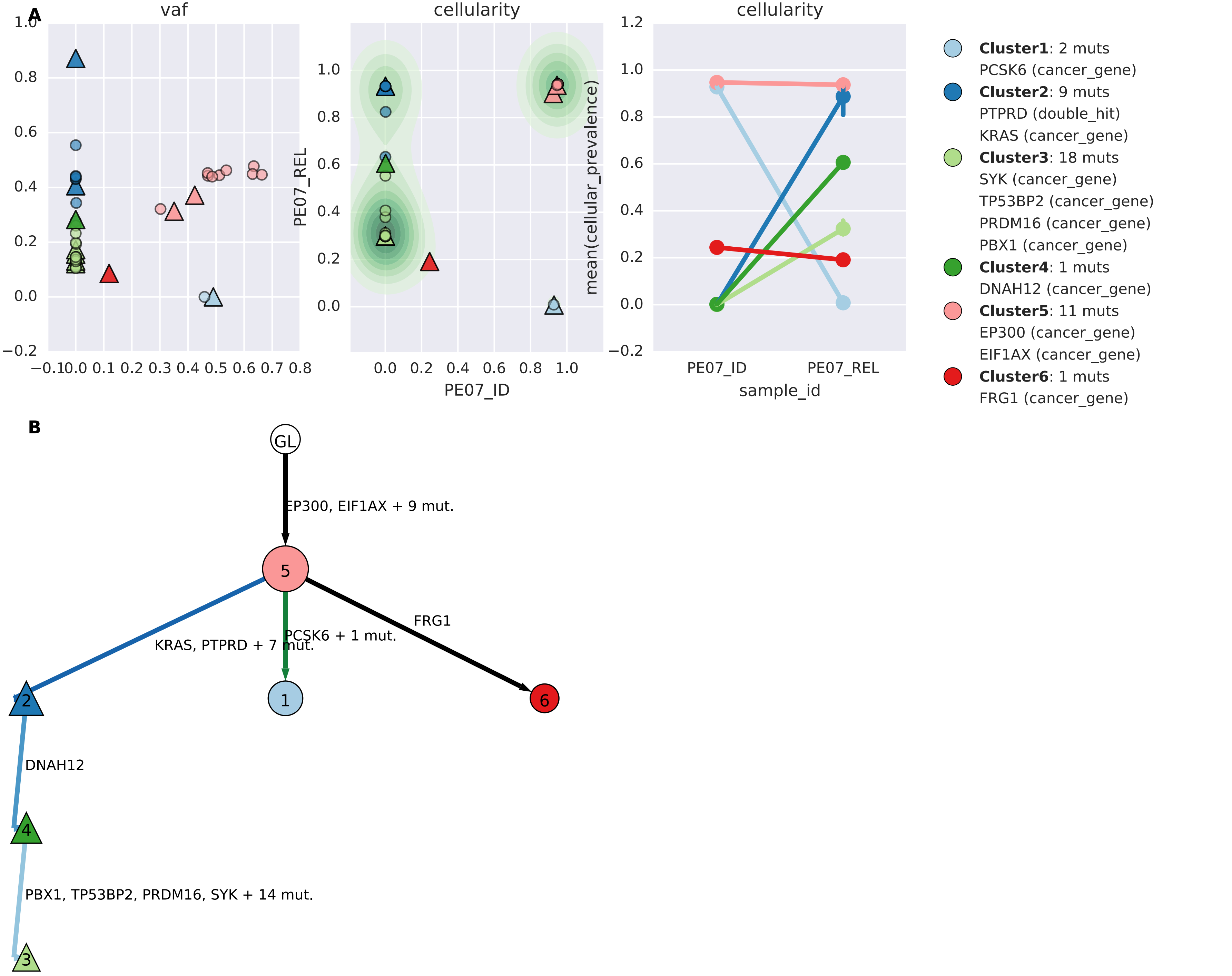
A
vaf
cellularity
PE07_REL
PE07_ID
cellularity
mean(cellular_prevalence)
sample_id
PE07_ID
PE07_REL
Cluster1: 2 muts
PCSK6 (cancer_gene)
Cluster2: 9 muts
PTPRD (double_hit)
KRAS (cancer_gene)
Cluster3: 18 muts
SYK (cancer_gene)
TP53BP2 (cancer_gene)
PRDM16 (cancer_gene)
PBX1 (cancer_gene)
Cluster4: 1 muts
DNAH12 (cancer_gene)
Cluster5: 11 muts
EP300 (cancer_gene)
EIF1AX (cancer_gene)
Cluster6: 1 muts
FRG1 (cancer_gene)
B
GL
EP300, EIF1AX + 9 mut.
5
KRAS, PTPRD + 7 mut.
PCSK6 + 1 mut.
FRG1
2
1
6
DNAH12
4
PBX1, TP53BP2, PRDM16, SYK + 14 mut.
3

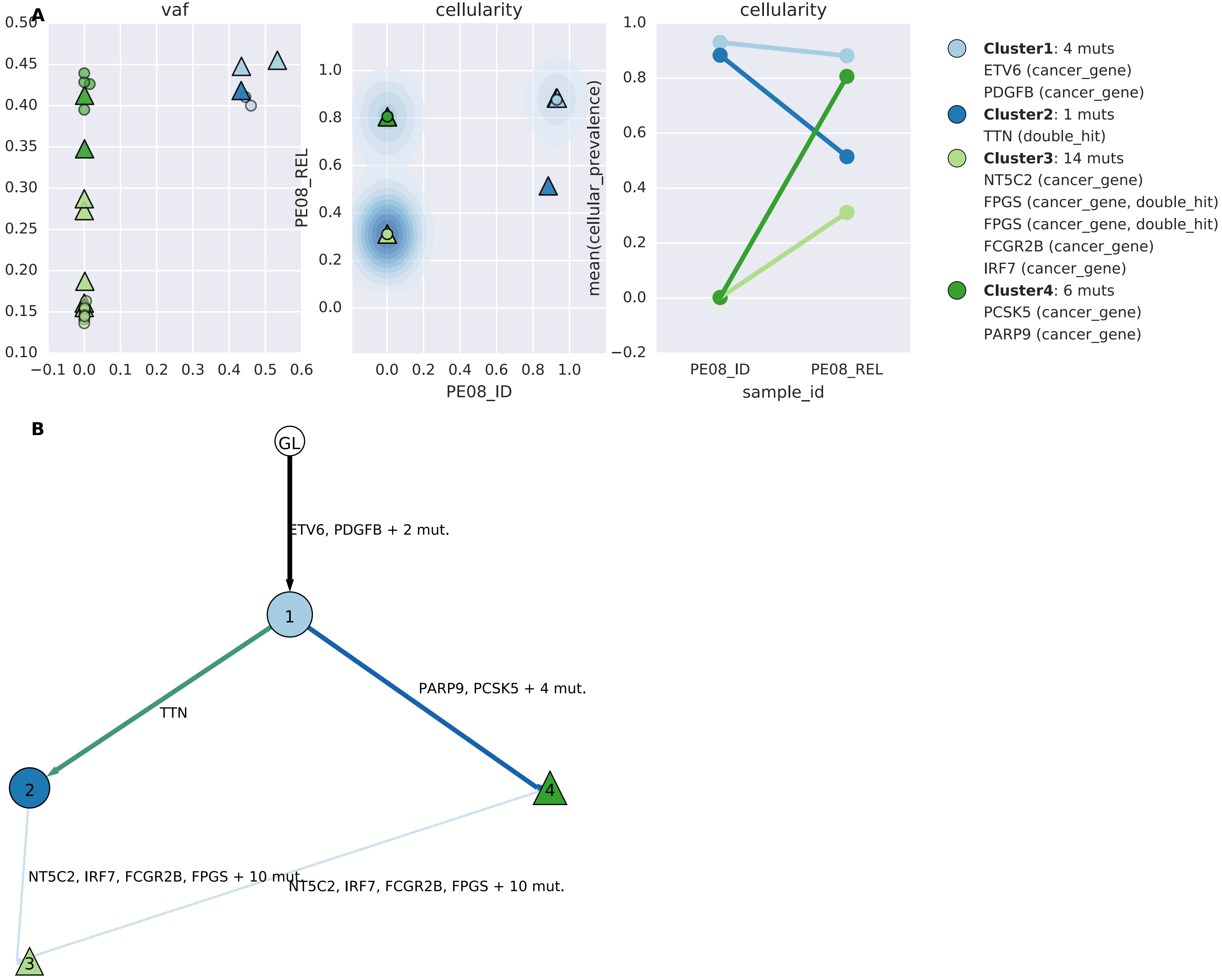
A
vaf
cellularity
PE08_REL
PE08_ID
cellularity
mean(cellular_prevalence)
PE08_ID
PE08_REL
sample_id
Cluster1: 4 muts
ETV6 (cancer_gene)
PDGFB (cancer_gene)
Cluster2: 1 muts
TTN (double_hit)
Cluster3: 14 muts
NT5C2 (cancer_gene)
FPGS (cancer_gene, double_hit)
FPGS (cancer_gene, double_hit)
FCGR2B (cancer_gene)
IRF7 (cancer_gene)
Cluster4: 6 muts
PCSK5 (cancer_gene)
PARP9 (cancer_gene)
B
GL
ETV6, PDGFB + 2 mut.
1
TTN
PARP9, PCSK5 + 4 mut.
2
4
NT5C2, IRF7, FCGR2B, FPGS + 10 mut.
NT5C2, IRF7, FCGR2B, FPGS + 10 mut.
3

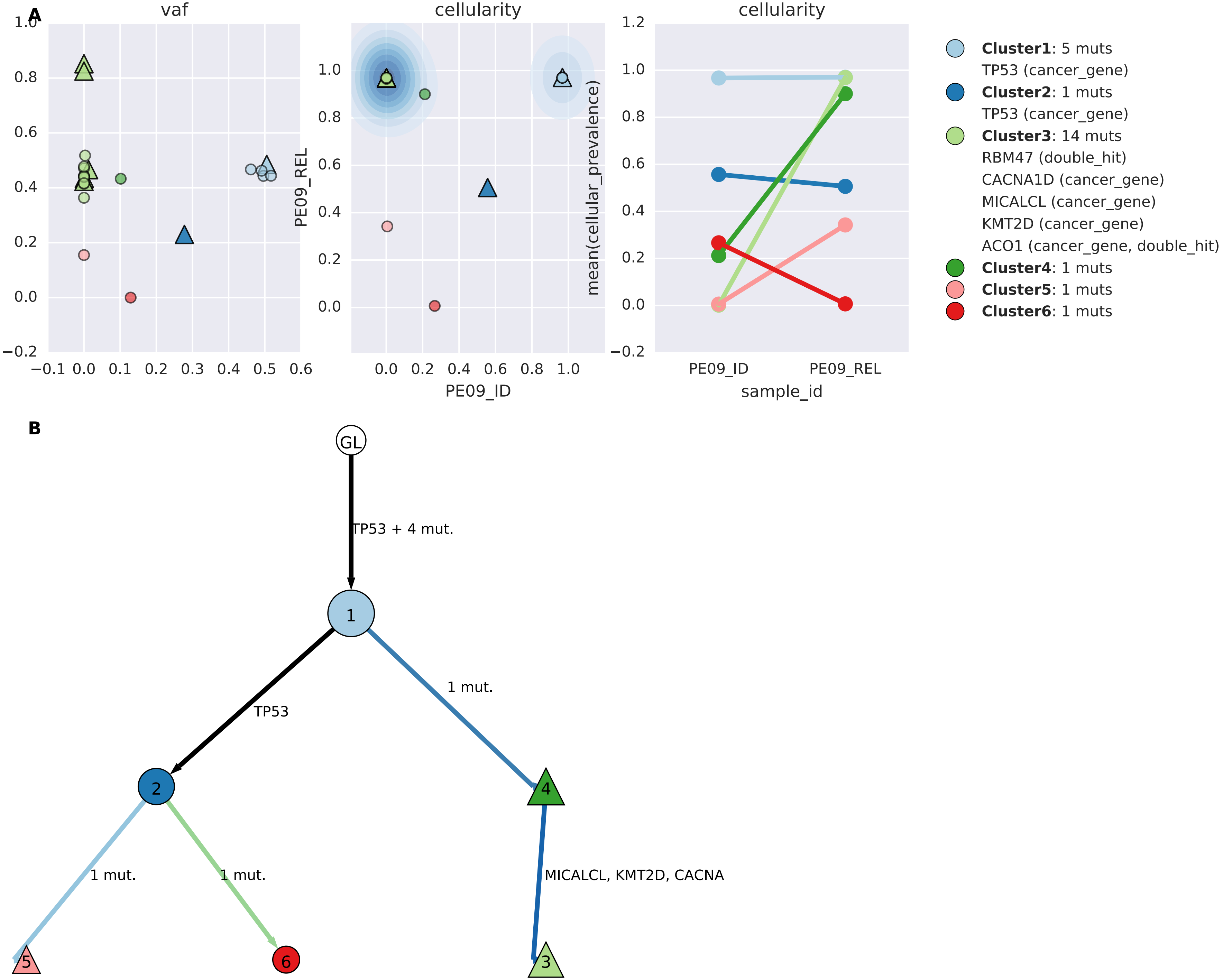

A
vaf
cellularity
PE09_REL
PE09_ID
cellularity
mean(cellular_prevalence)
sample_id
PE09_ID
PE09_REL
Cluster1: 5 muts
TP53 (cancer_gene)
Cluster2: 1 muts
TP53 (cancer_gene)
Cluster3: 14 muts
RBM47 (double_hit)
CACNA1D (cancer_gene)
MICALCL (cancer_gene)
KMT2D (cancer_gene)
ACO1 (cancer_gene, double_hit)
Cluster4: 1 muts
Cluster5: 1 muts
Cluster6: 1 muts
B
GL
TP53 + 4 mut.
1
TP53
1 mut.
2
4
1 mut.
1 mut.
MICALCL, KMT2D, CACNA
5
6
3

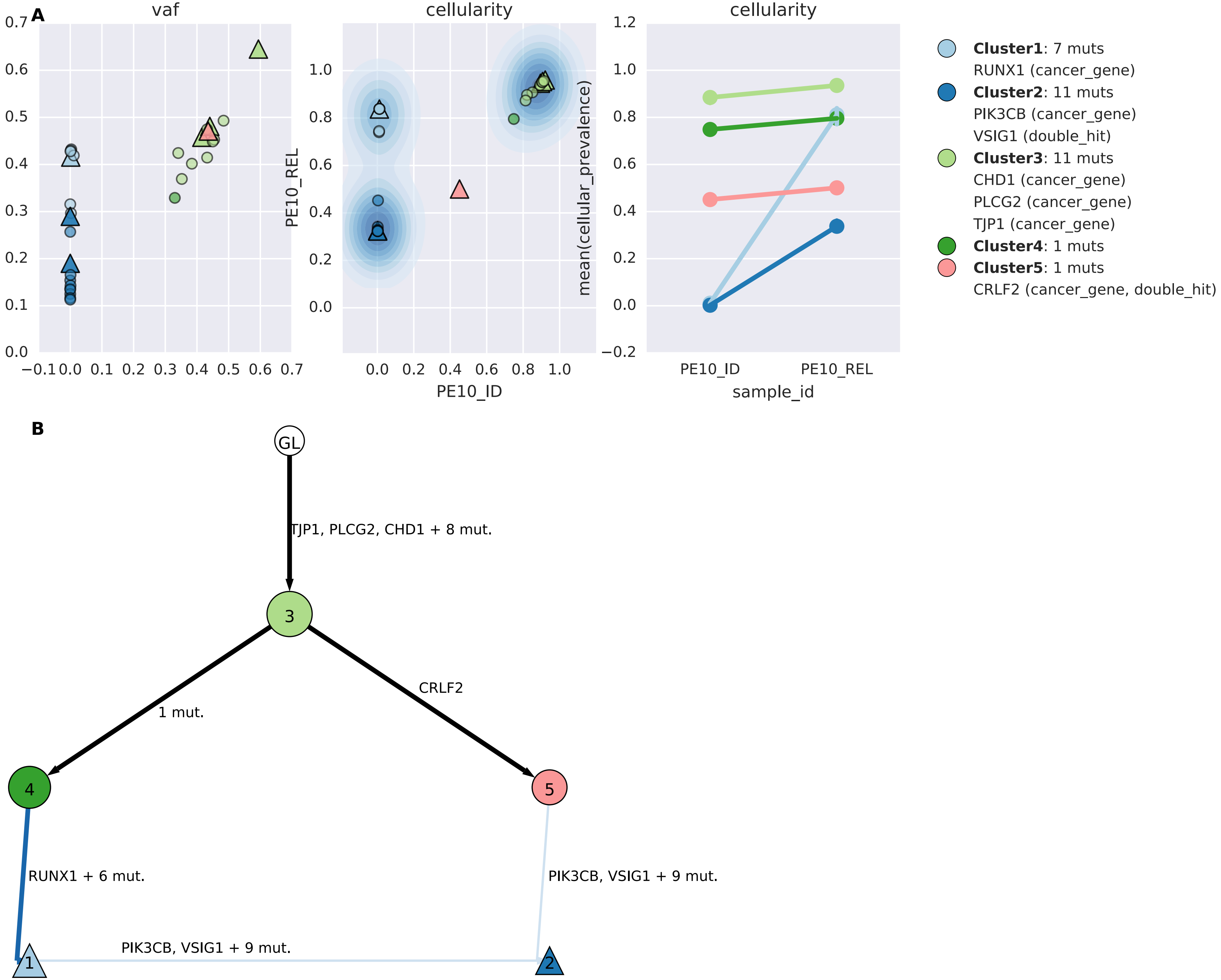

A
vaf
cellularity
PE10_REL
PE10_ID
cellularity
mean(cellular_prevalence)
sample_id
PE10_ID
PE10_REL
Cluster1: 7 muts
RUNX1 (cancer_gene)
Cluster2: 11 muts
PIK3CB (cancer_gene)
VSIG1 (double_hit)
Cluster3: 11 muts
CHD1 (cancer_gene)
PLCG2 (cancer_gene)
TJP1 (cancer_gene)
Cluster4: 1 muts
Cluster5: 1 muts
CRLF2 (cancer_gene, double_hit)
B
GL
TJP1, PLCG2, CHD1 + 8 mut.
3
1 mut.
CRLF2
4
5
RUNX1 + 6 mut.
PIK3CB, VSIG1 + 9 mut.
1
PIK3CB, VSIG1 + 9 mut.
2

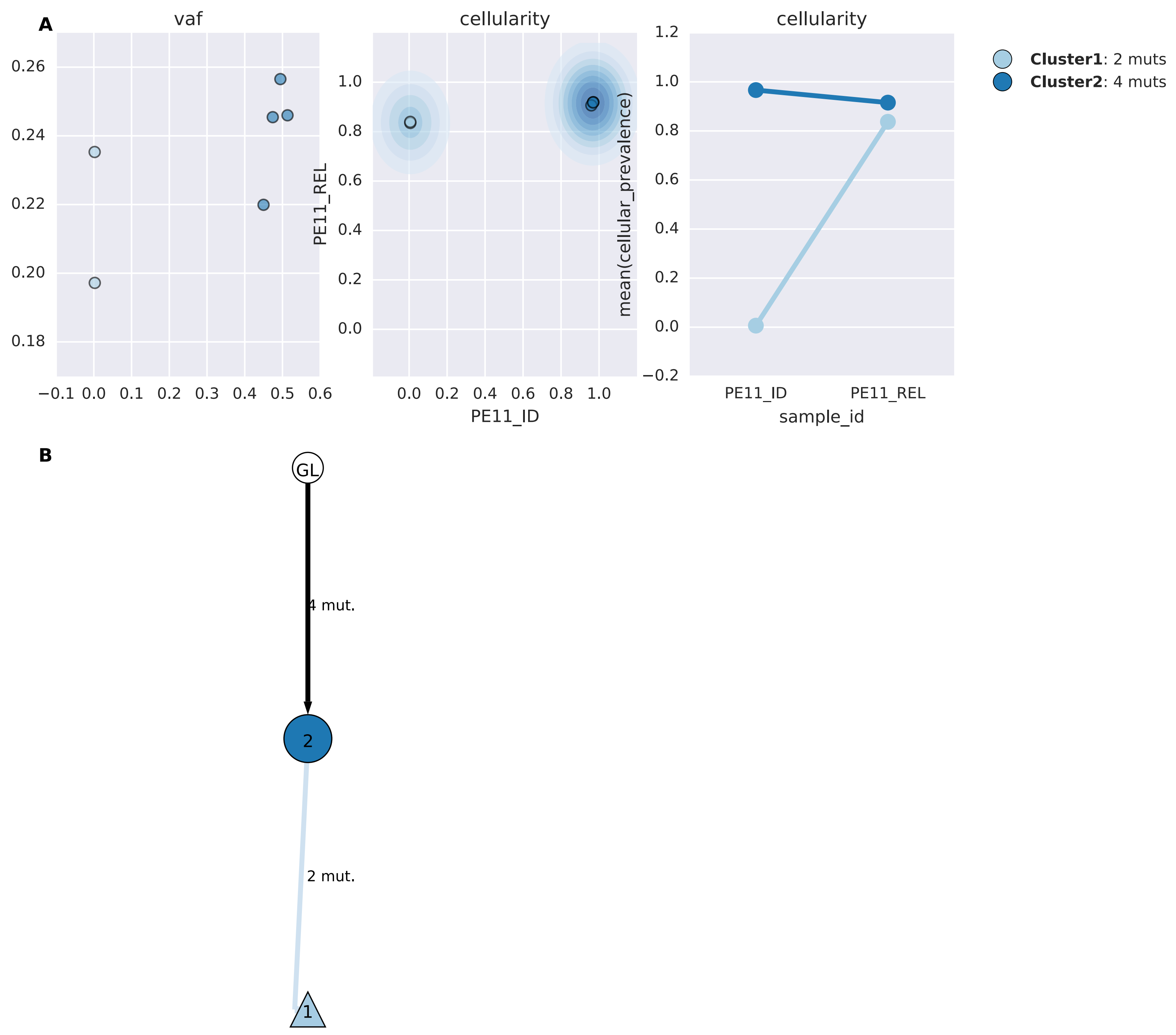
A
vaf
cellularity
PE11_REL
PE11_ID
cellularity
mean(cellular_prevalence)
PE11_ID
PE11_REL
sample_id
Cluster1: 2 muts
Cluster2: 4 muts
B
GL
4 mut.
2
2 mut.
1

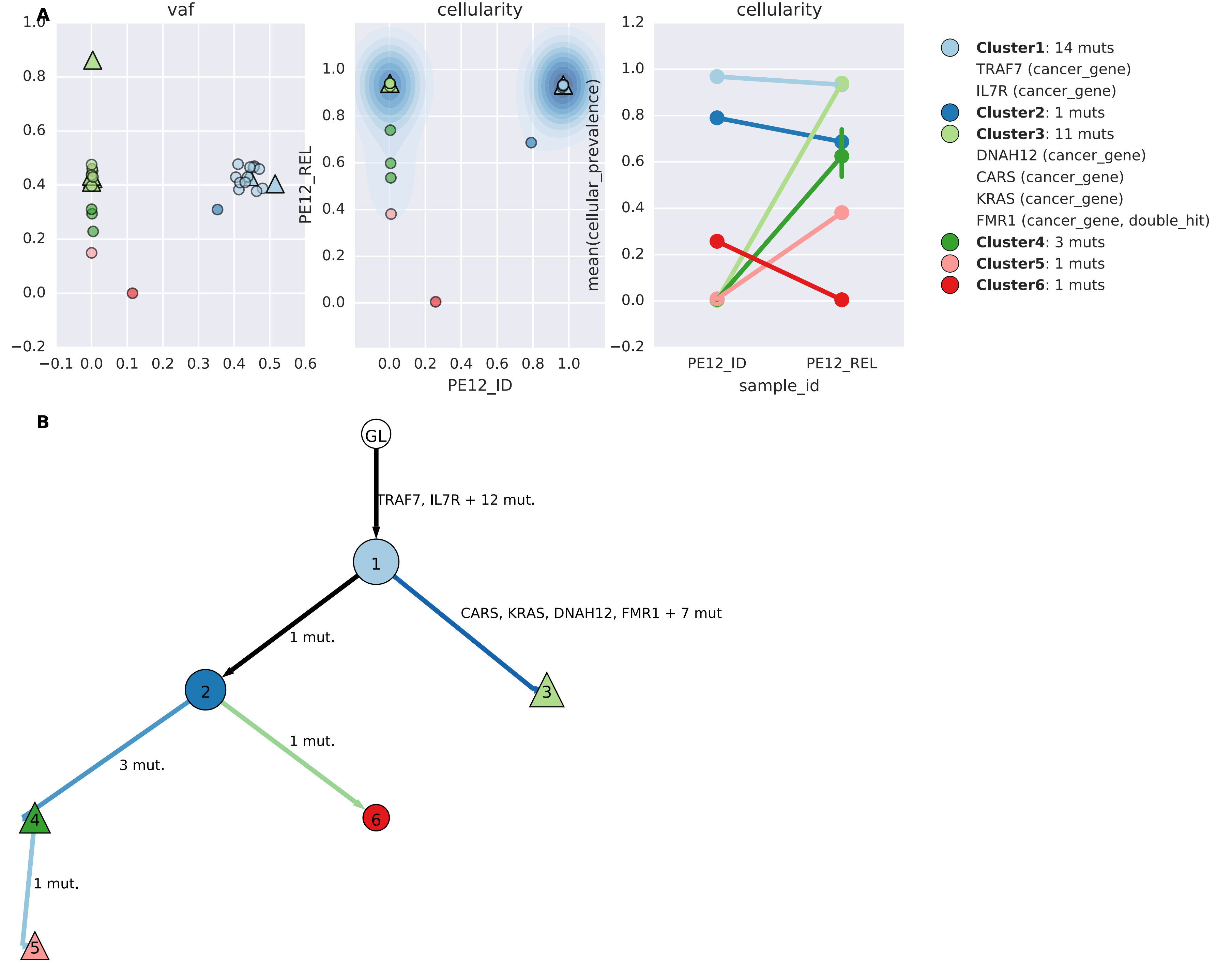
A
vaf
cellularity
cellularity
PE12_REL
PE12_ID
mean(cellular_prevalence)
sample_id
PE12_ID
PE12_REL
Cluster1: 14 muts
TRAF7 (cancer_gene)
IL7R (cancer_gene)
Cluster2: 1 muts
Cluster3: 11 muts
DNAH12 (cancer_gene)
CARS (cancer_gene)
KRAS (cancer_gene)
FMR1 (cancer_gene, double_hit)
Cluster4: 3 muts
Cluster5: 1 muts
Cluster6: 1 muts
B
GL
TRAF7, IL7R + 12 mut.
1
CARS, KRAS, DNAH12, FMR1 + 7 mut
1 mut.
2
3
1 mut.
3 mut.
4
6
1 mut.
5

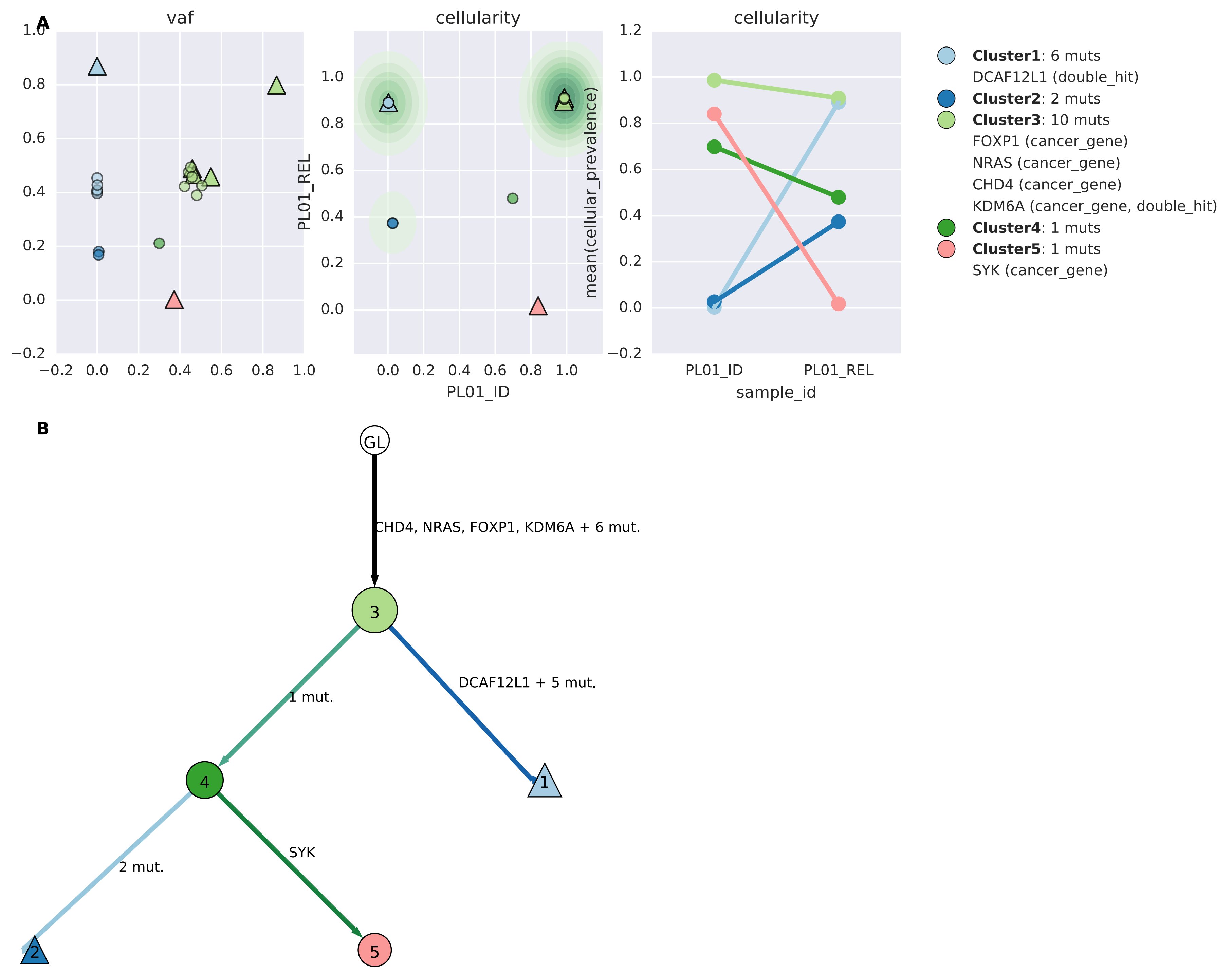
A
vaf
cellularity
PL01_REL
PL01_ID
cellularity
mean(cellular_prevalence)
PL01_ID
PL01_REL
sample_id
Cluster1: 6 muts
DCAF12L1 (double_hit)
Cluster2: 2 muts
Cluster3: 10 muts
FOXP1 (cancer_gene)
NRAS (cancer_gene)
CHD4 (cancer_gene)
KDM6A (cancer_gene, double_hit)
Cluster4: 1 muts
Cluster5: 1 muts
SYK (cancer_gene)
B
GL
CHD4, NRAS, FOXP1, KDM6A + 6 mut.
3
1 mut.
DCAF12L1 + 5 mut.
4
1
2 mut.
SYK
2
5

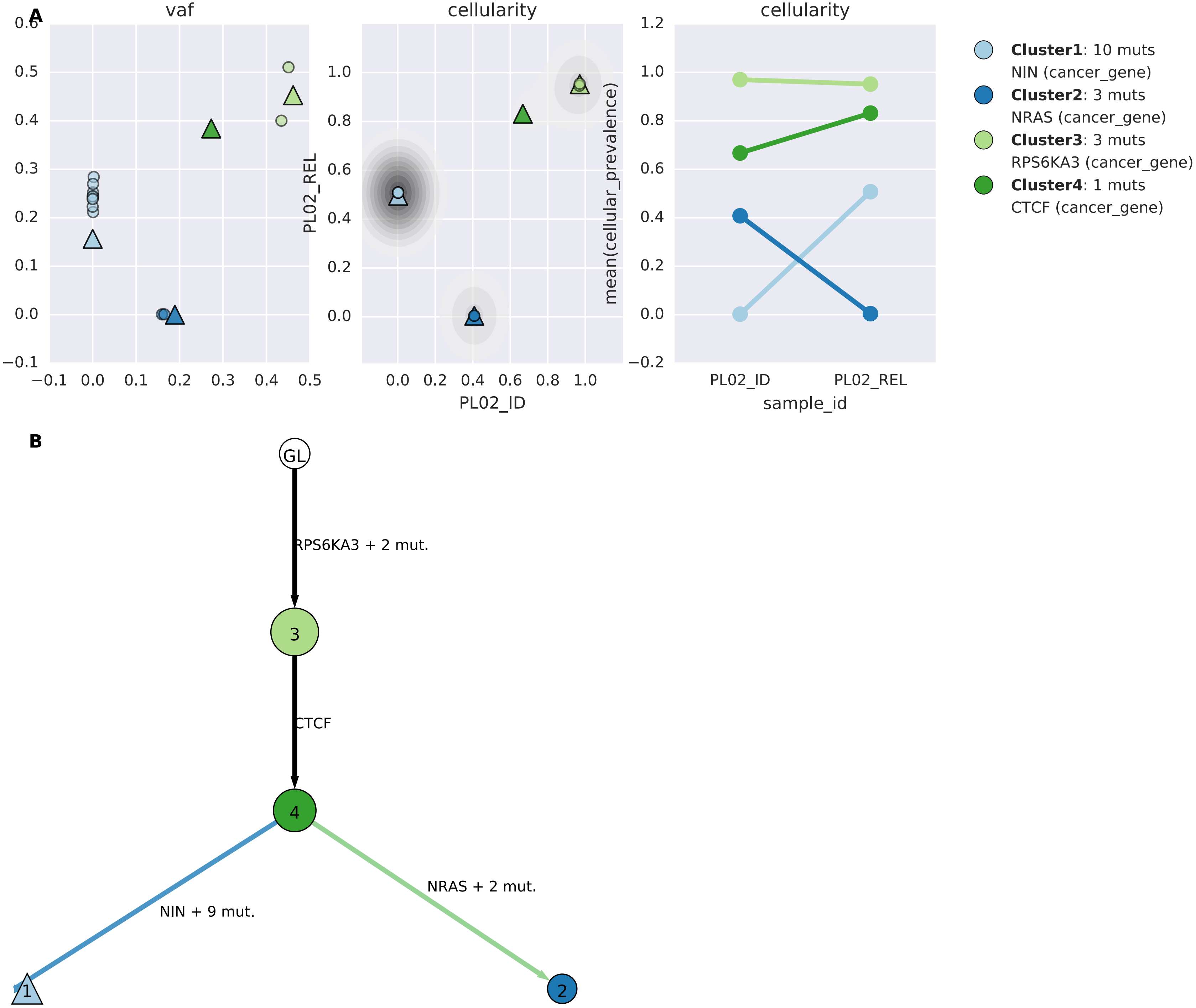
A
vaf
cellularity
cellularity
PL02_REL
PL02_ID
mean(cellular_prevalence)
PL02_ID
PL02_REL
sample_id
Cluster1: 10 muts
NIN (cancer_gene)
Cluster2: 3 muts
NRAS (cancer_gene)
Cluster3: 3 muts
RPS6KA3 (cancer_gene)
Cluster4: 1 muts
CTCF (cancer_gene)
B
GL
RPS6KA3 + 2 mut.
3
CTCF
4
NIN + 9 mut.
NRAS + 2 mut.
1
2

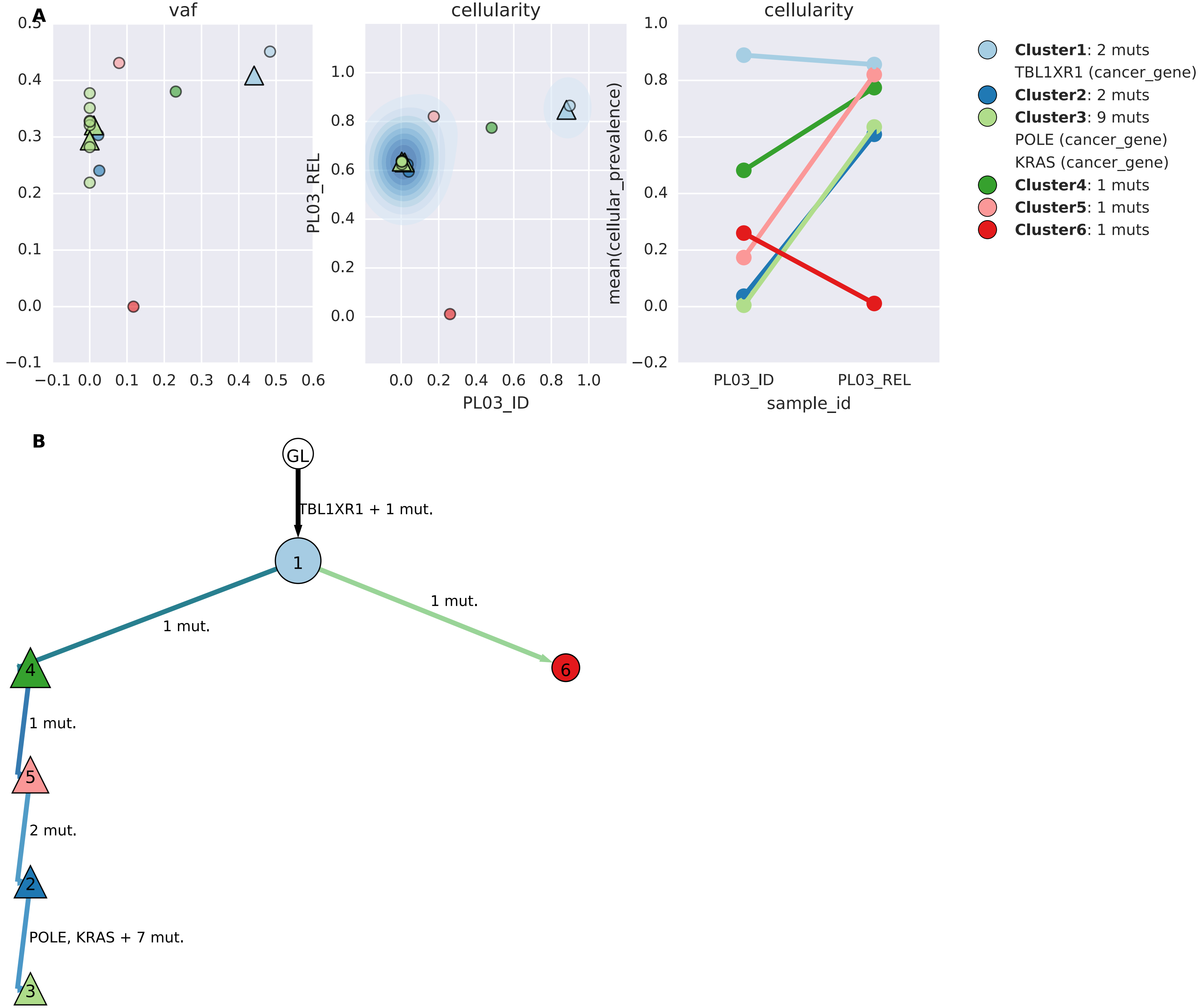

A
vaf
cellularity
PL03_REL
PL03_ID
cellularity
mean(cellular_prevalence)
PL03_ID
PL03_REL
sample_id
Cluster1: 2 muts
TBL1XR1 (cancer_gene)
Cluster2: 2 muts
Cluster3: 9 muts
POLE (cancer_gene)
KRAS (cancer_gene)
Cluster4: 1 muts
Cluster5: 1 muts
Cluster6: 1 muts
B
GL
TBL1XR1 + 1 mut.
1
1 mut.
1 mut.
4
6
1 mut.
5
2 mut.
2
POLE, KRAS + 7 mut.
3

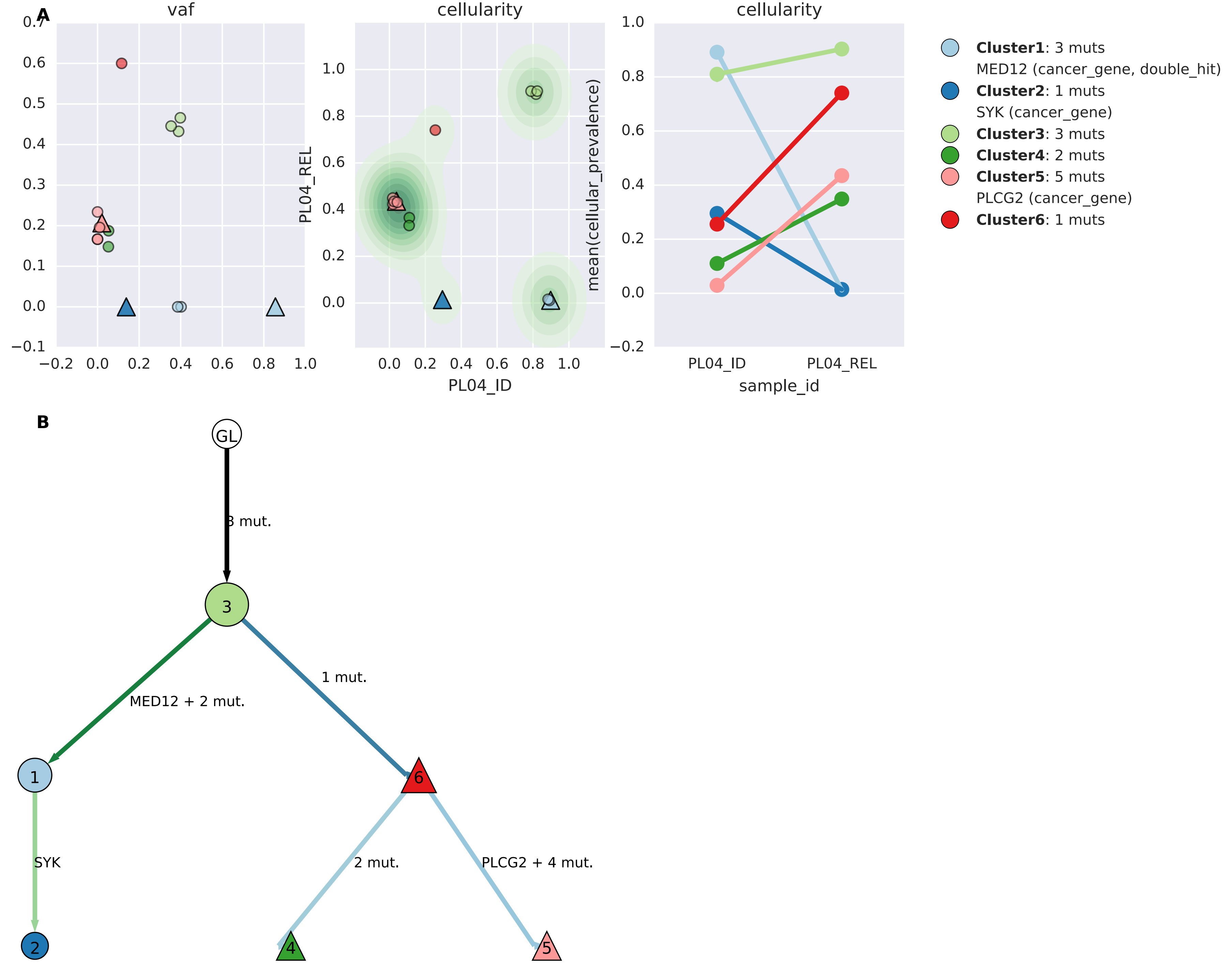
A
vaf
cellularity
PL04_REL
PL04_ID
cellularity
mean(cellular_prevalence)
PL04_ID
PL04_REL
sample_id
Cluster1: 3 muts
MED12 (cancer_gene, double_hit)
Cluster2: 1 muts
SYK (cancer_gene)
Cluster3: 3 muts
Cluster4: 2 muts
Cluster5: 5 muts
PLCG2 (cancer_gene)
Cluster6: 1 muts
B
GL
3 mut.
3
MED12 + 2 mut.
1 mut.
1
6
SYK
2 mut.
PLCG2 + 4 mut.
2
4
5

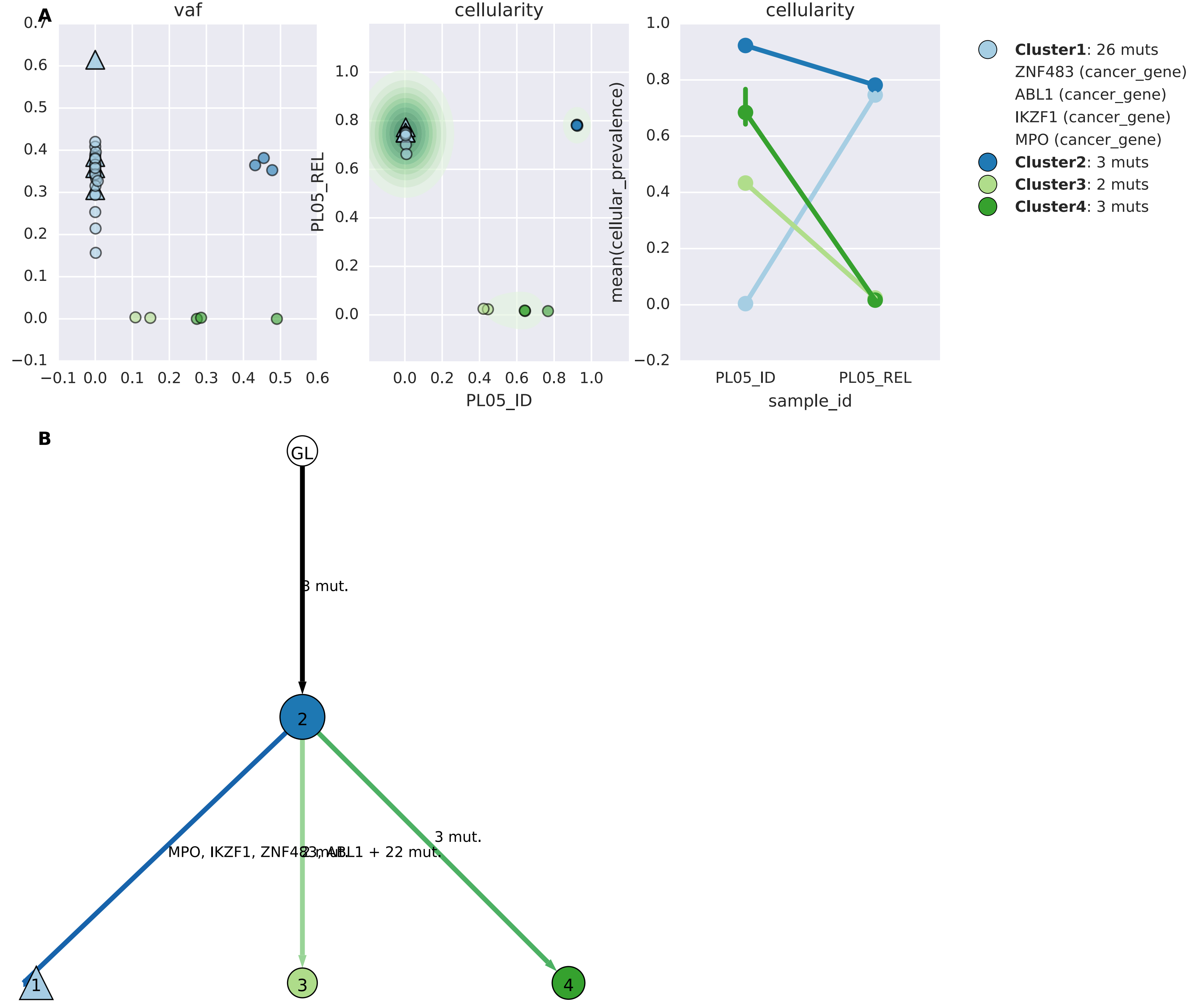

A
vaf
cellularity
PL05_REL
PL05_ID
cellularity
mean(cellular_prevalence)
PL05_ID
PL05_REL
sample_id
Cluster1: 26 muts
ZNF483 (cancer_gene)
ABL1 (cancer_gene)
IKZF1 (cancer_gene)
MPO (cancer_gene)
Cluster2: 3 muts
Cluster3: 2 muts
Cluster4: 3 muts
B
GL
3 mut.
2
MPO, IKZF1, ZNF483, ABL1 + 22 mut.
2 mut.
3 mut.
1
3
4

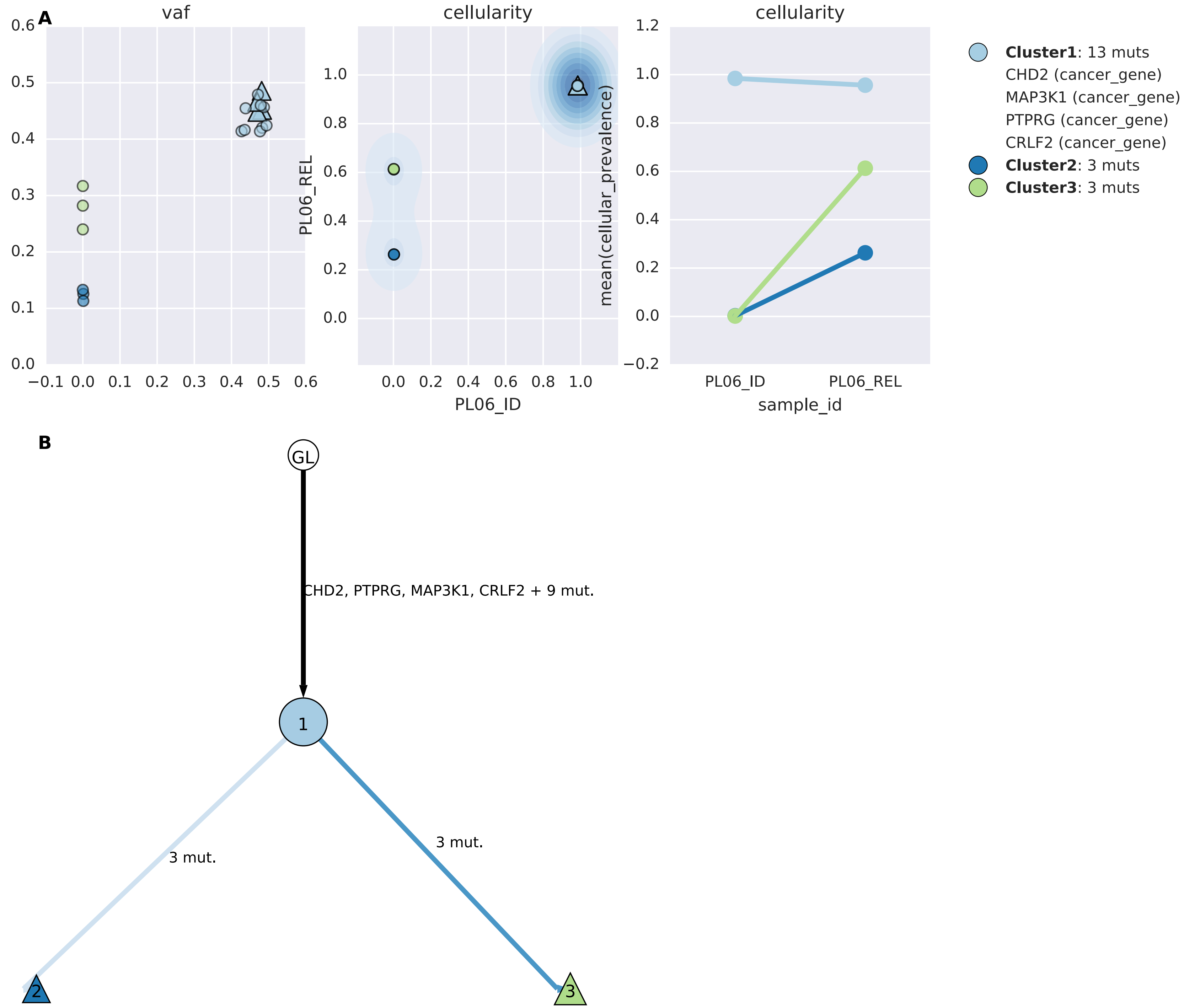

A
vaf
cellularity
cellularity
PL06_REL
PL06_ID
mean(cellular_prevalence)
sample_id
PL06_ID
PL06_REL
Cluster1: 13 muts
CHD2 (cancer_gene)
MAP3K1 (cancer_gene)
PTPRG (cancer_gene)
CRLF2 (cancer_gene)
Cluster2: 3 muts
Cluster3: 3 muts
B
GL
CHD2, PTPRG, MAP3K1, CRLF2 + 9 mut.
1
3 mut.
3 mut.
2
3

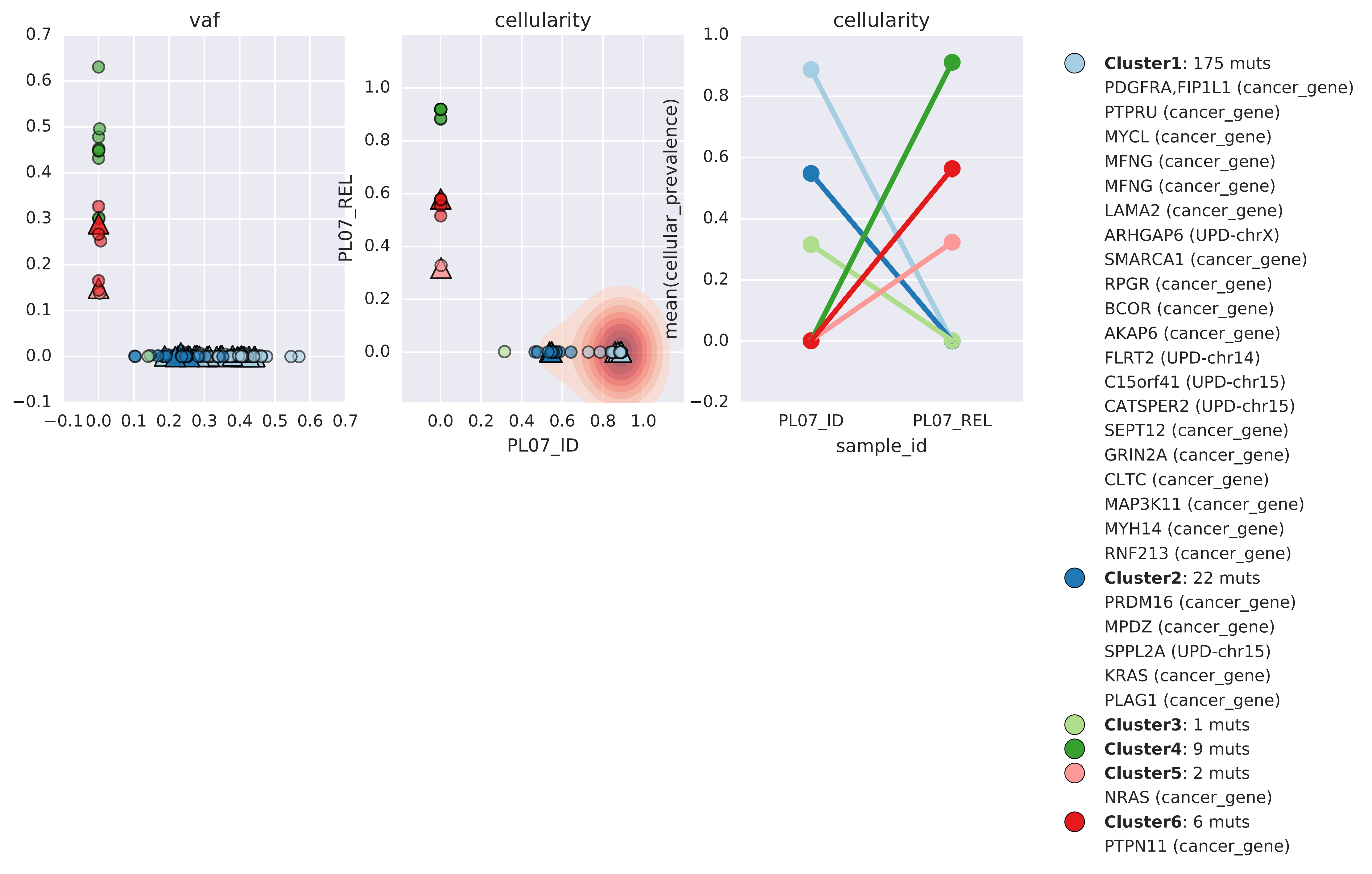
vaf
cellularity
cellularity
PL07_REL
PL07_ID
mean(cellular_prevalence)
PL07_ID
PL07_REL
sample_id
Cluster1: 175 muts
PDGFRA,FIP1L1 (cancer_gene)
PTPRU (cancer_gene)
MYCL (cancer_gene)
MFNG (cancer_gene)
MFNG (cancer_gene)
LAMA2 (cancer_gene)
ARHGAP6 (UPD-chrX)
SMARCA1 (cancer_gene)
RPGR (cancer_gene)
BCOR (cancer_gene)
AKAP6 (cancer_gene)
FLRT2 (UPD-chr14)
C15orf41 (UPD-chr15)
CATSPER2 (UPD-chr15)
SEPT12 (cancer_gene)
GRIN2A (cancer_gene)
CLTC (cancer_gene)
MAP3K11 (cancer_gene)
MYH14 (cancer_gene)
RNF213 (cancer_gene)
Cluster2: 22 muts
PRDM16 (cancer_gene)
MPDZ (cancer_gene)
SPPL2A (UPD-chr15)
KRAS (cancer_gene)
PLAG1 (cancer_gene)
Cluster3: 1 muts
Cluster4: 9 muts
Cluster5: 2 muts
NRAS (cancer_gene)
Cluster6: 6 muts
PTPN11 (cancer_gene)

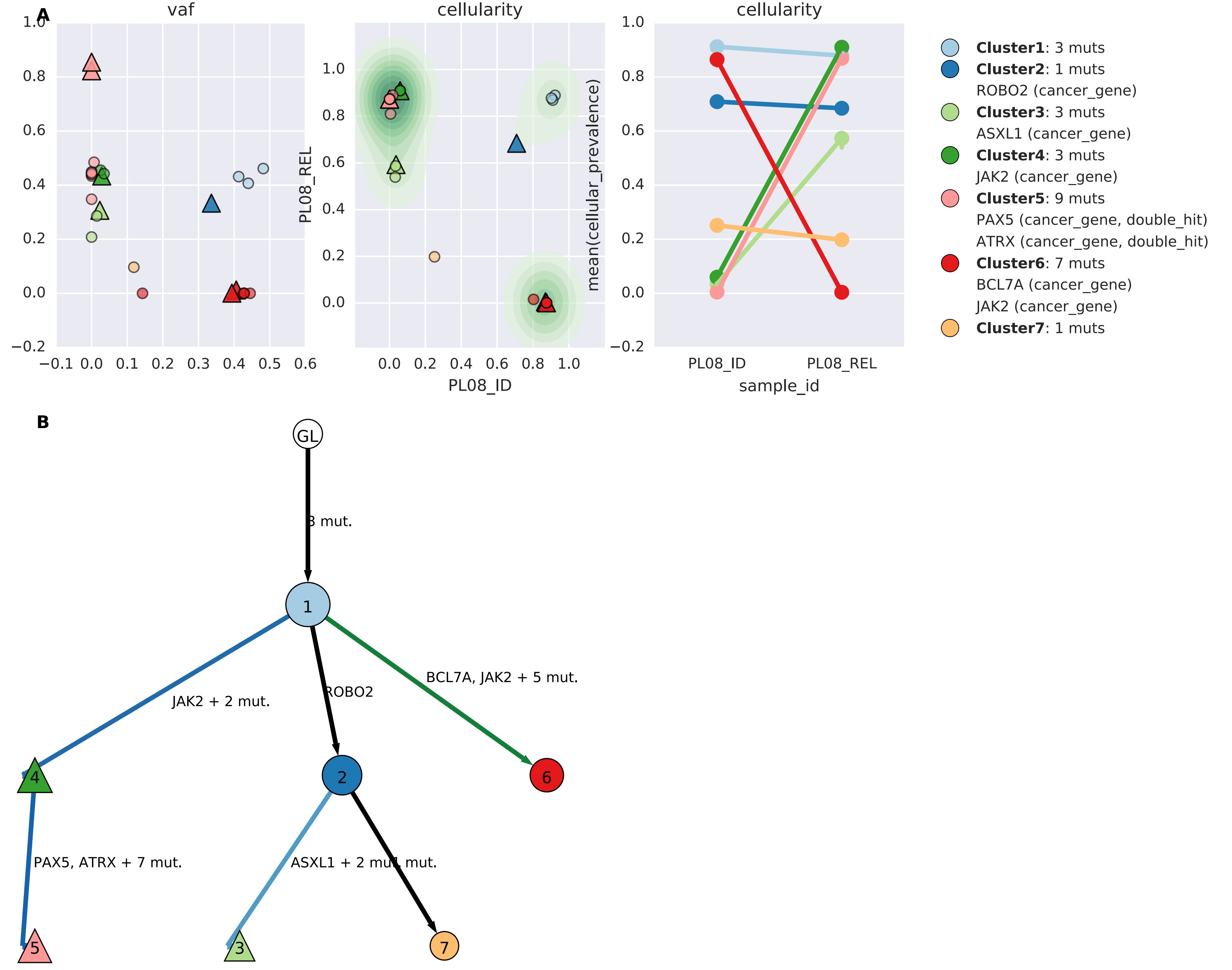
A
vaf
cellularity
PL08_REL
PL08_ID
cellularity
mean(cellular_prevalence)
PL08_ID
PL08_REL
sample_id
Cluster1: 3 muts
Cluster2: 1 muts
ROBO2 (cancer_gene)
Cluster3: 3 muts
ASXL1 (cancer_gene)
Cluster4: 3 muts
JAK2 (cancer_gene)
Cluster5: 9 muts
PAX5 (cancer_gene, double_hit)
ATRX (cancer_gene, double_hit)
Cluster6: 7 muts
BCL7A (cancer_gene)
JAK2 (cancer_gene)
Cluster7: 1 muts
B
GL
3 mut.
1
JAK2 + 2 mut.
ROBO2
BCL7A, JAK2 + 5 mut.
4
2
6
PAX5, ATRX + 7 mut.
ASXL1 + 2 mut.
mut.
5
3
7

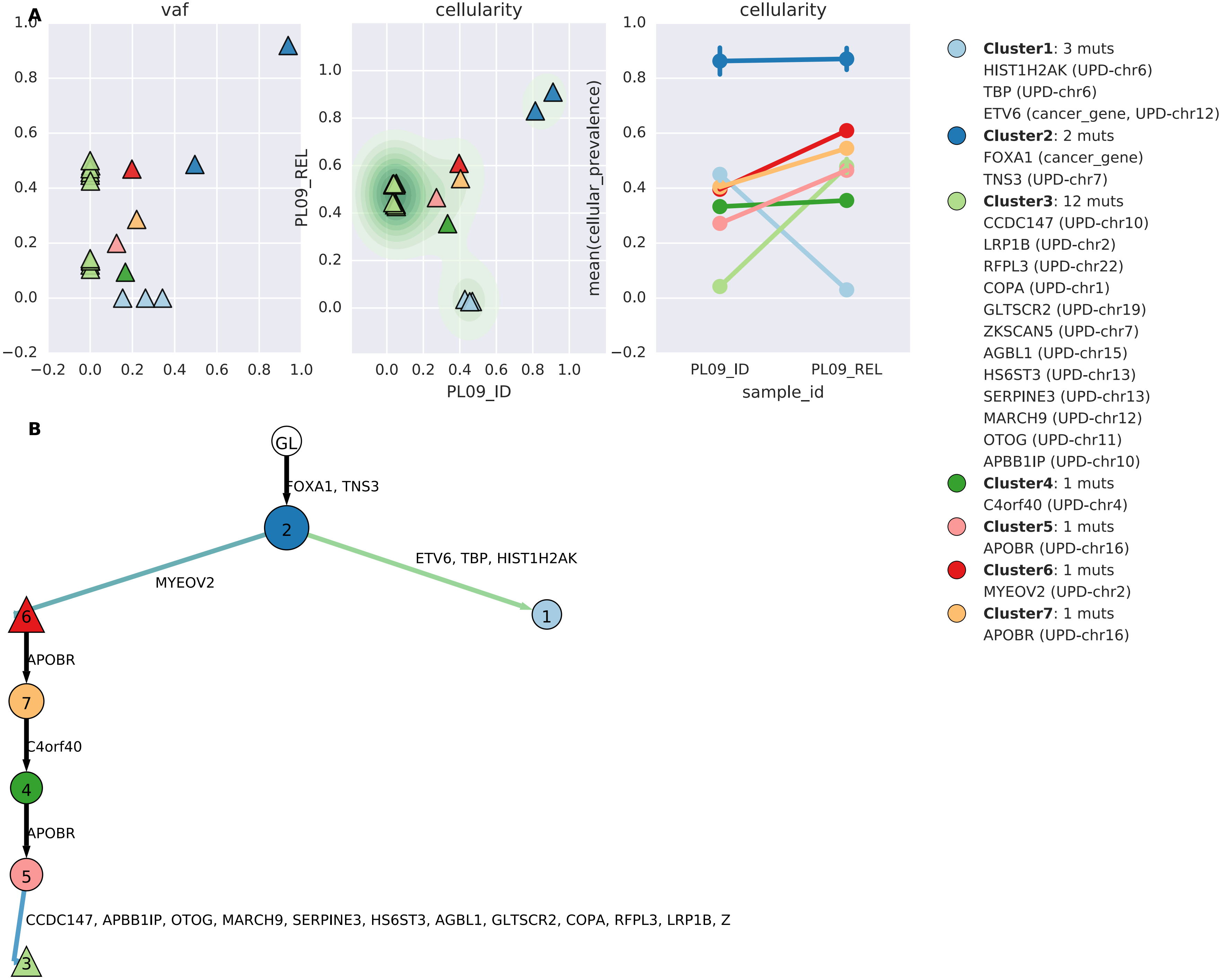
A
vaf
cellularity
cellularity
PL09_REL
PL09_ID
mean(cellular_prevalence)
sample_id
PL09_ID
PL09_REL
Cluster1: 3 muts
HIST1H2AK (UPD-chr6)
TBP (UPD-chr6)
ETV6 (cancer_gene, UPD-chr12)
Cluster2: 2 muts
FOXA1 (cancer_gene)
TNS3 (UPD-chr7)
Cluster3: 12 muts
CCDC147 (UPD-chr10)
LRP1B (UPD-chr2)
RFPL3 (UPD-chr22)
COPA (UPD-chr1)
GLTSCR2 (UPD-chr19)
ZKSCAN5 (UPD-chr7)
AGBL1 (UPD-chr15)
HS6ST3 (UPD-chr13)
SERPINE3 (UPD-chr13)
MARCH9 (UPD-chr12)
OTOG (UPD-chr11)
APBB1IP (UPD-chr10)
Cluster4: 1 muts
C4orf40 (UPD-chr4)
Cluster5: 1 muts
APOBR (UPD-chr16)
Cluster6: 1 muts
MYEOV2 (UPD-chr2)
Cluster7: 1 muts
APOBR (UPD-chr16)
B
GL
FOXA1, TNS3
2
ETV6, TBP, HIST1H2AK
MYEOV2
1
6
APOBR
7
C4orf40
4
APOBR
5
CCDC147, APBB1IP, OTOG, MARCH9, SERPINE3, HS6ST3, AGBL1, GLTSCR2, COPA, RFPL3, LRP1B, Z
3

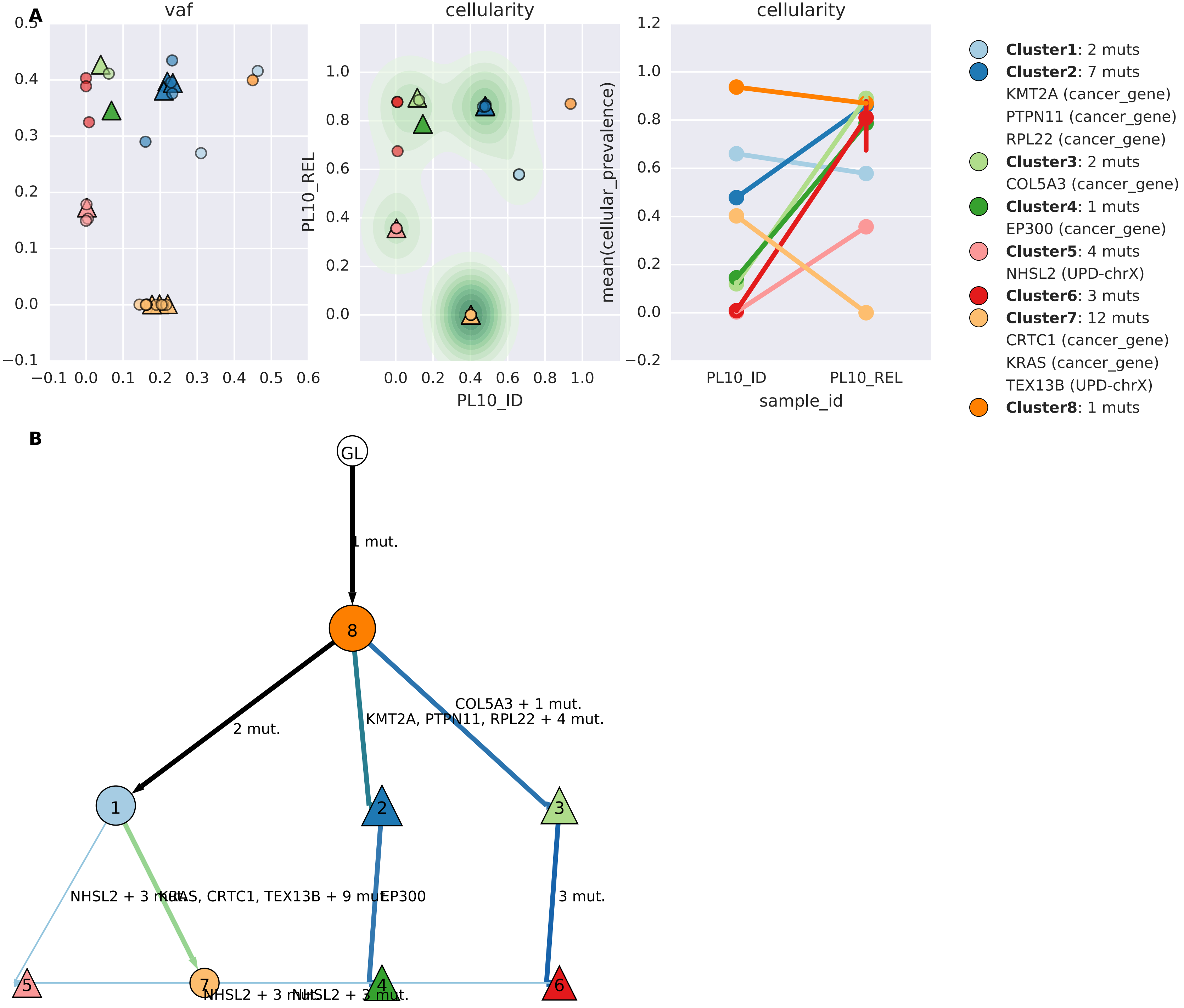
A
vaf
cellularity
PL10_REL
PL10_ID
cellularity
mean(cellular_prevalence)
PL10_ID
PL10_REL
sample_id
Cluster1: 2 muts
Cluster2: 7 muts
KMT2A (cancer_gene)
PTPN11 (cancer_gene)
RPL22 (cancer_gene)
Cluster3: 2 muts
COL5A3 (cancer_gene)
Cluster4: 1 muts
EP300 (cancer_gene)
Cluster5: 4 muts
NHSL2 (UPD-chrX)
Cluster6: 3 muts
Cluster7: 12 muts
CRTC1 (cancer_gene)
KRAS (cancer_gene)
TEX13B (UPD-chrX)
Cluster8: 1 muts
B
GL
1 mut.
8
2 mut.
KMT2A, PTPN11, RPL22 + 4 mut.
COL5A3 + 1 mut.
1
2
3
NHSL2 + 3 mut.
KRAS, CRTC1, TEX13B + 9 mut.
EP300
3 mut.
5
7
4
6
NHSL2 + 3 mut.
NHSL2 + 3 mut.

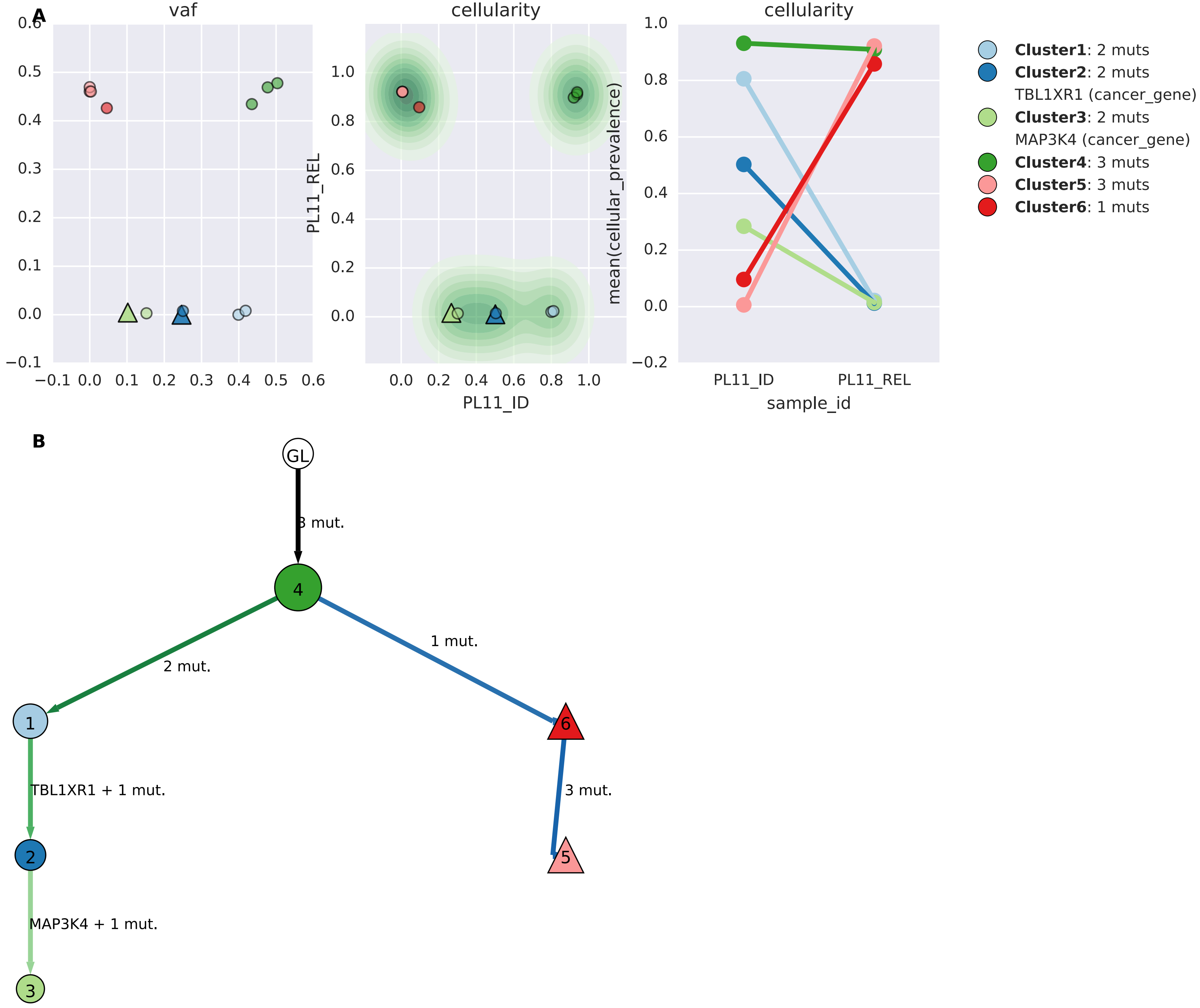
A
vaf
cellularity
PL11_REL
PL11_ID
cellularity
mean(cellular_prevalence)
PL11_ID
PL11_REL
sample_id
Cluster1: 2 muts
Cluster2: 2 muts
TBL1XR1 (cancer_gene)
Cluster3: 2 muts
MAP3K4 (cancer_gene)
Cluster4: 3 muts
Cluster5: 3 muts
Cluster6: 1 muts
B
GL
3 mut.
4
2 mut.
1 mut.
1
6
TBL1XR1 + 1 mut.
3 mut.
2
5
MAP3K4 + 1 mut.
3

A

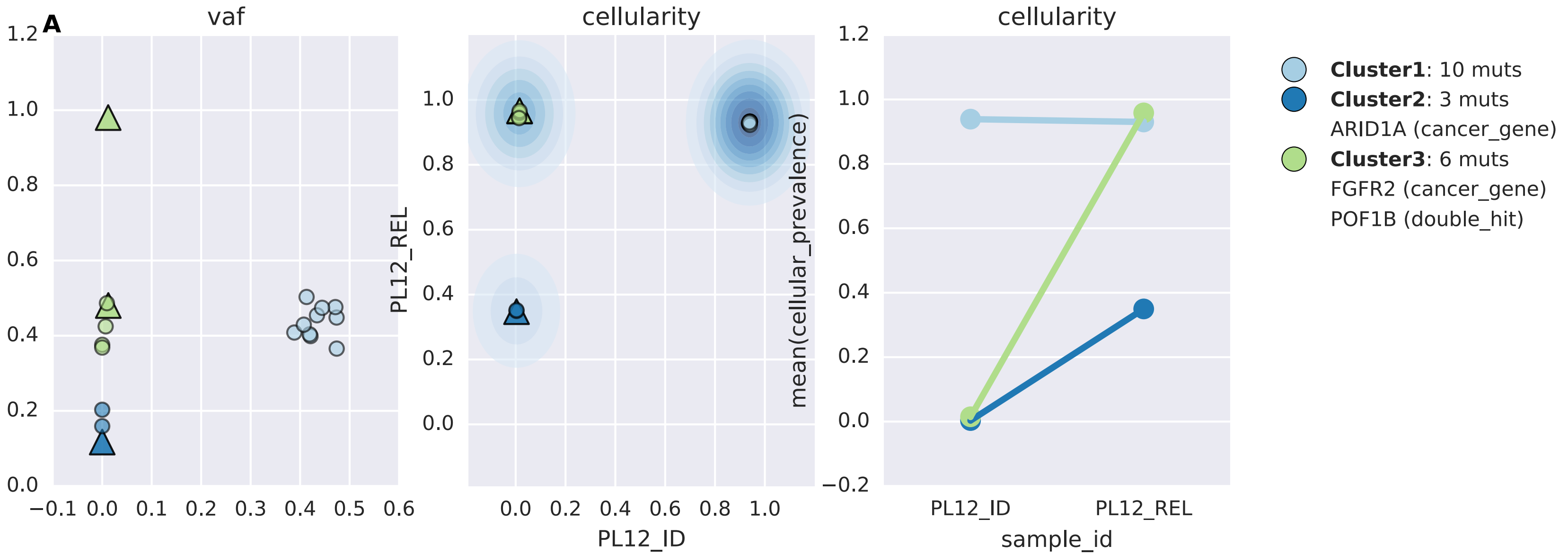
vaf
cellularity
cellularity
PL12_REL
PL12_ID
mean(cellular_prevalence)
sample_id
PL12_ID
PL12_REL
Cluster1: 10 muts
Cluster2: 3 muts
ARID1A (cancer_gene)
Cluster3: 6 muts
FGFR2 (cancer_gene)
POF1B (double_hit)

B

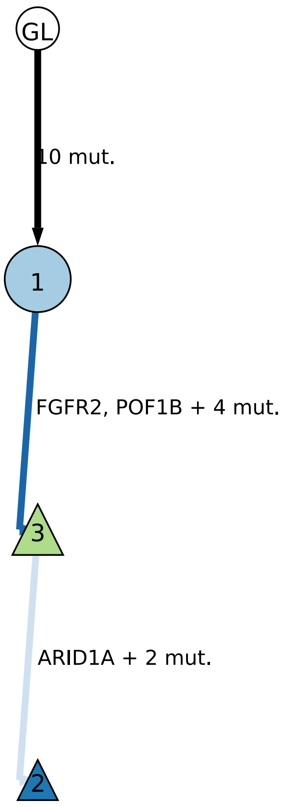
GL
10 mut.
1
FGFR2, POF1B + 4 mut.
3
ARID1A + 2 mut.
2

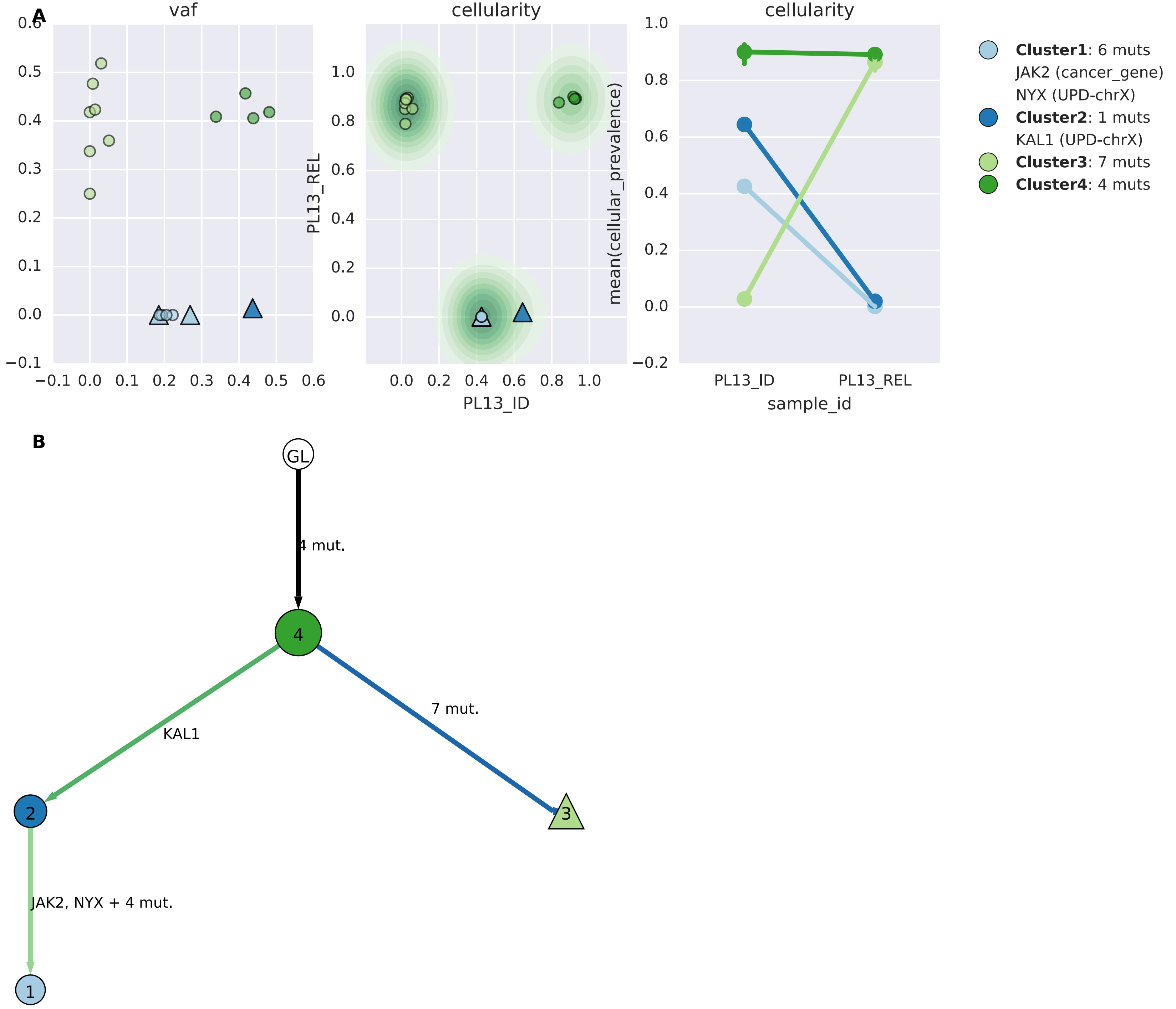

A
vaf
cellularity
cellularity
PL13_REL
PL13_ID
mean(cellular_prevalence)
sample_id
PL13_ID
PL13_REL
Cluster1: 6 muts
JAK2 (cancer_gene)
NYX (UPD-chrX)
Cluster2: 1 muts
KAL1 (UPD-chrX)
Cluster3: 7 muts
Cluster4: 4 muts
B
GL
4 mut.
4
KAL1
7 mut.
2
3
JAK2, NYX + 4 mut.
1

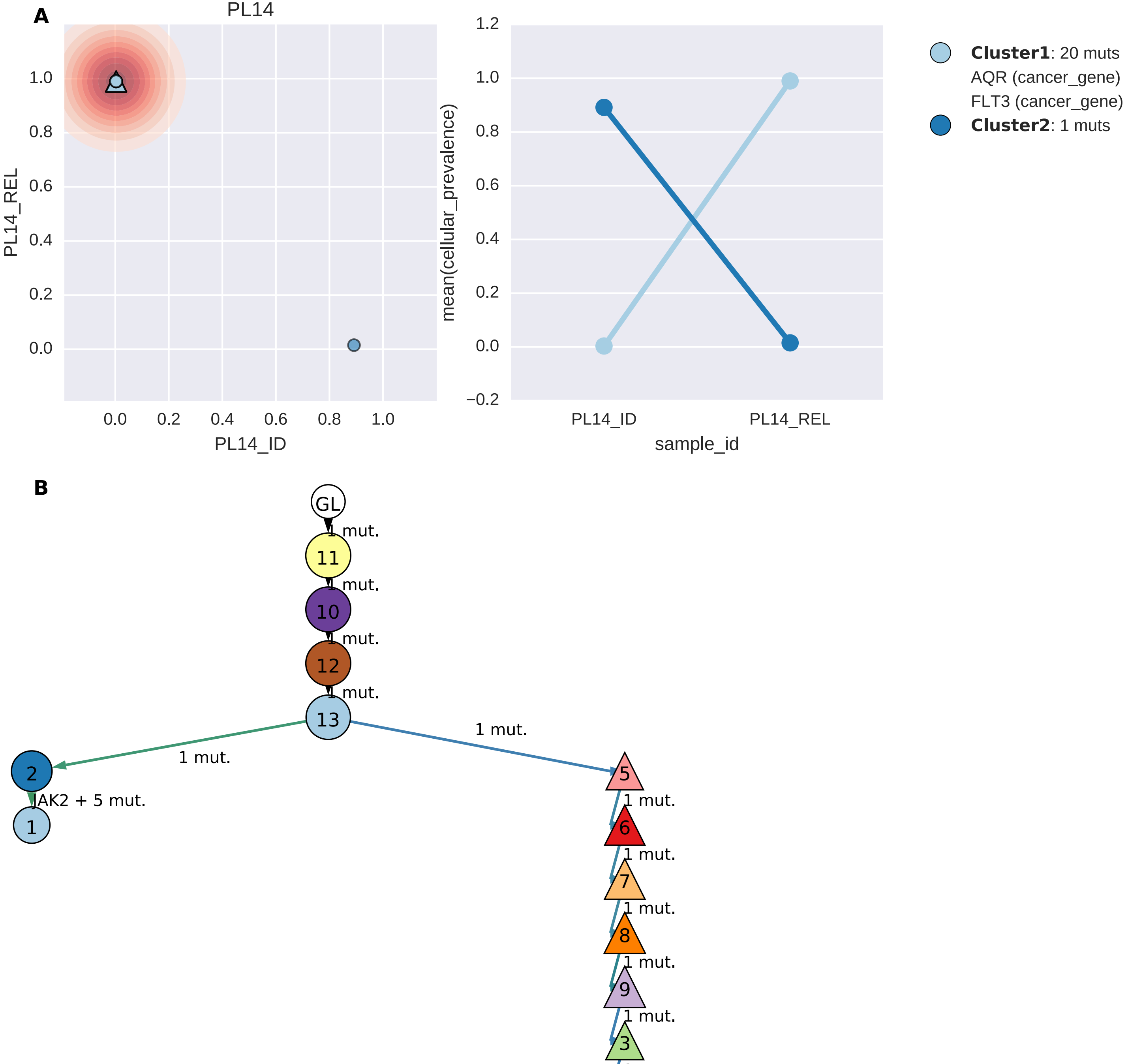

A
PL14
1.0
0.8
0.6
0.4
0.2
0.0
PL14_REL
0.0
0.2
0.4
0.6
0.8
1.0
PL14_ID
1.2
1.0
0.8
0.6
0.4
0.2
0.0
−0.2
mean(cellular_prevalence)
PL14_ID
PL14_REL
sample_id
Cluster1: 20 muts
AQR (cancer_gene)
FLT3 (cancer_gene)
Cluster2: 1 muts
B
GL
1 mut.
11
1 mut.
10
1 mut.
12
1 mut.
13
1 mut.
2
JAK2 + 5 mut.
1
1 mut.
5
1 mut.
6
1 mut.
7
1 mut.
8
1 mut.
9
1 mut.
3

Figure S6: This figure shows the clonal evolution trees between ID and REL for all patients accordingly to Figure S5. Each page contains a plot for a patient, subdivided into frequency plots (a) and the Schism evolutionary tree (b). (a) The frequency plots represent, from left to right the variant allele frequency of the mutations, the cellularities as calculated by PyClone and lastly the clone-specific mean cellularities as a line plot between ID and REL. The triangles in the VAF and cellularity plots represent the highlighted genes in the legend. These highlighted genes represent genes in our curated cancer gene (Supplementary Table S10) list and genes of which one copy has been lost according to the CNA analysis. (b) The GL node represents the germline, the starting point. The greener the arrow, the more specific to ID, the bluer the arrow the more specific to REL. The darker the luminosity reflects the higher cellularity of the clone.

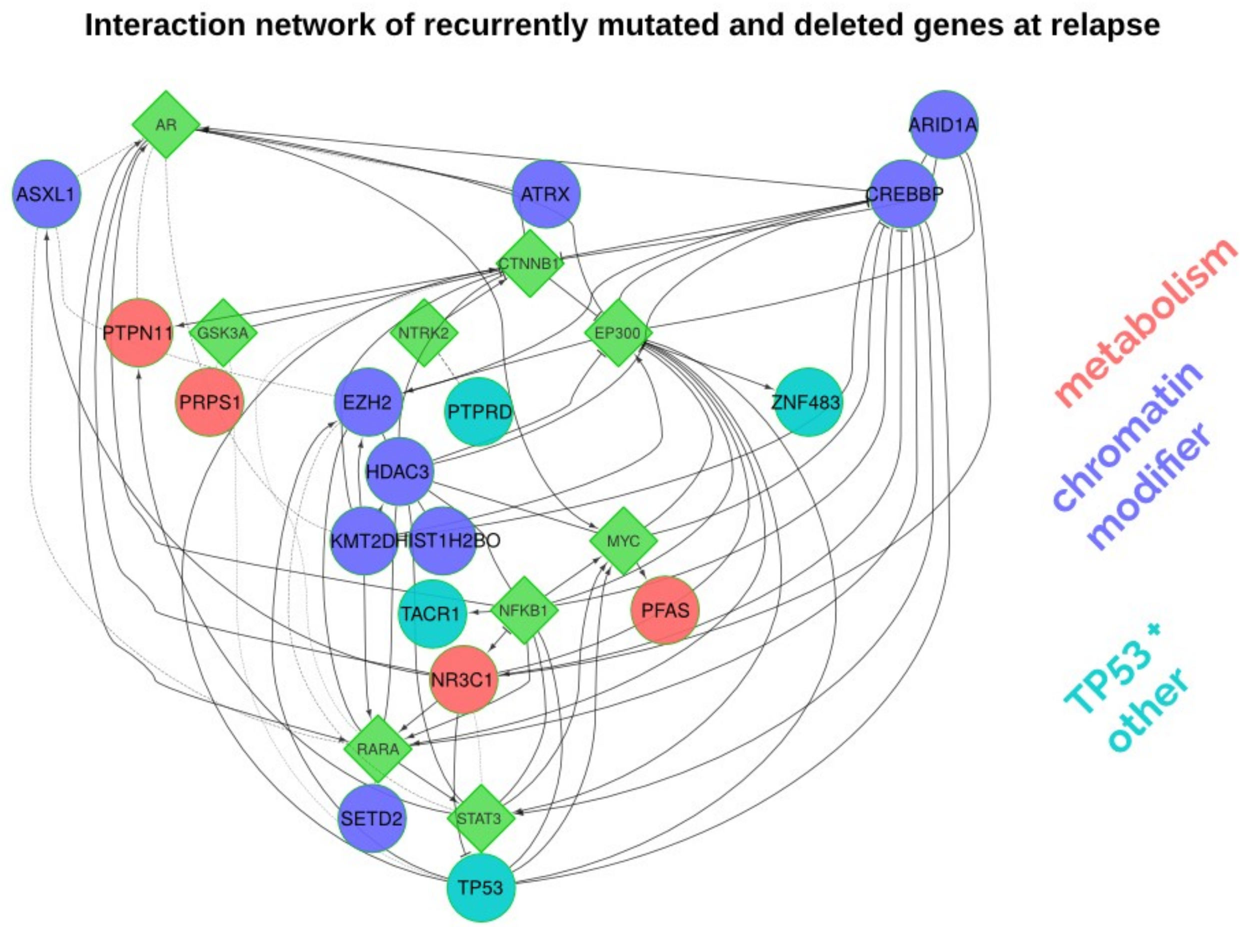

Figure S7: Relapse-mutation protein-protein interaction network. The network shows the protein-protein interaction of the recurrently mutated and deleted genes at relapse as retrieved from Reactome FIViz Cytoscape plugin. Diamond-shaped nodes are so-called linker genes, connecting genes from the original query set. Solid lines are experimentally validated and dashed lines represent predicted interactions. The color of the circular nodes represents the class according to (Figure 2).

Protein and transcript expression changes froM ID to REL

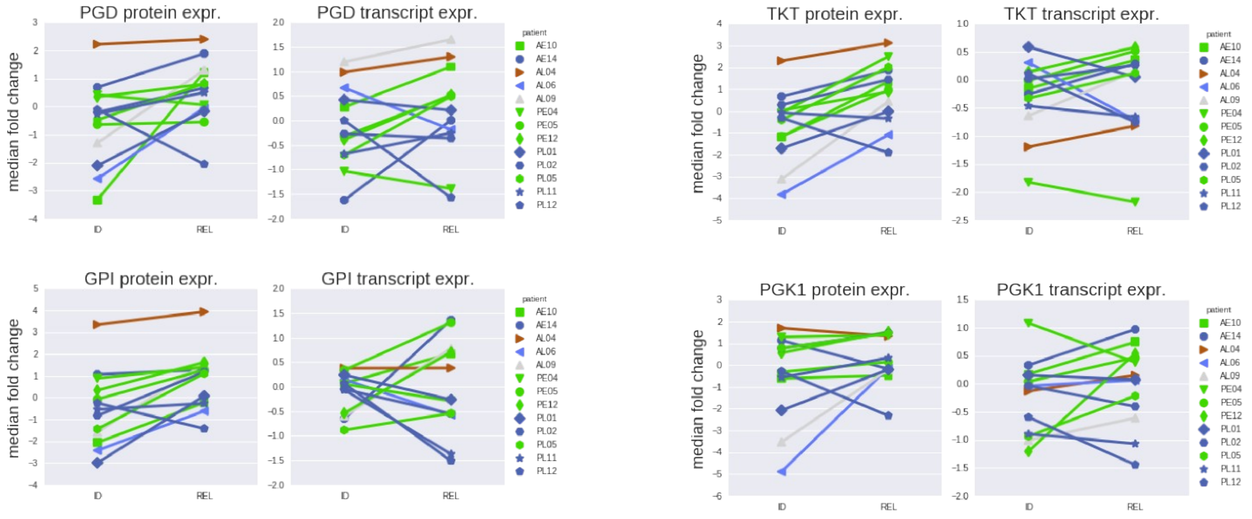

Figure S8: The expression of the protein products and mRNA transcripts of PGD, GPI, TKT and PGK1 at ID and REL of four metabolic genes.

Top 10 pathways of protein expression enriched in Ph-like relapse samples

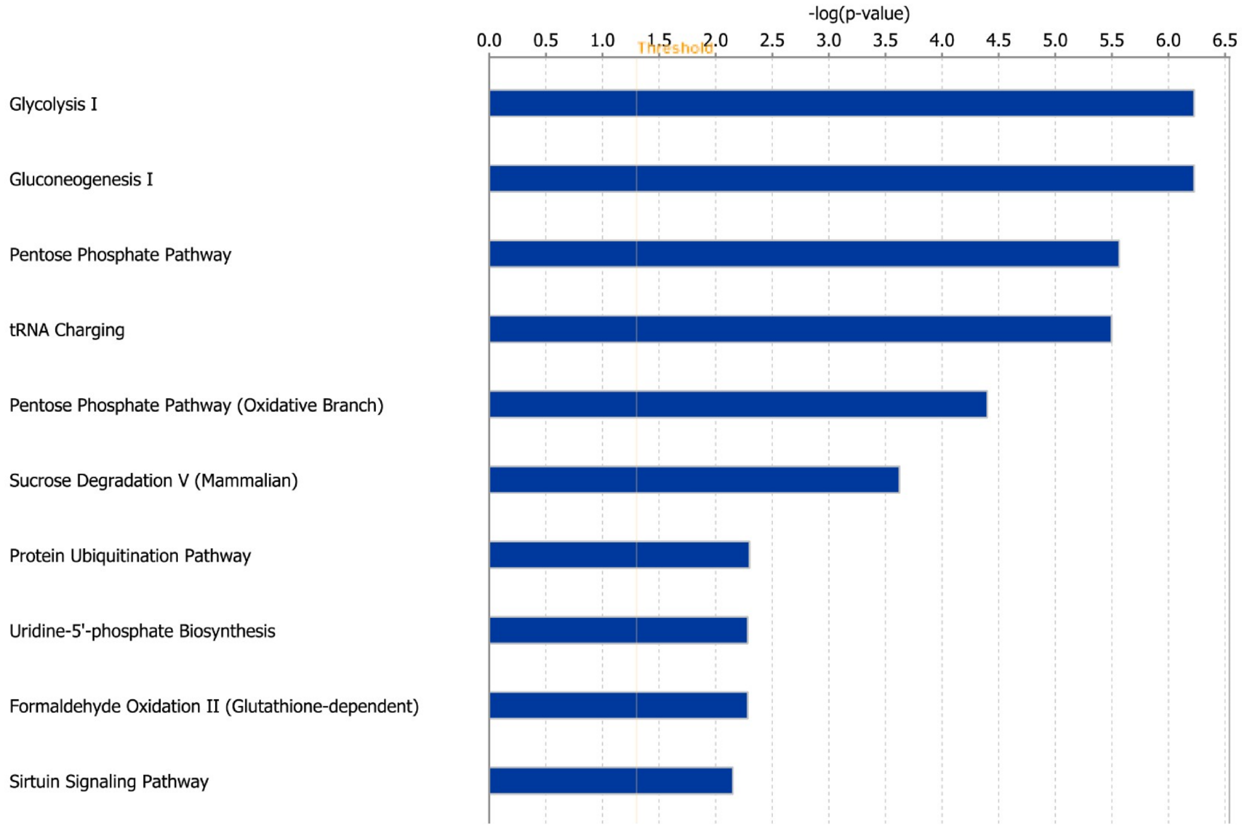

Figure S9: Protein expression and pathway enrichment in the Ph-like relapse samples. The bar plot shows the top 10 ingenuity pathways for differentially expressed proteins in Ph-like relapse samples.
